# A physically consistent dataset of water-energy-carbon fluxes across the Soil-Plant-Atmosphere Continuum

**DOI:** 10.1038/s41597-025-05386-x

**Published:** 2025-07-04

**Authors:** Yunfei Wang, Yijian Zeng, Fakhereh Alidoost, Bart Schilperoort, Zengjing Song, Danyang Yu, Enting Tang, Qianqian Han, Zhunqiao Liu, Xiongbiao Peng, Chao Zhang, Bas Retsios, Serkan Girgin, Xiaoliang Lü, Qiting Zuo, Huanjie Cai, Qiang Yu, Christiaan van der Tol, Zhongbo Su

**Affiliations:** 1 State Key Laboratory of Soil and Water Conservation and Desertification Control, Northwest Agriculture and Forestry University, Yangling, 712100 China; 2 Faculty of Geo-Information Science and Earth Observation, University of Twente, Enschede, 7500 AE The Netherlands; 3 School of Water Conservancy and Transportation, Zhengzhou University, Zhengzhou, 450001 China; 4 Netherlands eScience Center, Amsterdam, 1098 XH The Netherlands; 5 College of Water Resources and Architectural Engineering, Northwest Agriculture and Forestry University, Yangling, 712100 China; 6 College of Hydraulic Science and Engineering, Yangzhou University, Yangzhou, 225009 China

**Keywords:** Hydrology, Ecological modelling

## Abstract

Hight-quality and Long-term measurements of land-atmosphere fluxes are vital for climate monitoring and Land Surface models (LSMs) benchmarking. Eddy covariance provides key *in-situ* data for theory and LSMs evaluation, but most flux towers lack continuous soil-plant-atmosphere measurements. Here, we present a long-term global dataset of water, energy and carbon fluxes, along with the corresponding above and below-ground hydrological, photosynthetic, and radiative data derived from the STEMMUS-SCOPE model simulations at 170 sites. *In-situ* observed fluxes data from PLUMBER2 and soil moisture (SM) data from FLUXNET2015 are employed to validate the effectiveness of the STEMMUS-SCOPE dataset. Results demonstrate that, without site-specific model tuning or calibration, and driven solely by global parameters and forcing datasets, simulated net radiation, latent heat flux, sensible heat flux, gross primary production, net ecosystem exchange, and SM datasets consistently agree with available *in-situ* measurements (median KGE: −0.03 to 0.80; median R^2^: 0.46 to 0.97; median rRMSE: 4.09% to 29.11%). This dataset supplements the existing ecosystem flux and SM network, enhancing our understanding of ecosystem functioning.

## Background & Summary

Climate change modifies the interactions between the land surface and the atmosphere, significantly affecting global eco-hydrological processes^1–5^. Understanding its impacts necessitates long-term and physically consistent *in-situ* observations of eco-hydrological variables, including soil moisture (SM), gross primary production (GPP), and evapotranspiration (ET)^6^. However, the reliability of sensors and the difficulty and costs associated with maintaining representative sites across different biomes hinder the availability of physically consistent long-term datasets of water-energy-carbon fluxes across the Soil-Plant-Atmosphere Continuum (SPAC) at a global scale^7^. Therefore, data reliability remains one of the most elusive and significant sources of uncertainty in understanding the eco-hydrological cycle^8^.

Soil moisture plays a crucial role in regulating hydrological processes such as runoff and infiltration, as well as land-atmosphere interactions including ET and GPP^9–12^. While *in-situ* sensors can accurately measure SM, their limited spatial coverage and temporal frequency restrict their applicability to the local scale. Despite continuous global surface SM products retrievable from satellite data^13–15^, their accuracy and resolutions remain insufficient to fully comprehend its role in eco-hydrological processes. Another practical method in obtaining reliable long-term SM data is through the utility of process-based models driven by long-term biological and meteorological observations. The simulated SM theoretically mirrors the spatial-temporal variations observed by the *in-situ* and satellite sensors^16–18^. However, due to the specific model physics and structure, as well as uncertainty in parameters and meteorological forcings, SM simulations generally exhibit a high degree of uncertainty^19,20^.

The FLUXNET2015 dataset provides ecosystem-scale data on carbon, water, and energy fluxes between the land surface and the atmosphere, along with corresponding meteorological (e.g. radiation, air temperature, and wind speed) and biological measurements (e.g. leaf area index) worldwide (212 sites in total, over 1500 site-years)^21^. The FLUXNET2015 dataset addresses the limitations of prior datasets by implementing a standardized quality control and processing approach to enhance consistency and comparability across sites. However, the long durations of gap-fillied data may lead to inaccurate diurnal and/or seasonal cycles^7,22^. Furthermore, the gap-filled meteorological variables pose challenges in model applications, not only introducing biases in current time steps but also impacting future model predictions^23^. Consequently, using the FLUXNET2015 dataset to assess the performance of land surface models still faces challenges.

To address this issue, the second phase of the Protocol for the Analysis of Land Surface Models (PALS) Land Surface Model Benchmarking Evaluation Project (PLUMBER2) presented a dataset comprising 170 eddy covariance based flux sites, primarily sourced from FLUXNET2015, with additional contributions from the La Thuile and OzFlux networks^23^. PLUMBER2 includes 11 vegetation types and a diverse range of climate conditions. The earliest available *in-situ* measurements date back to 1992, with data available for most sites until 2014 (some sites until 2018). PLUMBER2 site data covers a period ranging from 1 to 21 years, totalling over 1000 site-years. It includes quality-controlled, fully gap-filled meteorological variables for driving land surface models, as well as a comprehensive set of *in-situ* measurements of water-energy-carbon fluxes^23^. Although PLUMBER2 addresses numerous limitations of FLUXNET2015, it does not include measurements of soil states (e.g., soil moisture and temperature). Although some of the FLUXNET2015 sites have multi-depth observations of SM and temperature, the root zone moisture information is not available, and it is difficult to conduct further analysis directly due to data scarcity and lack of data quality information. Last but not least, due to similar reasons, for gap-filling of flux data the PLUMBER2 employed the empirical relationship between meteorological variables and fluxes; this approach ignores the intricate physiological process feedbacks occurring within the SPAC.

As a component of Earth System Models (ESMs), Land Surface Models (LSMs) are extensively employed for quantifying land surface fluxes^24^. It is important for LSMs to consider the canopy physiological processes, root growth and water uptake, as well as soil water and heat transfer processes. Such systematic consideration of the SPAC processes is necessary, particularly in water-scarce regions, where the water-energy-carbon fluxes are regulated by the strong coupling between SM and land surface fluxes^25^. STEMMUS-SCOPE is a coupled process-based model that integrates canopy radiative transfer, the vertical profile of SM and soil temperature, and the dynamic root system. Validation conducted on a maize cropland in China and a grassland in the USA has demonstrated that STEMMUS-SCOPE effectively captures vegetation responses under water-stressed conditions and simulates the dynamics of soil heat and water movement, along with root growth and the corresponding root water uptake^25^. Additionally, STEMMUS-SCOPE serves as an effective forward simulator for simulating remote sensing signals, including reflectance, emittance, and solar-induced chlorophyll fluorescence^25–29^.

In this study, we hypothesize that the process-based model (e.g. STEMMUS-SCOPE) can generate reliable physically-consistent products of water-energy-carbon fluxes, along with corresponding above and below-ground hydrological, physiological, photosynthetic, and radiative variables. We conducted STEMMUS-SCOPE simulations with the PLUMBER2 dataset and generated an enhanced physically-consistent dataset that includes important soil states and water-energy-carbon fluxes. The product could assist other modelers or ecologists in comprehending global eco-hydrological processes.

## Methods

### Description of STEMMUS-SCOPE

STEMMUS-SCOPE is a 1-D model which couples a detailed canopy radiative transfer, energy balance, and photosynthesis model (SCOPE: Soil Canopy Observation, Photochemistry and Energy fluxes)^26^ with a two-phase vadose zone mass and heat transfer model (STEMMUS: Simultaneous Transfer of Energy, Mass and Momentum in Unsaturated Soil)^30^. The SCOPE model is a 1-D canopy model that simulates radiative transfer, photosynthesis, and fluorescence emission. It requires top-of-canopy incident radiation as input, typically obtained from atmospheric radiative transfer models like MODTRAN^26^. While SCOPE effectively represents energy exchange and carbon assimilation in the canopy, it lacks a soil module and does not represent for soil water dynamics, root water uptake, or root growth. Consequently, it relies on observed soil moisture data to simulate water stress, which limits its applicability in data-scarce environments.

To address these limitations, the SCOPE model was coupled with STEMMUS, a vadose zone model that simulates the coupled transport of energy, mass, and momentum in unsaturated soil. To address these limitations, the SCOPE model was coupled with STEMMUS—a vadose zone model that simulates the coupled transport of energy, mass, and momentum in unsaturated soils. STEMMUS integrates soil water and heat transport, providing dynamic soil moisture inputs to SCOPE and eliminating the need for external observations. Additionally, evapotranspiration and photosynthesis simulated by SCOPE drive root water uptake and growth in STEMMUS, forming a feedback loop that enhances simulation of soil–plant–atmosphere interactions. This coupling improves the accuracy of water stress simulations and ensures conservation of energy, mass, and momentum within the soil–plant–atmosphere continuum (SPAC). Therefore, STEMMUS-SCOPE simulates the transfer of optical, thermal, and fluorescent radiation, as well as water and carbon fluxes within the SPAC. It enables the generation of consistent ecohydrological datasets across diverse vegetation types without the need for parameter tuning^25,31–33^. The calculation of water stress factor, governing equations of the soil processes, equations of root growth and its water uptake can be found in Supplementary Texts S1 to S4.

### Datasets used in this study

#### Meteorological and biological forcing

As listed in Table 1, the meteorological forcing of this study is from the PLUMBER2 dataset. PLUMBER2 is the second phase of the Protocol for the Analysis of Land Surface Models (PALS) Land Surface Model Benchmarking Evaluation Project. PLUMBER2 conducted a multi-model (more than 20 land surface or biosphere models) intercomparison. For driving land surface models, fully gap-filled meteorological data of the 170 sites are provided after quality control. Additional meta-data, such as site descriptions, reference and canopy heights, plant functional types, and remotely-sensed leaf area index (LAI, the MODIS product MCD15A2H at 500 m spatial and 8-daily temporal resolution) are also provided^23^. The distribution of the 170 sites is shown in Fig. 2a and the detailed descriptions of each site can be found in Supplementary Table 2.

**Table 1:** The forcing variables, model parameters, and output variables used in this study.

	Variable	Description	Source	Unit
Forcing	Rin	Downward shortwave radiation	PLUMBER2	W m^−2^
	Rli	Downward longwave radiation	PLUMBER2	W m^−2^
	Ta	Air temperature	PLUMBER2	
	P	Air pressure	PLUMBER2	
	ea	Air vapor pressure	PLUMBER2	hPa
	Ws	Wind speed	PLUMBER2	m s^−1^
	Pre	Precipitaition	PLUMBER2	cm s^−1^
	LAI	Leaf area index	PLUMBER2	
	SMinit	Initial soil moisture	ERA5-Land	
	STinit	Initial soil temperature	ERA5-Land	
Parameters	Soil properties	Soil texture (sand, silt and clay contents), bulk density, and soil organic carbon content	GSDE	
	Soil hydraulic	Soil hydraulic parameters which were needed by the Van Genuchten (VG) model	CM_SoilHydraulic_1km	
	*F*_*max*_	Maximum saturated fractional area	SIMTOP	%
	Photo_Path	Photochemical pathway: C3 or C4	Prescribed	
	Slti	Slope of cold temperature decline (C4 only)	Prescribed	
	Shti	Slope of high-temperature decline in photosynthesis	Prescribed	
	Thl	Temperature below which C4 photosynthesis is lower than half that predicted by Q10	Prescribed	K
	Thh	Temperature above which photosynthesis is lower than half that predicted by Q10	Prescribed	K
	Trdm	Temperature at which respiration is lower than half that predicted by Q10	Prescribed	K
	Vcmax	Maximum carboxylation rate at 25 ^o^C	Prescribed	µmol m^−2^ s^−1^
	Cab	Chlorophyll content	Prescribed	ug cm^−2^
	m	Slope of Ball-Berry equation	Prescribed	
	BallBerry0	Intercept of Ball-Berry equation	Prescribed	
	Rdparam	Leaf respiration parameter	Prescribed	
	LIDFa	Parameter a of the leaf inclination distribution function	Prescribed	
	LIDFb	Parameter b of the leaf inclination distribution function	Prescribed	
	Leafwidth	Leaf width	Prescribed	m
	$\beta$	Root distribution parameter	Prescribed	
Output	Rntot	Net radiation		W m^−2^
	Rnc	Net radiation of canopy		W m^−2^
	Rns	Net radiation of soil		W m^−2^
	LEtot	Latent heat flux		W m^−2^
	LEc	Latent heat flux of canopy		W m^−2^
	LEs	Latent heat flux of soil		W m^−2^
	Htot	Sensible heat flux		W m^−2^
	Hc	Sensible heat flux of canopy		W m^−2^
	Hs	Sensible heat flux of soil		W m^−2^
	G	Ground heat flux		W m^−2^
	GPP	Gross primary production		kg C m^−2^ s^−1^
	NEE	Net ecosystem exchange		kg C m^−2^ s^−1^
	PAR	Photosynthetically active radiation		µmol m^−2^ s^−1^
	aPAR	Absorbed photosynthetically active radiation		µmol m^−2^ s^−1^
	SM	Soil moisture		m^3^ m^-3^
	ST	Soil temperature		^o^ C
	SIF	Solar-induced chlorophyll fluorescence		mW m^−2^ nm^−1^ sr^−1^
	Ref	Reflectance		
	ET	Evapotranspiration		cm s^−1^
	T	Plant transpiration		cm s^−1^
	E	Soil evaporation		cm s^−1^

Output Variables naming conventions follow the ALMA^85^ format where available.

#### Global soil texture and hydraulic parameters

Soil texture and hydraulic parameters are essential for LSMs and are equally crucial for STEMMUS-SCOPE. CM_SoilHydraulic_1km was used to provide the soil hydraulic parameters which were needed by the Van Genuchten (VG) model for describing the relationship between soil water content and soil water potential in this study. SoilGrids maps the spatial distribution of soil properties across the globe with state-of-the-art machine learning methods^34^. CM_SoilHydraulic_1km used the SoilGrids soil texture information as inputs to Pedotransfer Function, which then calculate soil hydraulic parameters for VG models^34^. For the vertical profile of soil texture (including sand, silt and clay contents), bulk density, and soil organic carbon content at 170 sites, the Global Soil Dataset for use in Earth System Models (GSDE) was used in this study^35^.

#### Consideration of surface runoff

In this study, SIMTOP, which is a simplified runoff parameterization of TOPMODEL, was used to calculate the surface runoff. It is consistent with the parameterization of runoff in the Common Land Model (CLM)^36^. This consideration prevents the soil from being saturated at the sites that have a large amount of precipitation. The maximum saturated fractional area (*F*_*max*_, %) is the key parameter in SIMTOP. Therefore, the global *F*_*max*_ dataset, which is also used by CLM, is adopted by the STEMMUS-SCOPE^37^.

#### Initial conditions

The simulations are very sensitive to the initial conditions, especially for the sites with short time series. To avoid the uncertainties introduced by the initial condition, the SM and soil temperature (ST) of the ERA5-Land dataset^38^ were used as the initial values at the start time of the simulation. The SM from ERA5-Land should first be constrained using the saturated and residual soil water content from SoilGrids. This constrained soil moisture can then be used as the initial value for the simulation. PyStemmusScope, which is the pre-processing module developed for preparing the input of STEMMUS-SCOPE, extracts the values from ERA5-land based on the location information and start time of each site’s timeseries.

#### Model validation

The PLUMBER2 dataset provides *in-situ* observed energy, water, and carbon fluxes, which consists of a time series with a duration that is consistent with the forcing data, for evaluating the simulations of STEMMUS-SCOPE. However, due to the missing SM and ST data in PLUMBER2, we used FLUXNET2015^21^ to validate the simulation of SM and ST of STEMMUS-SCOPE. It should be noted that there are 106 of the 170 sites that have SM measurements and 117 of the 170 sites that have ST measurements. In addition, the time series of *in-situ* SM and ST in the FLUXNET2015 are not complete. Therefore, we only compared simulated SM and ST when the observations are available. As PLUMBER2 lacks site-based measurements of Solar-Induced chlorophyll Fluorescence (SIF) and reflectance, and its simulations extend only through 2018, we used satellite-based products for model validation. A comparison of available satellite SIF datasets identified the Orbiting Carbon Observatory-2 (OCO-2) data as the most suitable for validating simulated SIF. The selection of OCO-2 was based on two main criteria: (1) Temporal coverage—available since 2014, which overlaps with our simulation period at several sites; and (2) Spatial and temporal resolution—OCO-2 offers a suitable resolution (0.05° and 16-day) for validating model outputs. Given these advantages, OCO-2 data was used for validating simulated SIF. For reflectance validation, we used the MODIS MOD09Q1 Version 6 product, focusing on two bands (Band 1: 620–670 nm and Band 2: 841–876 nm) due to its high spatial resolution (250 m)^39^. The detailed information about the datasets for validating simulations is listed in Supplementary Table 3.

#### Other parameters

To conduct the simulation, canopy physiological parameters and root growth parameters should also be defined. According to the original SCOPE model, the canopy parameters such as maximum carboxylation capacity (Vcmax), Chlorophyll content (Cab), Ball-Berry stomatal conductance parameters (BallBerry0 and m), and dark respiration rate (Rd), were set with a lookup table which is hard-coded within the STEMMUS-SCOPE model. The plant-dependent root distribution parameter ($\beta$) was adopted to simulate the vertical distribution of the root system^40^. The specific values of these parameters are shown in Supplementary Table 4.

### Experiment design

Multiple processing steps were undertaken to derive the final eco-hydrological dataset. First, the meteorological and LAI data (the MODIS LAI was used) from PLUMBER2 were used as the forcing input for the SCOPE model, while the initial SM and ST profile were retrieved from the ERA5-Land dataset and used as the initial condition for the STEMMUS model. Additionally, soil properties and runoff parameters were obtained from global datasets. Some plant feature parameters were pre-set and determined based on different PFTs using the lookup table (detailed parameter values are provided in Supplementary Table 4).

**Fig. 1:**
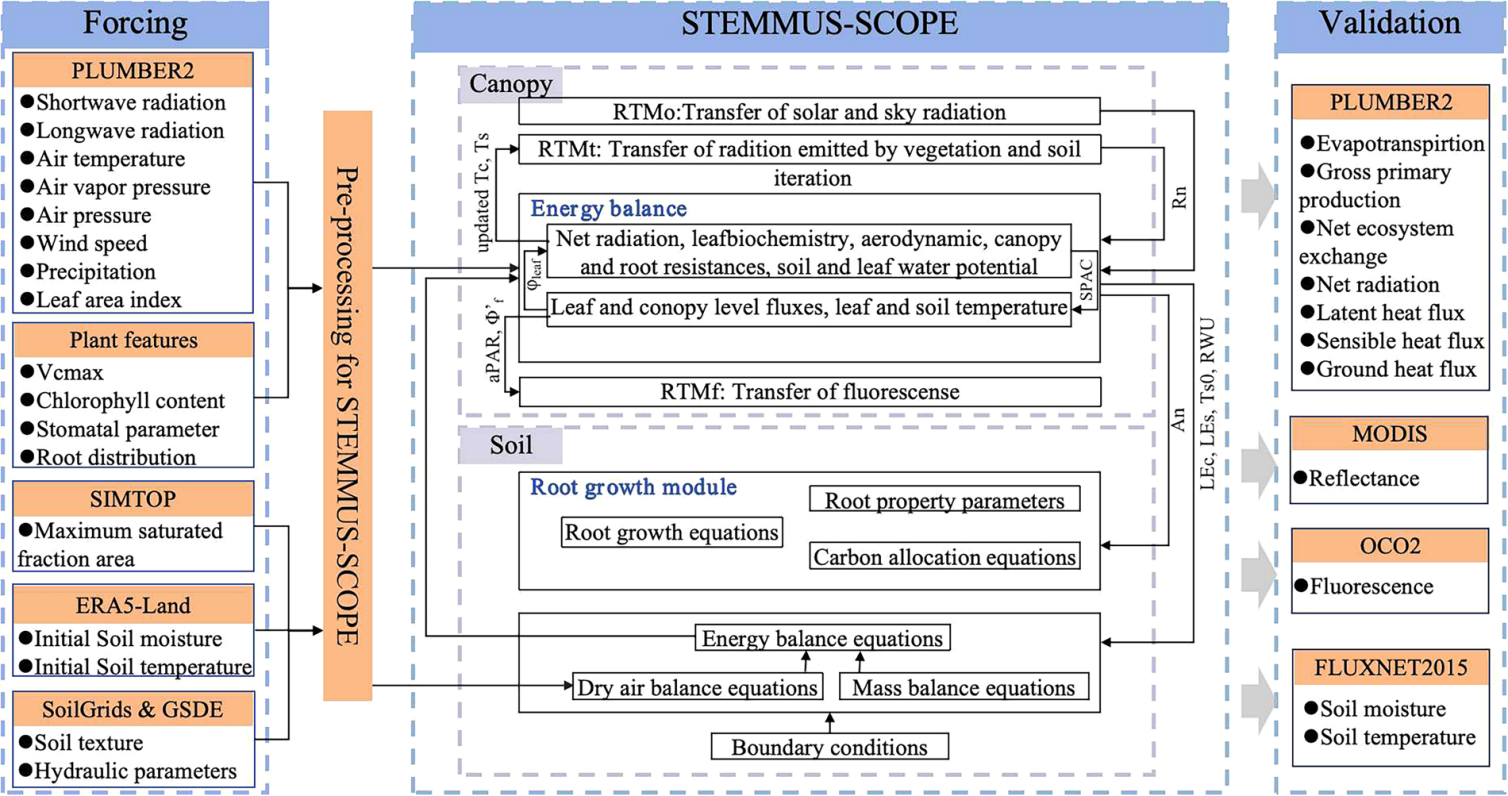
Schematic diagram of the overall workflow of this study.

During the data preparation phase, a pre-processing script (PyStemmusScope) was used to extract and standardize variables from different datasets, storing them in a unified folder. The STEMMUS-SCOPE model then read these input data for simulations. After that, we initially ran the model at three test sites (AU-Tum, US-Ha1, and FI-Hyy) to test STEMMUS-SCOPE at different forest types. Supplementary Fig. 1 shows that STEMMUS-SCOPE performed well at forest sites (3 sites are evaluated at the website: https://modelevaluation.org).Then, a one-year simulation for 170 sites was conducted to test the model setup. After all the settings were determined, the whole simulation of all 170 sites was conducted. Since the whole simulation required excessive computing power, it was run at the Dutch National Supercomputer ‘Snellius’ (https://www.surf.nl/en/dutch-national-supercomputer-snellius). After completing simulations for the 170 sites, the modeled energy, water, and carbon fluxes were evaluated against observations provided by PLUMBER2, while the simulated soil temperature and moisture were validated against observations from selected FLUXNET2015 sites. The simulated SIF was compared with OCO2 satellite observations, and the simulated reflectance was validated against MODIS satellite observations. A detailed flowchart of the simulation is described in Fig. 1, and all input, output, and model parameters used by STEMMUS-SCOPE are summarized in Table 1.

## Data Records

The derived dataset (Version v1.0.3) can be acquired from 10.5281/zenodo.7737321^41^. It contains half-hourly energy and carbon fluxes and soil moisture and soil temperature data of 170 sites. These data are stored in NetCDF format with one file per site. A detailed descriptions of the variables is listed in Table 1.

### Statistics

To evaluate the simulations, the Kling–Gupta efficiency (KGE), R^2^, RMSE, rRMSE, and rSD were used. The calculation of KGE is as follows^42^:

1 $${KGE}=1-\sqrt{{\left(r-1\right)}^{2}+{(\alpha -1)}^{2}+{(\beta -1)}^{2}}$$

Where $r$ is the Pearson correlation coefficient; $\alpha ={\sigma }_{s}/{\sigma }_{o}$ is the variability of simulation errors; $\beta =\,{\mu }_{s}/{\mu }_{o}$ is the bias. ${\sigma }_{s}$ and ${\sigma }_{o}$ are the standard deviations of the simulation and observation, respectively. ${\mu }_{s}$ and ${\mu }_{o}$ are the mean of the simulation and observation, respectively.

Based on the calculated KGE, we assessed the simulation for each site. The datasets were classified into four levels: ‘Excellent’, ‘Good’, ‘Average’, and ‘Poor’. These classifications can assist users in selecting sites as needed for conducting analyses. Detailed criteria are provided in Supplementary Table 5.

## Technical Validation

### Evaluation against *in-situ* observation

**Fig. 2:**
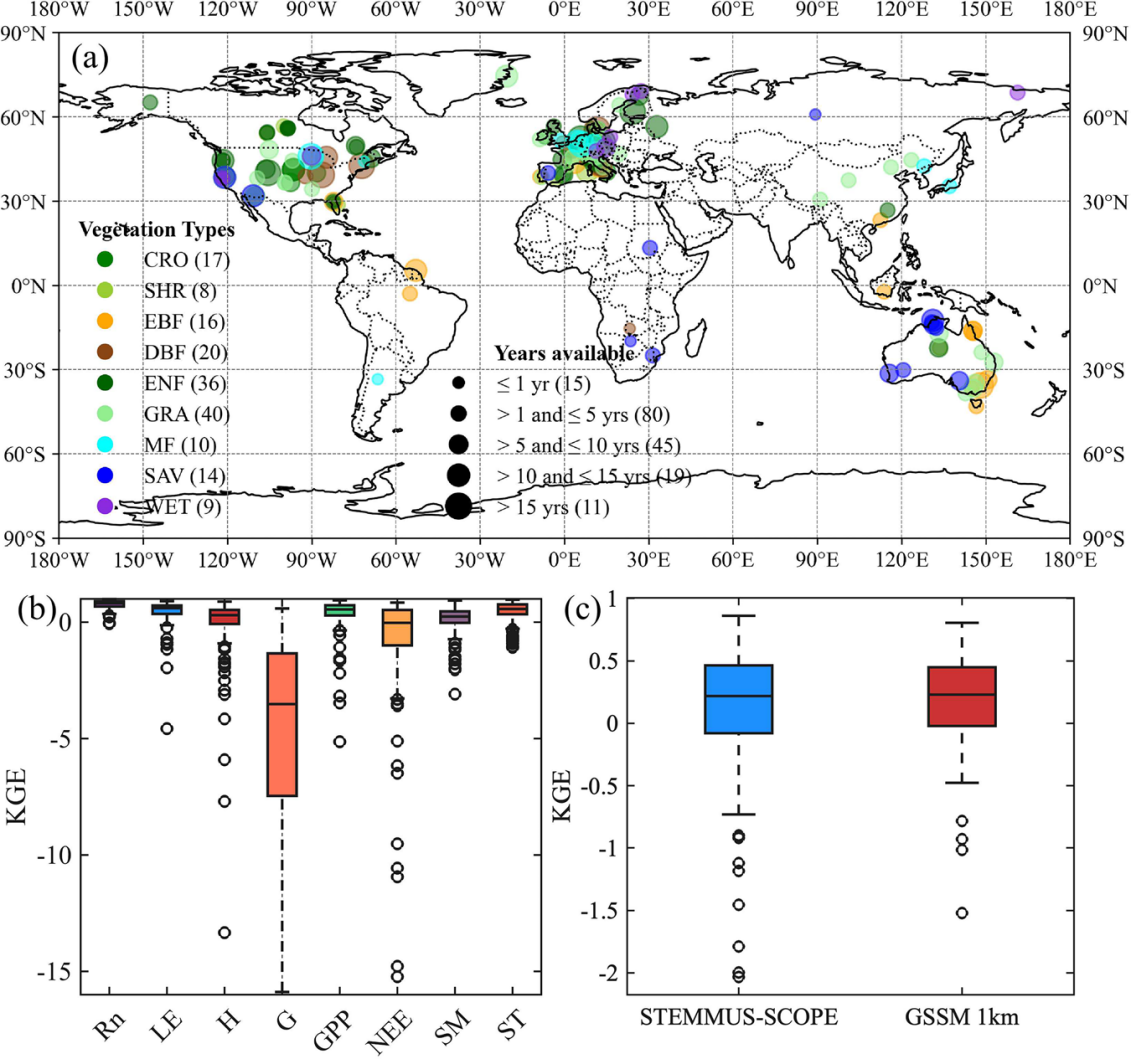
(**a**) Global distribution of PLUMBER2 sites; (**b**) Performance (Kling–Gupta efficiency, KGE) of STEMMUS-SCOPE (box plots) for the validation set of observations; (**c**) Performance (KGE) of STEMMUS-SCOPE and GSSM 1 km (box plots) for the validation set of observations. The box plots show (from top to bottom) the maximum, 75th percentile, median, 25th percentile, and minimum. The whiskers extend to the most extreme data points not considered outliers, and the outliers are plotted individually using the ‘○’marker symbol.

**Fig. 3:**
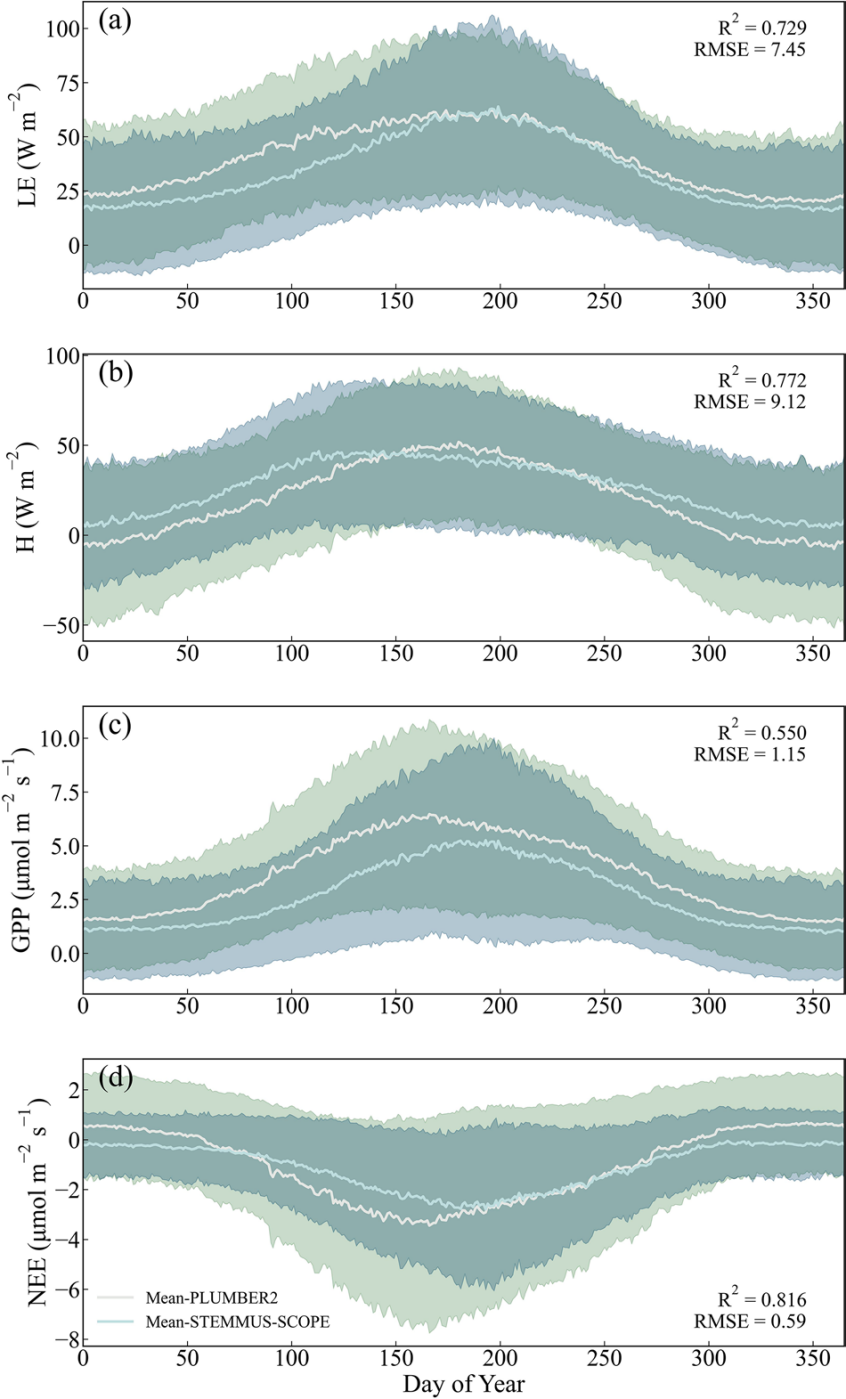
Time series of modeled and observed daily LE, H, GPP, and NEE for 170 sites.

Tower-based observations from PLUMBER2 were utilized to validate the simulated energy and carbon fluxes. The evaluations are depicted as box plots with outliers explicitly displayed (Fig. 2b). During assessment of the entire validation set, STEMMUS-SCOPE exhibited the best performance in net radiation (Rn). The Kling–Gupta efficiency (KGE) ranged from 0.37 to 0.99, with the highest median value being 0.80 (median R², RMSE, rRMSE, and rSD values are 0.97, 38.6 W m^−2^, 4.09%, and 0.07, respectively). However, the performance of STEMMUS-SCOPE in the simulation of G exhibited considerable uncertainty. The KGE ranged from −15.88 to 0.58, with a median value of -3.52 (median R², RMSE, rRMSE, and rSD values are 0.22, 32.8 W m^−2^, 19.12%, and 1.18, respectively). The simulation of LE and H were comparable. For LE, the KGE ranged from -0.32 to 0.93, with a median value of 0.60 (median R², RMSE, rRMSE, and rSD values are 0.63, 46.2 W m^−2^, 7.29%, and 0.17, respectively). As for H, the KGE ranged from −1.18 to 0.90, with a median value of 0.30 (median R², RMSE, rRMSE, and rSD values are 0.68, 58.1 W m^−2^, 8.49%, and 0.26, respectively). The simulation of GPP is slightly more accurate than that of NEE. For GPP, the KGE ranged from -0.35 to 0.93 with a median value of 0.55 (median R², RMSE, rRMSE, and rSD values are 0.64, 3.79 *µ*mol m^−2^ s^−1^, 6.15%, and 0.18, respectively). For NEE, the KGE ranged from −12.76 to 0.93 with a median value of −0.03 (median of R², RMSE, rRMSE, and rSD values are 0.54, 4.00 *µ*mol m^−2^ s^−1^, 7.9%, and 0.20, respectively). The specific values are provided in Supplementary Table 6, while the detailed statistical values of each site are illustrated in Supplementary Fig. 2. We also compared the time series of observed and modelled daily LE, H, GPP, and NEE and found that the model captured annual variation of energy and carbon fluxes well (Fig. 3). Additionally, the time series of observed and modelled daily LE, H, GPP, and NEE of different vegetation type were presented in Supplementary Figs. 4 to 7. The 9 vegetation types including: SHR (Open/Closed Shrublands), CRO (Croplands), DBF (Deciduous Broadleaf Forests), EBF (Evergreen Broadleaf Forests), ENF (Evergreen Needleleaf Forests), GRA (Grasslands), MF (Mixed Forests), WET (Wetlands), and SAV (Woody Savannas). We found that STEMMUS-SCOPE slightly underestimated LE from January to June for most vegetation types except EBF. H is overestimated at DBF, ENF, and MF, and it is underestimated at WET. GPP is consistently underestimated across different vegetation types, and NEE shows significant deviations in SAV.

Next, we estimated the data quality of energy and carbon fluxes at each site based on the statistical indicators. As the simulations of Rn were excellent, but those of G were unsatisfactory for most sites, these two variables were excluded in the quality classification. It should be noted that some PLUMBER2 sites lack observed G data. Furthermore, due to the absence of observation depths for G and corresponding soil moisture data at these depths, even when G observations are available, conversion to surface G is still challenging. This limitation prevents a fair comparison between observed and model-simulated G^43^. GPP was excluded because NEE can be directly observed, whereas GPP was derived from observed NEE. Thus, the simulations were primarily evaluated based on the KGE of LE, H, and NEE. As shown in Supplementary Table 7, out of the 170 sites evaluated, 32 sites were rated as ‘Excellent’, 64 sites as ‘Good’, 59 sites as ‘Average’, and 15 sites as ‘Poor’ for the energy and carbon fluxes.

As critical indicators of soil dynamics, the accuracy of SM and ST simulations directly reflects the reliability of other simulated fluxes. However, due to the absence of SM and ST observations in PLUMBER2, *in-situ* measurements from FLUXNET2015 were utilized to evaluate the accuracy and reliability of the simulations. A total of 123 sites with measurements of SM or ST were selected, as indicated in Supplementary Table 3. We compared the hourly or half-hourly *in situ* observations with the dataset derived from the model. For validation reliability, sites with explicit measurement depths and relatively higher-quality data were selected. As illustrated in Fig. 2b, the KGE values for SM across 96 sites varied from −0.73 to 0.93 with a median value of 0.24 (The median values of R^2^, RMSE, rRMSE, and rSD are 0.46, 8.84% m^3^ m^−3^, 29.11%, and 0.34, respectively). For ST, the KGE ranged from −0.31 to 0.96 with a median value of 0.56 (The median values of R², RMSE, rRMSE, and rSD are 0.88, 3.54 °C, 13.8%, and 0.39, respectively) (Supplementary Table 6). This indicates the strong capability of STEMMUS-SCOPE in tracking soil thermal and water dynamics. To further demonstrate the effectiveness of the SM simulated by STEMMUS-SCOPE, we compared it with an advanced, high-resolution global surface SM dataset (GSSM 1 km)^44^. Figure 2c shows that the KGE values of STEMMUS-SCOPE ranged from −0.73 to 0.86 with a median value of 0.22, which is comparable with the median value of GSSM 1 km (0.23). These indicate that STEMMUS-SCOPE is capable of capturing both surface and root zone SM dynamics. We further assessed the accuracy of SM simulation for each site.As shown in Supplementary Table 7, 38 sites were rated as ‘Excellent’, 33 sites as ‘Good’, 22 sites as ‘Average’, and 13 sites as ‘Poor’ based on the KGE value of SM at each site.

### Description of the results of different vegetation types (from selected sites)

**Fig. 4:**
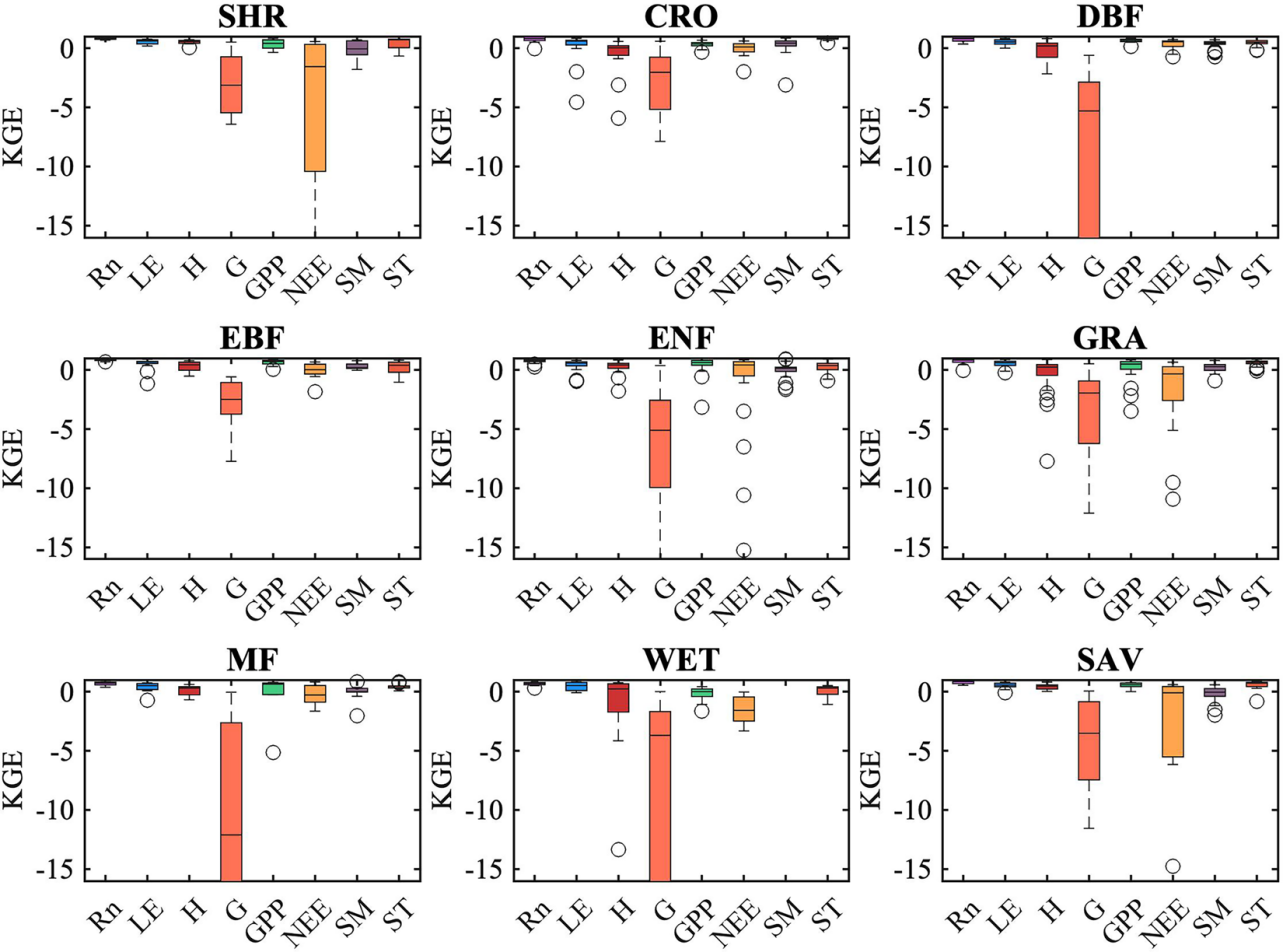
Box plots showing the Kling–Gupta efficiency (KGE) of STEMMUS-SCOPE against *in-situ* measurements at 9 vegetation types. On each box, the central mark indicates the median, and the bottom and top edges of the box indicate the 25th (q25) and 75th (q75) percentiles, respectively. (Note: SHR is (Open/Closed) Shrublands, CRO is Croplands, DBF is Deciduous Broadleaf Forests, EBF is Evergreen Broadleaf Forests, ENF is Evergreen Needleleaf Forests, GRA is Grasslands, MF is Mix Forests, WET is Wetland, SAV is (Woody) Savannas).

The simulations for various vegetation types were analyzed. Figure 4 demonstrates that the model accurately simulated Rn and ST for all vegetation types, consistent with the findings in Fig. 2b. Nonetheless, variations exist among the 9 vegetation types (SHR including Closed Shrublands and Open Shrublands; SAV including Savanna and Woody Savanna) regarding other output variables. The model exhibited better performance in simulating LE for forest sites, such as EBF, DBF, ENF, and MF, characterized by relatively high LAI. The model performed well in simulating H for most vegetation types except WET. The model only achieved relatively good performance in simulating G at SAV sites. Regarding GPP and NEE, the outcomes resembled those of LE due to the strong coupling between transpiration and carbon assimilation. The typical sites for each vegetation type are presented in Supplementary Figs. 8 to 16. Finally, simulations of SM were notably superior in EBF and DBF compared to other vegetation types, and the simulations of SM for two typical sites are shown in Supplementary Figs. 17 and 18.

### Simulation of other variables

**Fig. 5:**
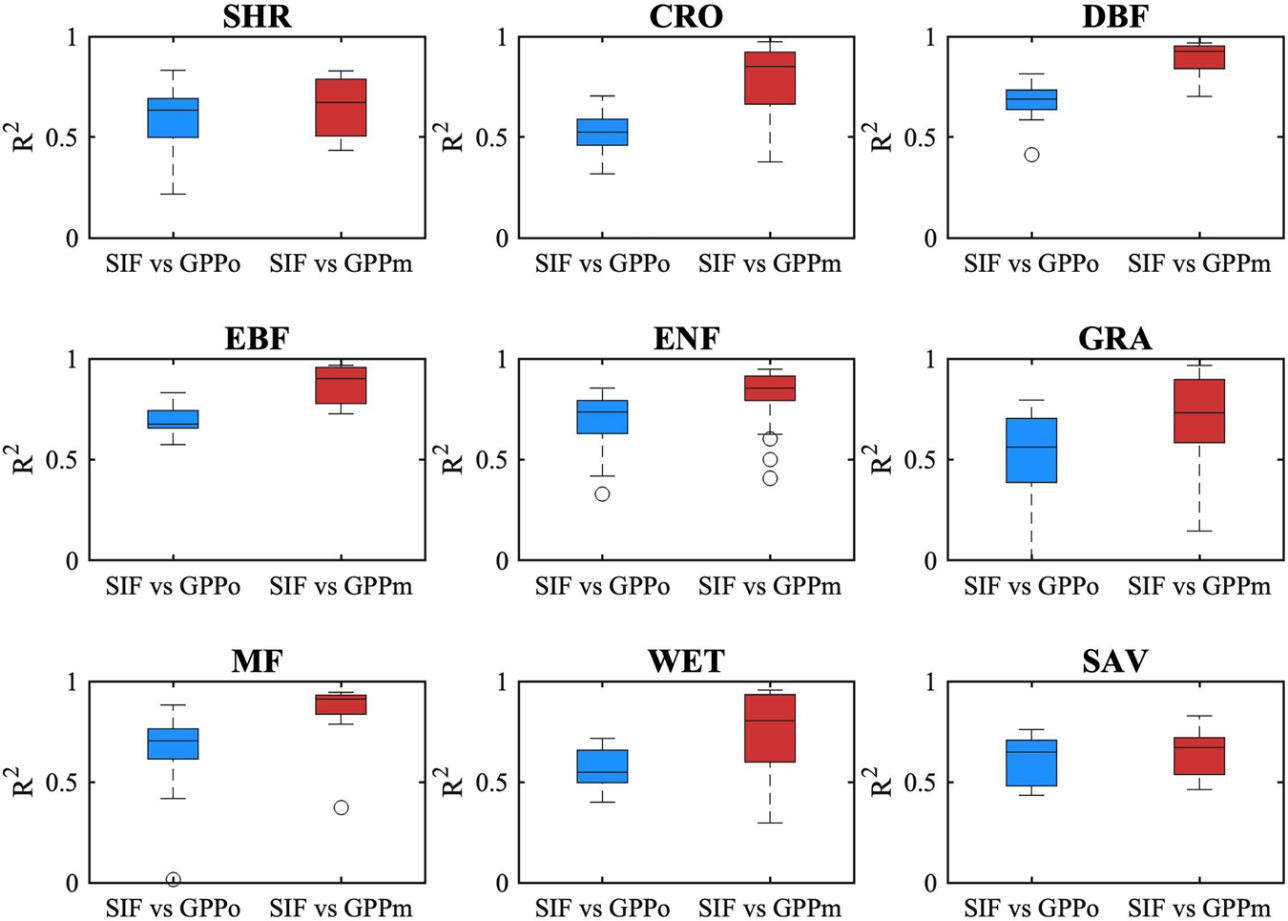
Box plots showing the coefficient of determination (R^2^) of modelled SIF against observed (GPPo) and modeled GPP (GPPm). The symbols are the same as in Fig. 3.

The model also simulated SIF, reflectance spectrum, and canopy temperature, as well as their profile within canopy layers, although validating these with observations is currently challenging. Due to the time frame of this dataset spanning from 1992 to 2018, it was not feasible to validate the simulated SIF with tower-based SIF. Therefore, we compared our simulated SIF with satellite observations from OCO-2. By aligning the time periods of OCO-2 observations with our model simulations, we validated the simulated SIF at 58 stations, with most validation data concentrated between September and December 2014. For several Australian sites, validation extended through the end of 2018. To ensure compatibility with OCO-2 measurements, we computed a weighted average of the simulated SIF at 757 nm and 771 nm using the formula: (SIF_757_ + 1.5 × SIF_771_)/2^45^. The resulting SIF values were then smoothed using a 16-day moving average to capture seasonal patterns. As illustrated in Fig. 7a, the smoothed simulated SIF closely matches OCO-2 observations, with a median R² of 0.650 and a median RMSE of 0.106 mW m^−2^ nm^−1^ sr^−1^. Furthermore, the model successfully captures the seasonal dynamics of SIF, as shown in Fig. 7d. To further test the reliability of simulated SIF, we also examined the correlation between modeled SIF and GPP (both observed and modeled), revealing a strong proportionality between modeled SIF and observed or modeled GPP (Fig. 5).

To validate the reflectance simulated by STEMMUS-SCOPE against MODIS observations, the MODIS Spectral Response Function (SRF) was applied to derive corresponding spectral bands from the model’s full-spectrum output. The simulated reflectance values were then averaged between 9:30 and 11:30 a.m.—the typical MODIS overpass time—across 8-day intervals to ensure temporal consistency. As shown in Fig. 7b and c, the agreement between simulated and observed MODIS reflectance is generally weak. For Reflectance Band 1, the median R^2^ is 0.19 with a median RMSE of 0.016, while for Band 2, the median R^2^ is 0.22 with a median RMSE of 0.010. Model performance also varies significantly across different sites. Where vegetation and soil primarily control surface reflectance, the simulated values closely align with MODIS observations (Fig. 7e). However, large discrepancies occur during periods of snow cover or extended rainfall, as expected, since the current model configuration does not account for the influence of snow, rain, or clouds on surface reflectance (Fig. 7f).

**Fig. 6:**
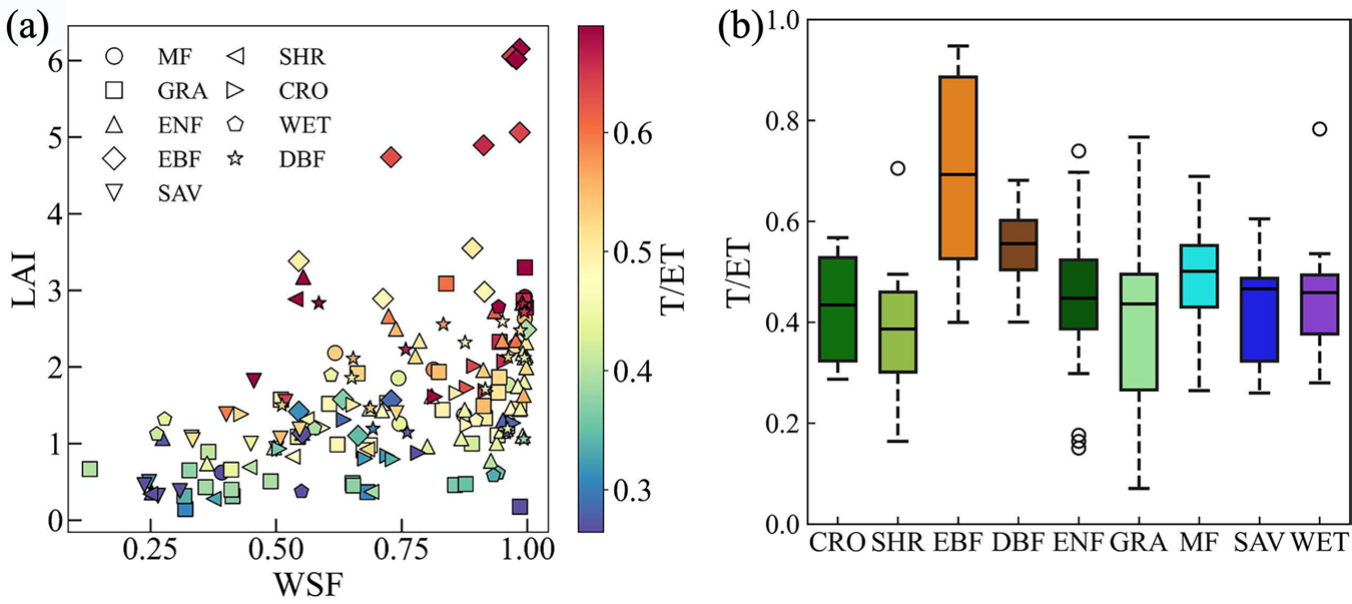
(**a**) Scatter plot of LAI and WSF with color-coded T/ET ratio for 170 sites with different vegetation types; (**b**) Boxplot of T/ET ratios for different vegetation types.

Additionally, the model simulated canopy (LEc) and soil latent heat flux (LEs), representing plant transpiration and soil evaporation, respectively. All variables of this generated physically consistent long-term dataset are listed in Table 1. To test the ability of STEMMUS-SCOPE in ET partitioning, we analyzed the simulated T/ET ratios under varying water statuses and LAI levels. As shown in Fig. 6a, the T/ET ratios increase with LAI and soil moisture. The median T/ET ratio for EBF is the highest at 0.69, and the GF-Guy site has the highest value at 0.95. The median T/ET ratio for DBF is 0.56, which is relatively high. SHR exhibits the lowest median T/ET ratio at 0.39. However, the minimum T/ET ratio is observed in the GRA (DK-Lva) at 0.07. The median T/ET ratios for other vegetation types range between 0.43 and 0.50 (Fig. 6b).

### Uncertainty analysis

The novel LSMs include processes representing energy, mass, and momentum transfers in the SPAC system on a wide range of spatiotemporal scales. This inevitably results in increased model complexity to account for the interactions between the atmosphere and the land surface. The evaluations of simulated land surface fluxes, SM, and ST remain highly uncertain^46–49^.

The conceptual illustration of uncertainty sources in the simulations is shown in Supplementary Fig. 19. The uncertainty of the datasets produced by STEMMUS-SCOPE could be classified into four types:

1. Uncertainties in forcing data: the gap-filled meteorological data and the vegetation and soil information for running STEMMUS-SCOPE (e.g., radiation, precipitation, air temperature, wind speed, humidity, LAI, initial conditions of SM and ST);
2. Uncertainties of the parameters: the fixed default parameters used to simulate the eco-hydrological processes (e.g., Vcmax, saturated SM, saturated conductivity (Ks));
3. Uncertainties in the model structure: inaccurate representations of bio-physiological processes (e.g., ignored soil freezing-thawing processes, the 1-D nature of the model);
4. Uncertainties in the measurements used for model validation.

#### The uncertainties in forcing data

The proposed dataset is derived based on forcing data provided by PLUMBER2. The uncertainties that exist in the forcing data (i.e. LAI and precipitation), due to the gap-filling algorithm, will be propagated to the outputs. We next discuss uncertainties related to LAI, precipitation, land cover and vegetation properties among others.

As Supplementary Fig. 20 shows, the simulated GPP can be considerably underestimated due to the low LAI input^50^. To further test the uncertainty induced by LAI, we doubled the value of LAI while keeping other driving data unchanged. The results showed that the simulated values of GPP and NEE increased significantly (KGE increased from −0.24 to 0.01 for GPP, and from −1.02 to −0.58 for NEE respectively; Supplementary Figs. 20 to 22). Although there are two LAI time series in PLUMBER2 that can be used to drive LSMs, it should be noted that much uncertainty still exists in the remote sensing LAI when it is used at site scale. Such large uncertainties in remote sensing LAI products include the errors in retrieving LAI from satellite images and the mismatching between the spatial-scale of the satellite data and the site-scale fluxes^51^. Numerous studies have shown that LAI is a key forcing data of LSMs and it directly influences the radiative transfer and photosynthesis^51,52^. Especially for the sparsely vegetated areas (e.g. arid and semi-arid areas), the uncertainties in LAI result in large deviations in the simulations of GPP and LE^53^. Additionally, the uncertainty of LAI can significantly affect the simulation of reflectance in the SCOPE model, thereby influencing radiative transfer as well as the energy and water-carbon balance of the canopy and soil. As such, precise input LAI would be greatly valuable for constraining models. Incorporating field-measured LAI into forthcoming flux tower databases would enable bias-correction and validation of remote sensing LAI, subsequently facilitating their utilization in model forcing or direct evaluation of simulated LAI by models.

As an important forcing variable, the quality of precipitation data determines the accuracy of simulated SM which is sensitive to precipitation, especially in water-scarce areas^54–56^. Generally, sensor malfunctions or blockages in the rain bucket can lead to missing precipitation data. Subsequent gap-filling of precipitation data by global reanalysis dataset also introduces uncertainty. As Supplementary Fig. 23 shows, towards the end of the time-series, the dynamics of observed SM are not consistent with the precipitation events of the ZM-Mon site.

Determination of the vegetation type remains challenging, especially for mixed vegetation. In this study, we found that the descriptions of vegetation types for some sites were inconsistent between the driving data files and the validation data files. This discrepancy may stem from the inability to clearly delineate vegetation types for some sites. Additionally, during long-term observation experiments, certain sites underwent land use changes, which altered their vegetation types. Vegetation type determines the canopy parameters including chlorophyll content (Cab), maximum carboxylation capacity (Vcmax), dark respiration (Rd), and photosynthetic pathway (Photo_Path). Especially for the rotating cropland, the different photosynthetic type introduces large uncertainty in GPP. As we know, the water use efficiency and light use efficiency of C4 plants are higher than that of C3 plants as they have a higher light saturation point. Except for some grassland and cropland sites explicitly specified as having vegetation such as maize, switchgrass, or other C4 plants, all other sites are assumed as C3 vegetation. Analysis of two typical rotation croplands (US-Ne2 and US-Ne3: maize/soybean rotation) revealed significant inter-annual variations in observed GPP. As Supplementary Figs. 24 and 25 show, the LAI is comparable for maize and soybean; however, the observed GPP of maize is significantly higher than that of soybean. Consequently, simulations of crop rotations still exhibit significant uncertainties. In addition, the root distribution is also linked with vegetation type, such that the vegetation type could affect the root zone SM. Moreover, it should be noted that the uncertainty of LAI is greater at sites with unclear vegetation types because of the mixture of tall and short vegetation.

Finally, the uncertainty of reference height (z) and canopy height (hc) introduces significant deviations in energy flux calculations. On the one hand, the reference height (z) and hc provided by PLUMBER2 are kept constant in the model and therefore cannot realistically represent the actual conditions, especially for croplands and seasonal grasslands. On the other hand, the theory of eddy covariance requires that meteorological variables should be observed at a reference height above the canopy^57^, and so *z* is expected to be higher than *hc*. However, due to the lack of clarity in various site descriptions, the *hc* of some sites (including AU-Wrr, AU-Rob, and CN-Din sites) are higher than *z* in PLUMBER2, which makes it difficult for the model to converge when iterating for energy balance and results in unreasonable sensible heat flux with incorrect *hc*. For example, the site description for AU-Wrr states that the forests attain mature heights over 55 m and the tallest trees can reach 90 m, while the instruments are mounted at 80 m. Therefore, in the PLUMBER2 dataset, the reference height is 80 m and the canopy height is 90 m for AU-Wrr. Although we have corrected this issue during the simulation, the inconsistency between the *hc* provided by PLUMBER2 and the site description or the actual condition still remains significantly uncertain. This finding indicated that a physical consistent model can also be used to test the physical consistency of site data.

#### Canopy physiological parameters and soil property and hydraulic parameters

Whether the canopy physiological, soil physical and hydraulic parameters are adequate determines directly the performance of the model. We hypothesized that the canopy physiological parameters are relatively stable for each vegetation type, but the ignored seasonal or annual variations in these parameters may contribute to the uncertainties of the simulations. To date, with many published global datasets, such uncertainties can be further assessed. For example, the chlorophyll content (Cab) and maximum carboxylation capacity (Vcmax) are set as constants for each vegetation type, although it has been reported that these parameters may have significant seasonal and annual variabilities^58–60^. In addition, the soil hydraulic parameters could introduce uncertainty in SM simulation. For example, the saturation and residual soil water content determine the maximum and minimum values of simulated SM and the saturated hydraulic conductivity and water retention parameters of the Van Genuchten (VG) model determine the dynamics of SM. The soil parameters in this study are extracted from global datasets. Therefore, the mismatching between the grid and site scales exists and may also induce uncertainties. Specific field and lab works can be carried out to investigate specific sites, and we encourage the inclusion of the soil textures and soil hydraulic parameters in the FLUXNET site data.

#### Missing processes in the model structure

**Fig. 7:**
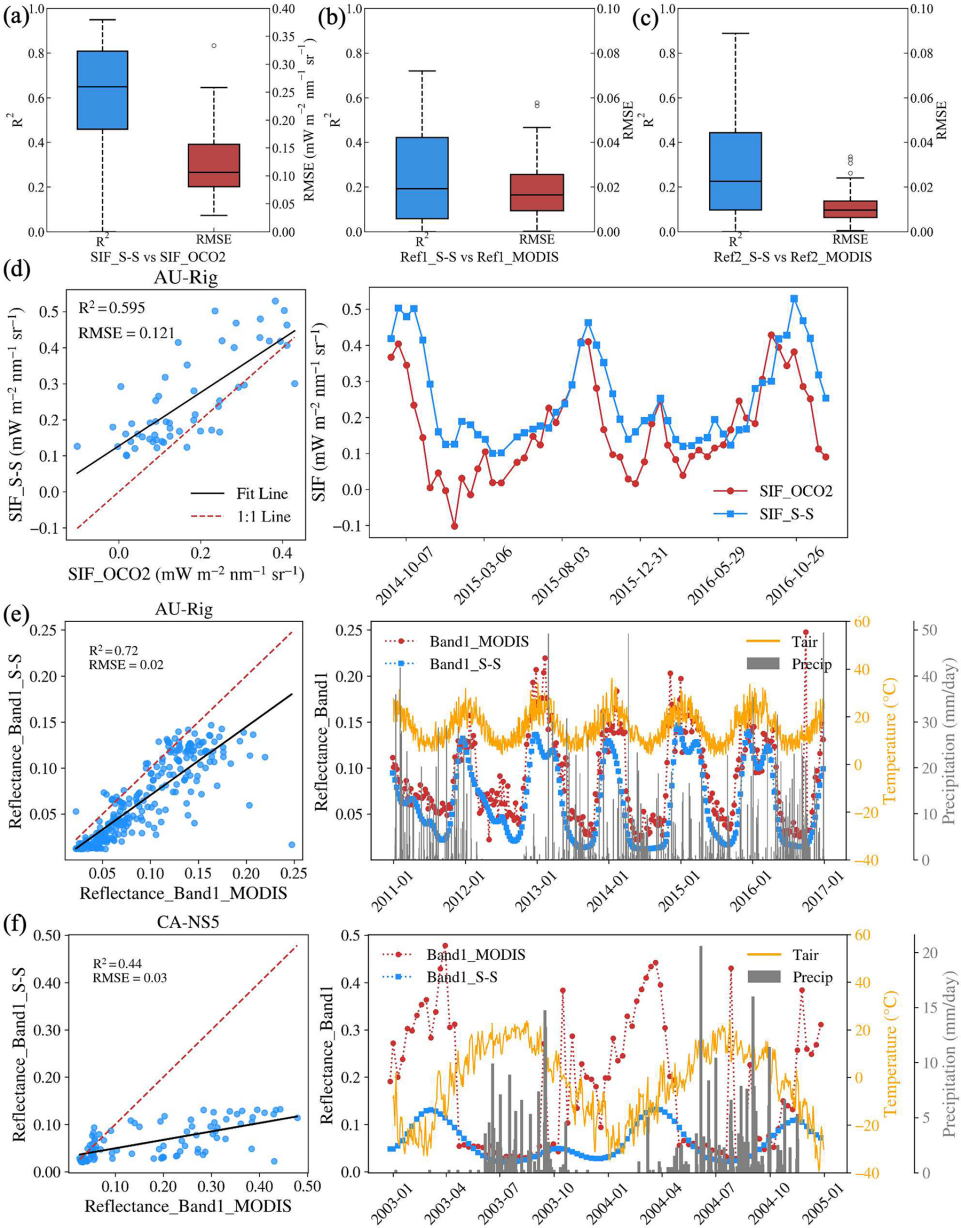
(**a**) Boxplot of R^2^ and RMSE for OCO-2 satellite SIF and modeled SIF (SIF-S-S). (**b**) Boxplot of R^2^ and RMSE for MODIS reflectance (Band 1) and modeled reflectance. (**c**) Boxplot of R^2^ and RMSE for MODIS reflectance (Band 2) and modeled reflectance. (**d**) Scatter plot and time series of modeled and satellite-observed SIF (OCO2) at AU-Rig site. (**e,f**) Scatter plots and time series of modeled and satellite-observed reflectance (MODIS) at AU-Rig and CA-NS5 site.

Although STEMMUS-SCOPE is a novel process-based LSM, it still does not contain all eco-hydrological processes. For example, processes related to freeze-thaw have been ignored which results in a large uncertainty of the SM simulation during frozen soil conditions (Supplementary Fig. 26). The user should pay attention when using the SM data at high latitude or high-altitude areas, though the vegetation is usually dormant during these periods. STEMMUS-SCOPE simulates the aggregate soil water content (sum of liquid water and frozen water), but most SM sensors only measure the liquid soil water content^61–63^. So, the model simulation in the freezing periods cannot be validated. In addition, the omission of freeze-thaw cycles and snow cover processes can introduce significant biases in reflectance simulations. For instance, at Canadian sites such as CA-NS5, the reflectance simulated by STEMMUS-SCOPE during winter is considerably lower than MODIS observations (Fig. 7(f)).

In addition, although the plant hydraulic module appears practical, it is relatively simple compared to those in other LSMs^24,64–68^, introducing a state-of-the-art explicit plant hydraulic scheme can further improve the performance of STEMMUS-SCOPE in arid areas. Coupling plant hydraulics in land surface models (LSMs) allows for the accurate determination of plant water potentials (root, stem, and leaf), better reflecting water stress. This approach captures the water stress responses of different plant species and improves the estimation of water, energy, and carbon fluxes^68–72^. The current version of STEMMUS-SCOPE only considers the water potential difference between the leaf and soil, while the water potential in the xylem and roots is not accounted for^25^. Introducing a state-of-the-art explicit plant hydraulic scheme could enhance the performance of STEMMUS-SCOPE, particularly in arid regions. The leaf water potential-based water stress factor (PHWSF) in Community Land Model Version 5.0 (CLM5) increases sensitivity to atmospheric drought compared to CLM4.5^69^. Coupling plant hydraulics with the ED2 model allows the model to capture diverse phenologies across different plant species^68^. By incorporating whole plant hydraulics with water storage, the Noah-MP-PHS model captures the hydraulic behaviors of isohydric and anisohydric plants during the dry-down period^70^. Furthermore, the simulation of ET and GPP by VIP-PHS is significantly improved by integrating plant hydraulics into the Vegetation Interface Process model (VIP)^72^. Recently, an advanced plant hydraulic model incorporating xylem vulnerability has been implemented in STEMMUS-SCOPE (STEMMUS-SCOPE-PHS)^71^. The leaf water potential-based water stress factor (PHWSF) replaces the original soil moisture-based stress factor, better representing the impact of water stress on plant growth. The PHWSF captures the diurnal dynamics of water stress, improving the simulation of LE, NEE, and GPP. Although STEMMUS-SCOPE-PHS has been published, the advanced plant hydraulics model introduces more parameters which limits its global-scale application. Therefore, STEMMUS-SCOPE-PHS requires further validation at the PLUMBER2 sites.

#### Uncertainty in measurements used for validation

Uncertainties exist in the observations. Although PLUMBER2 has conducted quality control and gap-filling, some fluxes still have outliers. For example, there are many negative values in GPP due to the GPP being partitioned by the night time-based approach^21,73^ (Supplementary Fig. 27). In addition, the ground heat flux is not corrected to that at the soil surface which is the main reason for the low correlation between simulated and observed G. Furthermore, the measurements of SM have a large number of outliers due to the sensor failure and the measured depths that are not explained at some sites (Supplementary Fig. 28). These make it very difficult to verify the simulation of SM profiles. Since no *in-situ* observations are available, the validation of SIF and reflectance relies on comparisons with satellite products. However, the spatial scale mismatch between satellite observations and site-level simulations introduces inherent uncertainty into the evaluation of model performance.

**Fig. 8:**
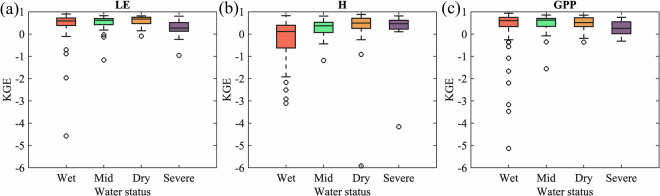
Box plots showing the Kling–Gupta efficiency (KGE) of modelled LE, H, and GPP against observed ones under different levels of water status. The symbols are the same as in Fig. 4.

To test the performance of STEMMUS-SCOPE at different levels of water status, we divide 170 sites into four groups based on their mean water stress factor (WSF, which is calculated by STEMMUS-SCOPE based on the vertical profile of root distribution and root zone soil moisture). The detailed criteria are shown in Supplementary Table 8. As shown in Fig. 8, the median KGE of LE and GPP slightly decreases with the increase of water stress, while the KGE of H increases with the increase of water stress. The reason is the strong controlling effect of radiation on LE and GPP in wet sites and while the impact of surface temperature becomes more important on H in dry sites (i.e. more radiation is partitioned to sensible heat resulting in higher surface temperature). These indicate that STEMMUS-SCOPE is capable in both wet and dry sites.

### Potential usage and the next step

The dataset could be used in several applications, including ecological and eco-hydrological studies, remote sensing studies, and contribute to the further development of process-based models. Furthermore, this dataset can be used to assess how changes in overall water availability across the globe influence the ability of terrestrial ecosystems to sequester carbon dioxide from the atmosphere^74^. The product of this study provides physically consistent fluxes data and multi-layer root zone SM and ST, based on STEMMUS-SCOPE simulations, and contributes to the understanding of terrestrial energy, water, and carbon cycles. Therefore, the SM can be used to quantify the relationship between soil water availability and eco-hydrological processes. Lastly, the dataset can be used for benchmarking various types of models, such as hydrological models, LSMs, and ESMs.

In our future work, to minimize uncertainty in the product, especially for the mixed vegetation, a vegetation growth module combined with data assimilation will be introduced into STEMMUS-SCOPE. The LAI in the current version is simply linearly interpolated from available remote sensing observations, while the actual LAI may have a higher temporal dynamics, especially for croplands. Many studies reported that combining vegetation growth model with remote sensing LAI using data assimilation methods improved the simulation of LAI as well as other vegetation growth processes^75^. To improve the quality of simulations for arid areas, further improvement in the plant hydraulics module is also needed. Upon the improvement in simulating plant hydraulics, the simulated plant transpiration and soil evaporation may be validated with SAPFLUXNET^76^. After such improvements, a global simulation can be conducted using re-analysis products as the forcing data and using International Soil Moisture Network (ISMN)^77^ and different global products of GPP^78–80^ and ET^81,82^ to validate the simulation. In addition, global satellite SM products, such as those from AMSR-E, SMOS-IC, and SMAP^83,84^, could also be used to validate the simulated global SM by STEMMUS-SCOPE.

## Usage Notes

This study achieved the goal of creating a physically consistent long-term energy, water, and carbon fluxes as well as multilayer SM profile products (of 0–500 cm depth) that displayed realistic temporal evolutions without gaps. The datasets generated by STEMMUS-SCOPE exhibit excellent agreement with *in-situ* measurements. Consequently, we advocate for the utilization of these datasets, such as identifying and attributing historical fluctuations in land-surface fluxes and soil moisture, including their associated extreme events, as well as benchmarking different types of remote sensing or process-based models. Future developments may aim at global gridded datasets based on re-analysis of meteorological data and consider more detailed physiological processes (e.g., vegetation growth, groundwater, freeze-thaw process, and plant hydraulics). We hope that the published dataset will aid the further development of ESMs for better-representing soil heat and water movements, as well as land-atmosphere exchanges involving energy, water, and carbon.

## Supplementary information

Supplement of A Physically Consistent Dataset of Water-Energy-Carbon Fluxes Across the Soil-Plant-Atmosphere Continuum

## Data Availability

The code of the STEMMUS-SCOPE model can be acquired from https://github.com/EcoExtreML/STEMMUS_SCOPE. And the script for preparing forcing data and setting the initial conditions can be acquired from https://github.com/EcoExtreML/STEMMUS_SCOPE_Processing. It is also available at: 10.5281/zenodo.15130521.

## References

[1] He, N., Guo, W., Lan, J., Yu, Z. & Wang, H. The impact of human activities and climate change on the eco-hydrological processes in the Yangtze River basin. *Journal of Hydrology: Regional Studies* **53**10.1016/j.ejrh.2024.101753 (2024).

[2] Jiang, S. *et al*. Quantifying the impact of climate change and human activities on the eco-hydrological regimes of the Weihe River Basin, Northwest China. *Hydrology Research* **54**, 49–64, 10.2166/nh.2022.194 (2023).

[3] Jiao, Y. *et al*. Impact of vegetation dynamics on hydrological processes in a semi-arid basin by using a land surface-hydrology coupled model. *Journal of Hydrology* **551**, 116–131, 10.1016/j.jhydrol.2017.05.060 (2017).

[4] Qin, Y. *et al*. Impacts of climate warming on the frozen ground and eco-hydrology in the Yellow River source region, China. *Sci Total Environ* **605-606**, 830–841, 10.1016/j.scitotenv.2017.06.188 (2017). 28683427 10.1016/j.scitotenv.2017.06.188

[5] Wang, Y. P. *et al*. Diagnosing errors in a land surface model (CABLE) in the time and frequency domains. *Journal of Geophysical Research* **116**10.1029/2010jg001385 (2011).

[6] Martens, B. *et al*. GLEAM v3: satellite-based land evaporation and root-zone soil moisture. *Geoscientific Model Development* **10**, 1903–1925, 10.5194/gmd-10-1903-2017 (2017).

[7] Kang, M. *et al*. New Gap-Filling Strategies for Long-Period Flux Data Gaps Using a Data-Driven Approach. *Atmosphere* **10**10.3390/atmos10100568 (2019).

[8] Fisher, J. B. *et al*. The future of evapotranspiration: Global requirements for ecosystem functioning, carbon and climate feedbacks, agricultural management, and water resources. *Water Resources Research* **53**, 2618–2626, 10.1002/2016wr020175 (2017).

[9] Amani, M., Salehi, B., Mahdavi, S., Masjedi, A. & Dehnavi, S. Temperature-Vegetation-soil Moisture Dryness Index (TVMDI). *Remote Sensing of Environment* **197**, 1–14, 10.1016/j.rse.2017.05.026 (2017).

[10] Ford, T. W., Rapp, A. D., Quiring, S. M. & Blake, J. Soil moisture–precipitation coupling: observations from the Oklahoma Mesonet and underlying physical mechanisms. *Hydrology and Earth System Sciences* **19**, 3617–3631, 10.5194/hess-19-3617-2015 (2015).

[11] Seneviratne, S. I. *et al*. Investigating soil moisture–climate interactions in a changing climate: A review. *Earth-Science Reviews* **99**, 125–161, 10.1016/j.earscirev.2010.02.004 (2010).

[12] Seneviratne, S. I. & Orth, R. Variability of Soil Moisture and Sea Surface Temperatures Similarly Important for Warm-Season Land Climate in the Community Earth System Model. *Journal of Climate* **30**, 2141–2162, 10.1175/jcli-d-15-0567.1 (2017).

[13] Lievens, H. *et al*. SMOS soil moisture assimilation for improved hydrologic simulation in the Murray Darling Basin, Australia. *Remote Sensing of Environment*10.1016/j.rse.2015.06.025 (2015).

[14] Colliander, A. *et al*. Validation of SMAP surface soil moisture products with core validation sites. *Remote Sensing of Environment* **191**, 215–231, 10.1016/j.rse.2017.01.021 (2017).

[15] Dorigo, W. A. *et al*. Evaluation of the ESA CCI soil moisture product using ground-based observations. *Remote Sensing of Environment* **162**, 380–395, 10.1016/j.rse.2014.07.023 (2015).

[16] Du, E., Vittorio, A. D. & Collins, W. D. Evaluation of hydrologic components of community land model 4 and bias identification. *International Journal of Applied Earth Observation and Geoinformation* **48**, 5–16, 10.1016/j.jag.2015.03.013 (2016).

[17] Zeng, Y. *et al*. Blending Satellite Observed, Model Simulated, and *in Situ* Measured Soil Moisture over Tibetan Plateau. *Remote Sensing* **8**10.3390/rs8030268 (2016).

[18] Zhuang, R., Zeng, Y., Manfreda, S. & Su, Z. Quantifying Long-Term Land Surface and Root Zone Soil Moisture over Tibetan Plateau. *Remote Sensing* **12**10.3390/rs12030509 (2020).

[19] Dumedah, G. & Coulibaly, P. Evolutionary assimilation of streamflow in distributed hydrologic modeling using *in-situ* soil moisture data. *Advances in Water Resources* **53**, 231–241, 10.1016/j.advwatres.2012.07.012 (2013).

[20] Massari, C., Brocca, L., Tarpanelli, A. & Moramarco, T. Data Assimilation of Satellite Soil Moisture into Rainfall-Runoff Modelling: A Complex Recipe? *Remote Sensing* **7**, 11403–11433, 10.3390/rs70911403 (2015).

[21] Pastorello, G. *et al*. The FLUXNET2015 dataset and the ONEFlux processing pipeline for eddy covariance data. *Sci Data* **7**, 225, 10.1038/s41597-020-0534-3 (2020). 32647314 10.1038/s41597-020-0534-3 PMC7347557

[22] Mahabbati, A. *et al*. A comparison of gap-filling algorithms for eddy covariance fluxes and their drivers. *Geoscientific Instrumentation, Methods and Data Systems* **10**, 123–140, 10.5194/gi-10-123-2021 (2021).

[23] Ukkola, A. M., Abramowitz, G. & De Kauwe, M. G. A flux tower dataset tailored for land model evaluation. *Earth System Science Data* **14**, 449–461, 10.5194/essd-14-449-2022 (2022).

[24] Niu, G. Y. *et al*. Enhancing the Noah‐MP Ecosystem Response to Droughts With an Explicit Representation of Plant Water Storage Supplied by Dynamic Root Water Uptake. *Journal of Advances in Modeling Earth Systems* **12**10.1029/2020ms002062 (2020).

[25] Wang, Y. *et al*. Integrated modeling of canopy photosynthesis, fluorescence, and the transfer of energy, mass, and momentum in the soil–plant–atmosphere continuum (STEMMUS–SCOPE v1.0.0). *Geoscientific Model Development* **14**, 1379–1407, 10.5194/gmd-14-1379-2021 (2021).

[26] Van der Tol, C., Verhoef, W., Timmermans, J., Verhoef, A. & Su, Z. An integrated model of soil-canopy spectral radiances, photosynthesis, fluorescence, temperature and energy balance. *Biogeosciences* **6**, 3109–3129, 10.5194/bg-6-3109-2009 (2009).

[27] Yu, Z. *et al*. Enhanced observations from an optimized soil-canopy-photosynthesis and energy flux model revealed evapotranspiration-shading cooling dynamics of urban vegetation during extreme heat. *Remote Sensing of Environment* **305**10.1016/j.rse.2024.114098 (2024).

[28] Zhang, Y. *et al*. Estimation of vegetation photosynthetic capacity from space-based measurements of chlorophyll fluorescence for terrestrial biosphere models. *Glob Chang Biol* **20**, 3727–3742, 10.1111/gcb.12664 (2014). 24953485 10.1111/gcb.12664

[29] Zhu, K. *et al*. Characterization of the layered SIF distribution through hyperspectral observation and SCOPE modeling for a subtropical evergreen forest. *ISPRS Journal of Photogrammetry and Remote Sensing* **201**, 78–91, 10.1016/j.isprsjprs.2023.05.014 (2023).

[30] Zeng, Y. & Su, Z. *STEMMUS: Simultaneous Transfer of Engery, Mass and Momentum in Unsaturated Soil* Doctor thesis, University of Twente (2013).

[31] Yu, L., Zeng, Y., Wen, J. & Su, Z. Liquid-Vapor-Air Flow in the Frozen Soil. *Journal of Geophysical Research: Atmospheres*10.1029/2018jd028502 (2018).

[32] Zeng, Y., Su, Z., Wan, L. & Wen, J. Numerical analysis of air-water-heat flow in unsaturated soil: Is it necessary to consider airflow in land surface models? *Journal of Geophysical Research* **116**10.1029/2011jd015835 (2011).

[33] Zeng, Y., Su, Z., Wan, L. & Wen, J. A simulation analysis of the advective effect on evaporation using a two-phase heat and mass flow model. *Water Resources Research* **47**10.1029/2011wr010701 (2011).

[34] Montzka, C., Herbst, M., Weihermüller, L., Verhoef, A. & Vereecken, H. A global data set of soil hydraulic properties and sub-grid variability of soil water retention and hydraulic conductivity curves. *Earth System Science Data* **9**, 529–543, 10.5194/essd-9-529-2017 (2017).

[35] Shangguan, W., Dai, Y., Duan, Q., Liu, B. & Yuan, H. A global soil data set for earth system modeling. *Journal of Advances in Modeling Earth Systems* **6**, 249–263, 10.1002/2013ms000293 (2014).

[36] Lawrence, D. *et al*. *Technical Description of version 5.0 of the Community LandModel*. (2022).

[37] Noilhan, J. A Simple Parameterization of Land Surface Processes for Meteorological Models. *Mon.wea.rev* **117**, 536–549, 10.1175/1520-0493(1989)1172.0.CO;2 (1989).

[38] Muñoz-Sabater, J. *et al*. ERA5-Land: a state-of-the-art global reanalysis dataset for land applications. *Earth System Science Data* **13**, 4349–4383, 10.5194/essd-13-4349-2021 (2021).

[39] Vermote, E. & Vermeulen, A. Atmospheric correction algorithm: spectral reflectances (MOD09). *ATBD version* **4**, 1–107 (1999).

[40] Johnson, J. E. & Berry, J. A. The role of Cytochrome b6f in the control of steady-state photosynthesis: a conceptual and quantitative model. *Photosynth Res* **148**, 101–136, 10.1007/s11120-021-00840-4 (2021). 33999328 10.1007/s11120-021-00840-4 PMC8292351

[41] Wang, Y. *et al*. STEMMUS-SCOPE for PLUMBER2: A Physically Consistent Dataset Across the Soil-Plant-Atmosphere Continuum (v1.0.3), *Zenodo*, 10.5281/zenodo.7737321 (2025).

[42] Gupta, H. V., Kling, H., Yilmaz, K. K. & Martinez, G. F. Decomposition of the mean squared error and NSE performance criteria: Implications for improving hydrological modelling. *Journal of Hydrology* **377**, 80–91, 10.1016/j.jhydrol.2009.08.003 (2009).

[43] Tang, E. *et al*. Understanding the effects of revegetated shrubs on fluxes of energy, water, and gross primary productivity in a desert steppe ecosystem using the STEMMUS–SCOPE model. *Biogeosciences* **21**, 893–909, 10.5194/bg-21-893-2024 (2024).

[44] Han, Q. *et al*. Global long term daily 1 km surface soil moisture dataset with physics informed machine learning. *Sci Data* **10**, 101, 10.1038/s41597-023-02011-7 (2023). 36805459 10.1038/s41597-023-02011-7 PMC9938112

[45] Yu, L., Wen, J., Chang, C., Frankenberg, C. & Sun, Y. High‐resolution global contiguous SIF of OCO‐2. *Geophysical Research Letters* **46**, 1449–1458 (2019).

[46] Anav, A. *et al*. Evaluating the Land and Ocean Components of the Global Carbon Cycle in the CMIP5 Earth System Models. *Journal of Climate* **26**, 6801–6843, 10.1175/jcli-d-12-00417.1 (2013).

[47] Bastrikov, V. *et al*. Land surface model parameter optimisation using *in situ* flux data: comparison of gradient-based versus random search algorithms (a case study using ORCHIDEE v1.9.5.2). *Geoscientific Model Development* **11**, 4739–4754, 10.5194/gmd-11-4739-2018 (2018).

[48] Knutti, R. *et al*. Uncertainties in CMIP5 Climate Projections due to Carbon Cycle Feedbacks. *Journal of Climate* **27**, 511–526, 10.1175/jcli-d-12-00579.1 (2014).

[49] Sitch, S. *et al*. Recent trends and drivers of regional sources and sinks of carbon dioxide. *Biogeosciences* **12**, 653–679, 10.5194/bg-12-653-2015 (2015).

[50] Prikaziuk, E., Migliavacca, M., Su, Z. & van der Tol, C. Simulation of ecosystem fluxes with the SCOPE model: Sensitivity to parametrization and evaluation with flux tower observations. *Remote Sensing of Environment* **284**, 10.1016/j.rse.2022.113324 (2023).

[51] Forzieri, G. *et al*. Evaluating the Interplay Between Biophysical Processes and Leaf Area Changes in Land Surface Models. *J Adv Model Earth Syst* **10**, 1102–1126, 10.1002/2018MS001284 (2018). 30034575 10.1002/2018MS001284 PMC6049881

[52] Huang, A., Shen, R., Shi, C. & Sun, S. Effects of satellite LAI data on modelling land surface temperature and related energy budget in the Noah-MP land surface model. *Journal of Hydrology* **613**10.1016/j.jhydrol.2022.128351 (2022).

[53] De Kauwe, M. G., Disney, M. I., Quaife, T., Lewis, P. & Williams, M. An assessment of the MODIS collection 5 leaf area index product for a region of mixed coniferous forest. *Remote Sensing of Environment* **115**, 767–780, 10.1016/j.rse.2010.11.004 (2011).

[54] Peters‐Lidard, C. D. *et al*. Role of precipitation uncertainty in the estimation of hydrologic soil properties using remotely sensed soil moisture in a semiarid environment. *Water Resources Research* **44**10.1029/2007wr005884 (2008).

[55] Zhao, N. *et al*. Investigation of Rainfall-Runoff Processes and Soil Moisture Dynamics in Grassland Plots under Simulated Rainfall Conditions. *Water* **6**, 2671–2689, 10.3390/w6092671 (2014).

[56] Zhong, S., Yang, T., Qian, Y., Zhu, J. & Wu, F. Temporal and spatial variations of soil moisture – Precipitation feedback in East China during the East Asian summer monsoon period: A sensitivity study. *Atmospheric Research* **213**, 163–172, 10.1016/j.atmosres.2018.05.014 (2018).

[57] Komatsu, H., Kumagai, T. O. & Hotta, N. Is surface conductance theoretically independent of reference height. *Hydrological Processes* **19**, 339–347, 10.1002/hyp.5760 (2005).

[58] Chen, J. M. *et al*. Global datasets of leaf photosynthetic capacity for ecological and earth system research. *Earth System Science Data* **14**, 4077–4093, 10.5194/essd-14-4077-2022 (2022).

[59] Croft, H. *et al*. The global distribution of leaf chlorophyll content. *Remote Sensing of Environment* **236**10.1016/j.rse.2019.111479 (2020).

[60] He, L. *et al*. Diverse photosynthetic capacity of global ecosystems mapped by satellite chlorophyll fluorescence measurements. *Remote Sens Environ* **232**10.1016/j.rse.2019.111344 (2019). 10.1016/j.rse.2019.111344 PMC7608051 33149371

[61] Romano, N. Soil moisture at local scale: Measurements and simulations. *Journal of Hydrology* **516**, 6–20, 10.1016/j.jhydrol.2014.01.026 (2014).

[62] S.U, S. L., Singh, D. N. & Shojaei Baghini, M. A critical review of soil moisture measurement. *Measurement* **54**, 92–105, 10.1016/j.measurement.2014.04.007 (2014).

[63] Spaans, E. J. A. & Baker, J. M. Examining the use of time domain reflectometry for measuring liquid water content in frozen soil. *Water Resources Research* **31**, 2917–2925, 10.1029/95wr02769 (2010).

[64] De Kauwe, M. G. *et al*. Identifying areas at risk of drought-induced tree mortality across South-Eastern Australia. *Glob Chang Biol* **26**, 5716–5733, 10.1111/gcb.15215 (2020). 32512628 10.1111/gcb.15215

[65] De Kauwe, M. G. *et al*. Do land surface models need to include differential plant species responses to drought? Examining model predictions across a mesic-xeric gradient in Europe. *Biogeosciences* **12**, 7503–7518, 10.5194/bg-12-7503-2015 (2015).

[66] Eller, C. B. *et al*. Stomatal optimization based on xylem hydraulics (SOX) improves land surface model simulation of vegetation responses to climate. *New Phytol* **226**, 1622–1637, 10.1111/nph.16419 (2020). 31916258 10.1111/nph.16419 PMC7318565

[67] Medlyn, B. E., De Kauwe, M. G. & Duursma, R. A. New developments in the effort to model ecosystems under water stress. *New Phytol* **212**, 5–7, 10.1111/nph.14082 (2016). 27558747 10.1111/nph.14082

[68] Xu, X., Medvigy, D., Powers, J. S., Becknell, J. M. & Guan, K. Diversity in plant hydraulic traits explains seasonal and inter-annual variations of vegetation dynamics in seasonally dry tropical forests. *New Phytol* **212**, 80–95, 10.1111/nph.14009 (2016). 27189787 10.1111/nph.14009

[69] Kennedy, D. *et al*. Implementing Plant Hydraulics in the Community Land Model, Version 5. *Journal of Advances in Modeling Earth Systems* **11**, 485–513, 10.1029/2018ms001500 (2019).

[70] Li, L. *et al*. Representation of plant hydraulics in the Noah‐MP land surface model: Model development and multiscale evaluation. *Journal of Advances in Modeling Earth Systems* **13**, e2020MS002214 (2021).

[71] Song, Z. *et al*. Investigating Plant Responses to Water Stress via Plant Hydraulics Pathway. *EGUsphere* **2024**, 1–25, 10.5194/egusphere-2024-2940 (2024).

[72] Xie, S., Mo, X., Liu, S. & Hu, S. Plant Hydraulics Improves Predictions of ET and GPP Responses to Drought. *Water Resources Research* **59**10.1029/2022wr033402 (2023).

[73] Reichstein, M. *et al*. On the separation of net ecosystem exchange into assimilation and ecosystem respiration: review and improved algorithm. *Global Change Biology* **11**, 1424–1439, 10.1111/j.1365-2486.2005.001002.x (2005).

[74] Peters, W. *et al*. Increased water-use efficiency and reduced CO(2) uptake by plants during droughts at a continental-scale. *Nat Geosci* **11**, 744–748, 10.1038/s41561-018-0212-7 (2018). 30319710 10.1038/s41561-018-0212-7 PMC6179136

[75] Wu, S. *et al*. Estimating winter wheat yield by assimilation of remote sensing data with a four-dimensional variation algorithm considering anisotropic background error and time window. *Agricultural and Forest Meteorology* **301-302**10.1016/j.agrformet.2021.108345 (2021).

[76] Poyatos, R. *et al*. Global transpiration data from sap flow measurements: the SAPFLUXNET database. *Earth System Science Data* **13**, 2607–2649, 10.5194/essd-13-2607-2021 (2021).

[77] Dorigo, W. A. *et al*. The International Soil Moisture Network: a data hosting facility for global *in situ* soil moisture measurements. *Hydrology and Earth System Sciences* **15**, 1675–1698, 10.5194/hess-15-1675-2011 (2011).

[78] Chen, J. M., Liu, J., Cihlar, J. & Goulden, M. L. Daily canopy photosynthesis model through temporal and spatial scaling for remote sensing applications. *Ecological Modelling* **124**, 99–119, 10.1016/S0304-3800(99)00156-8 (1999).

[79] Tramontana, G., Jung, M., Camps-Valls, G., Ichii, K. & Papale, D. Predicting carbon dioxide and energy fluxes across global FLUXNET sites with regression algorithms. *Biogeosciences*, 1-33 10.5194/bg-13-4291-2016 (2016).

[80] Zhang, Z. *et al*. Reduction of structural impacts and distinction of photosynthetic pathways in a global estimation of GPP from space-borne solar-induced chlorophyll fluorescence. *Remote Sensing of Environment* **240**10.1016/j.rse.2020.111722 (2020).

[81] Chen, X., Su, Z., Ma, Y., Trigo, I. & Gentine, P. Remote Sensing of Global Daily Evapotranspiration based on a Surface Energy Balance Method and Reanalysis Data. *Journal of Geophysical Research: Atmospheres* **126**10.1029/2020jd032873 (2021).

[82] Zheng, C., Li, J., Hu, G., Jing, L. & Li, Z. in *Geoscience & Remote Sensing Symposium*.

[83] Al-Yaari, A. *et al*. Global-scale evaluation of two satellite-based passive microwave soil moisture datasets (SMOS and AMSR-E) with respect to Land Data Assimilation System estimates. *Remote Sensing of Environment* **149**, 181–195, 10.1016/j.rse.2014.04.006 (2014).

[84] McNairn, H. *et al*. The Soil Moisture Active Passive Validation Experiment 2012 (SMAPVEX12): Prelaunch Calibration and Validation of the SMAP Soil Moisture Algorithms. *IEEE Transactions on Geoscience and Remote Sensing* **53**, 2784–2801, 10.1109/tgrs.2014.2364913 (2015).

[85] Bowling, L. & Polcher, J. *The ALMA data exchange convention*, https://web.lmd.jussieu.fr/~polcher/ALMA/ (2001).

