# Supplementary material for: A physically consistent dataset of water-energy-carbon fluxes across the Soil-Plant-Atmosphere Continuum
Source: Sci Data. 2025 Jul 4;12:1146. doi: 10.1038/s41597-025-05386-x (PMC12227580; doi:10.1038/s41597-025-05386-x)
Supplement: Supplementary file 1 — Supplement of A Physically Consistent Dataset of Water-Energy-Carbon Fluxes Across the Soil-Plant-Atmosphere Continuum [file 41597_2025_5386_MOESM1_ESM.docx]

*Supplement of*

**A Physically Consistent Dataset of Water-Energy-Carbon Fluxes Across the Soil-Plant-Atmosphere Continuum**

**Yunfei Wang et al.**

*Correspondence to*: Zhongbo Su (z.su@utwente.nl) and Qiang Yu (yuq@nwafu.edu.cn)

## S.1. Water Stress Factor in SCOPE

The C4 Photosynthesis is calculated in the SCOPE model as the minimum of three processes^1^; (1) carboxylation rate limited by Ribulose biphosphate-carboxylase-oxygenase activity (known as Rubisco (enzyme)-limited, *V_c_*, described in Eq. (S1); (2) carboxylation rate limited by Ribulose 1–5 bisphosphate regeneration rate (known as RuBP (electron transport/light)-limited), *V_e_*, described in Eq. (S2); (3) At low CO_2_ concentrations, carboxylation rate limited by intercellular CO_2_ partial pressure (*p_i_*), *V_s_*, described in Eq. (S3).

$V_{c}=V_{cmax}*WSF$ (S1)

$V_{e}=\frac{J}{6}\frac{-b\pm\sqrt{b^{2}-4ac}}{2a}$ (S2)

$V_{s}=p_{i}(k_{p}-\frac{L}{p_{i}})/P$ (S3)

$A_{n}=min\left( V_{c},V_{e},V_{s} \right)$ (S4)

$C_{i}=C_{a}(1-\frac{1}{mRH})$ (S5)

where $V_{cmax}$ is the maximum carboxylation rate (μmol m^−2^ s^−1^), $p_{i}$ is the intercellular CO_2_ partial pressure (Pa), $k_{p}$ is a pseudo-first-order rate constant for PEP carboxylase with respect to $C_{i}$, $P$ is the atmospheric pressure; *A_n_* is the net photosynthesis (μmol m^−2^ s^−1^); *WSF* is the total water stress factor, *J* is the electron transport rate (μmol m^-2^ s^-1^), *C_i_* is the intercellular CO_2_ concentration (μmol m^−3^) and *C_a_* is CO_2_ concentration in the boundary layer (μmol m^−3^), *m* is Ball-Berry parameter and RH is relative humidity at the leaf surface (%).

In the previous study, water stress factor was calculated based on the root zone soil moisture content neglecting the distribution of root length^2^. In this study, water stress factor considered both root length distribution and water content in root zone. We use a sigmoid formulation rather than the piecewise function. The calculations are as follows:

$WSF=\sum_{i=1}^{n} RF\left( i \right)*WSF(i)$ (S6)

$WSF(i)=\frac{1}{1+e^{-100*\theta_{sat}\left( SM(i)-\frac{\theta_{f}+\theta_{r}}{2} \right)}}$ (S7)

$\theta_{r}$ is the soil water content at wilting point; $\theta_{f}$ is the soil water content at field capacity; $\theta_{sat}$ is the saturated soil water content; *WSF(i)* is the water stress factor at each soil layer; *RF(i)* is the ratio of *root length* in soil layer *i* and its calculation can be found in the appendix A.4; *SM(i)* is the soil moisture at each soil layer.

### S2. Governing Equations in STEMMUS

### S2.1 Soil water conservation equation

$\frac{\partial}{\partial t}\left( {\rho_{L}\theta}_{L}+{\rho_{V}\theta}_{V} \right)=-\frac{\partial}{\partial z}\left( q_{Lh}+q_{LT}+q_{La}+q_{Vh}+q_{VT}+q_{Va} \right)-S=\rho_{L}\frac{\partial}{\partial z}\left[ K\left( \frac{\partial h}{\partial z}+1 \right)+D_{TD}\frac{\partial T_{s}}{\partial z}+\frac{K}{\gamma_{w}}\frac{\partial P_{g}}{\partial z} \right]+\frac{\partial}{\partial z}\left[ D_{Vh}\frac{\partial h}{\partial z}+D_{VT}\frac{\partial T_{s}}{\partial z}+D_{Va}\frac{\partial P_{g}}{\partial z} \right]-S$ (S8)

where $\rho_{L}$, $\rho_{V}$ (kg m^−3^) are the density of liquid water, water vapor, respectively; *q_L_*, *q_V_* (m^3^ m^−3^) are the volumetric water content (liquid and water vapor, respectively); z (m) is the vertical space coordinate (positive upwards); *S* (cm s^−1^) is the sink term for the root water extraction. *K* (m s^−1^) is hydraulic conductivity; *h* (cm) is the pressure head; *T_s_* (°C) is the soil temperature; and *P_g_* (Pa) is the mixed pore-air pressure. $\gamma_{w}$ (kg m^-2^ s^-2^) is the specific weight of water. *D_TD_* (kg m^-1^ s^-1^ °C^-1^) is the transport coefficient for adsorbed liquid flow due to temperature gradient; *D_Vh_* (kg m^-2^ s^-1^) is the isothermal vapor conductivity; and *D_VT_* (kg m^-1^ s^-1^ °C^-1^) is the thermal vapor diffusion coefficient. *D_Va_* is the advective vapor transfer coefficient^3,4^. $q_{Lh}$, $q_{LT}$, and $q_{La}$(kg m^-2^ s^-1^) are the liquid water fluxes driven by the gradient of matric potential, temperature, and air pressure, respectively. $q_{Vh}$, $q_{VT}$, and $q_{Va}$ (kg m^-2^ s^-1^) are the water vapor fluxes driven by the gradient of matric potential, temperature, and air pressure, respectively.

### S2.2 Dry air conservation equation

$\frac{\partial}{\partial t}\left[ \varepsilon\rho_{da}\left( {S_{a}+H}_{c}S_{L} \right) \right]=\frac{\partial}{\partial z}\left[ D_{e}\frac{\partial\rho_{da}}{\partial z}+\rho_{da}\frac{S_{a}K_{g}}{\mu_{a}}\frac{\partial P_{g}}{\partial z}-H_{c}\rho_{da}\frac{q_{L}}{\rho_{L}}+\left( \theta_{a}D_{Vg} \right)\frac{\partial\rho_{da}}{\partial z} \right]$ (S9)

where $\varepsilon$ is the porosity; $\rho_{da}$ (kg m^−3^) is the density of dry air; *S_a_* (=1-*S_L_*) is the degree of air saturation in the soil; *S_L_* (=*θ_L_*/$\varepsilon$) is the degree of saturation in the soil; *H_c_* is Henry’s constant; *D_e_* (m^2^ s^-1^) is the molecular diffusivity of water vapor in soil; *K_g_* (m^2^) is the intrinsic air permeability; *m_a_* ( kg m^-2^ s^-1^) is the air viscosity; *q_L_* (kg m^-2^ s^-1^) is the liquid water flux; $\theta$*_a_* (=$\theta$ _V_) is the volumetric fraction of dry air in the soil; and *D_Vg_* (m^2^ s^-1^) is the gas phase longitudinal dispersion coefficient^3,4^.

### S2.3 Energy balance equation

$\frac{\partial}{\partial t}\left[ \left( {\rho_{s}\theta}_{s}C_{S}+{\rho_{L}\theta}_{L}C_{L}+{\rho_{V}\theta}_{V}C_{V}+{\rho_{da}\theta}_{a}C_{a} \right)\left( T_{s}-T_{r} \right)+{\rho_{V}\theta}_{V}L_{0} \right]-\rho_{L}W\frac{\partial\theta_{L}}{\partial t}=\frac{\partial}{\partial z}\left( \lambda_{eff}\frac{\partial T}{\partial z} \right)-\frac{\partial}{\partial z}{[q_{L}C}_{L}\left( T_{s}-T_{r} \right)+q_{V}({L_{0}+C}_{V}\left( T_{s}-T_{r} \right))+q_{a}C_{a}\left( T_{s}-T_{r} \right)]-C_{L}S\left( T_{s}-T_{r} \right)$ (S10)

where *C_S_*, *C_L_*, *C_V_*, *C_a_* (J kg^−1^ °C^−1^) are the specific heat capacities of solids, liquid, water vapor, and dry air, respectively; $\rho_{s}$(kg m^−3^), $\rho_{L}$ (kg m^−3^), $\rho_{V}$ (kg m^−3^), and $\rho_{da}$ (kg m^−3^) are the density of solids, liquid water, water vapor, and dry air, respectively; $\theta$*_s_* is the volumetric fraction of solids in the soil; $\theta_{L}$, $\theta_{V}$, and$\theta_{a}$ are the volumetric fraction of liquid water, water vapor, and dry air, respectively; *T_r_* (°C) is the reference temperature; *L_0_* (J kg^−1^) is the latent heat of vaporization of water at temperature *T_r_*; *W* (J kg^−1^) is the differential heat of wetting (the amount of heat released when a small amount of free water is added to the soil matrix); and $\lambda_{eff}$ (W m^−1^ °C^−1^) is the effective thermal conductivity of the soil; *q_L_,* *q_V_,* and *q_a_* (kg m^-2^ s^-1^) are the liquid, vapor water and dry air flux.

### S3. Dynamic Root Growth Modelling

### S3.1. Initial vertical root distribution

Initial vertical root distribution is calculated by the root fraction 𝑟_𝑖_ in each soil layer depends on the plant functional type:

$r_{i}=\left( \beta^{{zh}_{i-1}*100}-\beta^{{zh}_{i}*100} \right)$ $for 1\leq i\leq N_{levsoil}$ (S11)

where 𝑧ℎ_𝑖_ (m) is the depth from the soil surface to the interface between layers 𝑖 and 𝑖+1 (𝑧_ℎ,0_, the soil surface), the factor of 100 converts from m to cm, and 𝛽 is a plant-dependent root distribution parameter adopted from^5^.

### S3.2. Root front growth

The depth of the root front is firstly initialized either with the sowing depth for sown crops or with an initial value for transplanted crops or perennial crops. The root front growth stops when it reached certain depth of soil or a physical/chemical obstacle preventing root growth, but also stops when the phenological stopping stage has been reached.

$\Delta Z=\left\{ \begin{matrix} \begin{matrix} 0 \\ \left( T_{air}-T_{min} \right)*RGR \\ \left( T_{max}-T_{min} \right)*RGR \end{matrix} & \begin{matrix} T_{air}<T_{min} \\ T_{min}<T_{air}<T_{max} \\ T_{max}<T_{air} \end{matrix} \end{matrix} \right.$ (S12)

$D_{Z}(t)=D_{Z}(t-1)+\Delta Z$ (S13)

where $\Delta Z$ is root front growth at *t-*th time step; $D_{Z}$ (cm) is root zone depth; $T_{air}$ (^0^C) is air temperature; *T_min_* (^0^C) is the minimum temperature of root growth; *T_max_* (^0^C) is the maximum temperature of root growth; *RGR* (cm ^0^C^-1^ day^-1^) is the root growth rate of root front.

### S3.3. Root length growth

In this study, the root distribution in the root zone was realized via simulating the root length growth in each soil layer^6^.

$\Delta Rl\_tot=\frac{A_{n}*fr_{root}}{R_{C}*R_{D}*\pi*r_{root}^{2}}$ (S14)

$fr_{root}$ is the allocation fraction of net assimilation to root, and $fr_{root}$ is assumed as a function of leaf area index (*LAI*) and root zone water content. $A_{n}$ is the net assimilation rate (*μ*mol m^−2^ s^−1^).$R_{C}$ is ratio of carbon to dry organic matter in root, $R_{D}$ is root density (g m^-3^), and $r_{root}$ is radius of the root (0.15*10^-3^ m), and $\Delta Rl\_tot$ (m m^-3^) is total root length growth.

The limiting factors for allocation are preliminarily computed and they account for root zone soil moisture availability $A_{W}$, and light availability $A_{L}$.

$A_{W}=max\left[ \begin{matrix} 0.1, & min\left( \begin{matrix} 1, & WSF \end{matrix} \right) \end{matrix} \right]$ (S15)

where $WSF$ is the averaged soil moisture stress factor in the root zone.

$A_{L}=max\left[ \begin{matrix} 0.1, & e^{-K_{e} LAI} \end{matrix} \right]$ (S16)

where $K_{e}=0.15$is a constant light extinction coefficient.

$fr_{root}=max\left[ \begin{matrix} r_{min}, & r_{0}\frac{3A_{L}}{A_{L}+2A_{W}} \end{matrix} \right]$ (S17)

where $r_{min}(=0.15$) is the minimum allocation coefficient to fine roots, and $r_{0}$ is a coefficient that indicates the theoretically unstressed allocation to fine roots.

$\Delta Rl(i)=\Delta Rl\_tot*R_{frac}(i)$ (S18)

where $R_{frac}(i)$is the allocation fraction of root growth length in layer *i*, $\Delta Rl(i)$ is the root growth length in layer *i*.

${Rl}_{i}^{t}={Rl}_{i}^{t-1}+\Delta Rl(i)$ (S19)

where ${Rl}_{i}^{t}$ and ${Rl}_{i}^{t-1}$ is the root length of layer *i* at time step *t* and time step *t-1*.

$R_{frac}(i)=\frac{RWU(i)}{{RWU}_{T}}$ (S20)

where ${RWU}_{T}$ is the total root water uptake in root zone,$RWU(i)$ is the root water uptake in soil layer *i*. (here the *RWU* is only considered higher than 0)

### S4. Root water uptake

The equation to calculate root water uptake and transpiration was as follows:

$\sum_{i=1}^{n} \frac{\psi_{s,i}-\psi_{l}}{r_{s,i}+r_{r,i}+r_{x,i}}=\frac{0.622}{P}\frac{\rho_{da}}{\rho_{V}}\left( \frac{e_{l}-e_{a}}{r_{c}+r_{a}} \right)=T$ (S21)

where *ψ_s,i_* is soil water potential of layer *i* (m), *ψ_l_* is leaf water potential (m), *r_s,i_* is the soil hydraulic resistance (s m^−1^), *r_r,i_* is the root resistance to water flow radially across the roots (s m^−1^), and *r_x,i_* is the plant axial resistance to flow from the soil to the leaves (s m^−1^). *e_l_* and *e_a_* are vapor pressure of leaf and the atmosphere (hPa), respectively, and *r_a_* and *r_c_* are aerodynamic resistance and canopy resistance (s m^−1^), respectively. $\rho_{da}$ is the density of dry air (kg m^-3^). $\rho_{V}$ is the density of water vapor. $P$ is the atmospheric pressure (Pa). 0.622 is the ratio of the molar mass of water to air.

$\psi_{s, i}$ is described as a function of soil moisture^7^, and the relevant parameters were shown in Table B.1.

The $r_{s}$ is calculated by (Reid and Huck, 1990) as:

$r_{s}=\frac{1}{B\cdot K\cdot L_{v}\cdot\Delta d}$ (S22)

where *B* is the root length activity factor, *K* is hydraulic conductivity of soil (m s^−1^), $L_{v}$ is root length density (m m^−3^), and $\Delta d$ is the thickness of the soil layer (m). *B* is calculated as:

$B=\frac{2\pi}{ln\left[ \left( \pi R_{D} \right)^{-1/2}/r_{root} \right]}$ (S23)

where $r_{root}$ is root radius (m).

The $r_{r}$ is estimated as^8^:

$r_{r}=\frac{P_{r}\left( \theta_{sat}/\theta\right)}{L_{v}\Delta d}$ (S24)

where $P_{r}$ is root radial resistivity (s m^−1^).

The xylem resistance $r_{x}$ is estimated by^9^:

$r_{x}=\frac{P_{a}Z_{mid}}{0.5fL_{v}}$ (S25)

where $P_{a}$ is root axial resistivity (s m^−3^), $Z_{mid}$ is the depth of the midpoint of soil layer, and $f$ is a fraction defined for a specific depth as the number of roots which connect directly to the stem base to total roots crossing a horizontal plane at that depth. We can consider it equal to 0.22.

The updated root water uptake term is:

$S_{i}=\frac{\psi_{s,i}-\psi_{l}}{r_{s,i}+r_{r,i}+r_{x,i}}$ (S26)

Different from other studies which need to calculate the compensatory water uptake and hydraulic redistribution after calculating the standard water uptake of each soil layer, the sink term in this study is calculated by a physically-based model which contain the effect of root resistance and soil hydraulic resistance rather than only considering the root fraction, so the compensatory uptake and hydraulic redistribution have been implicitly considered when calculating the sink term.


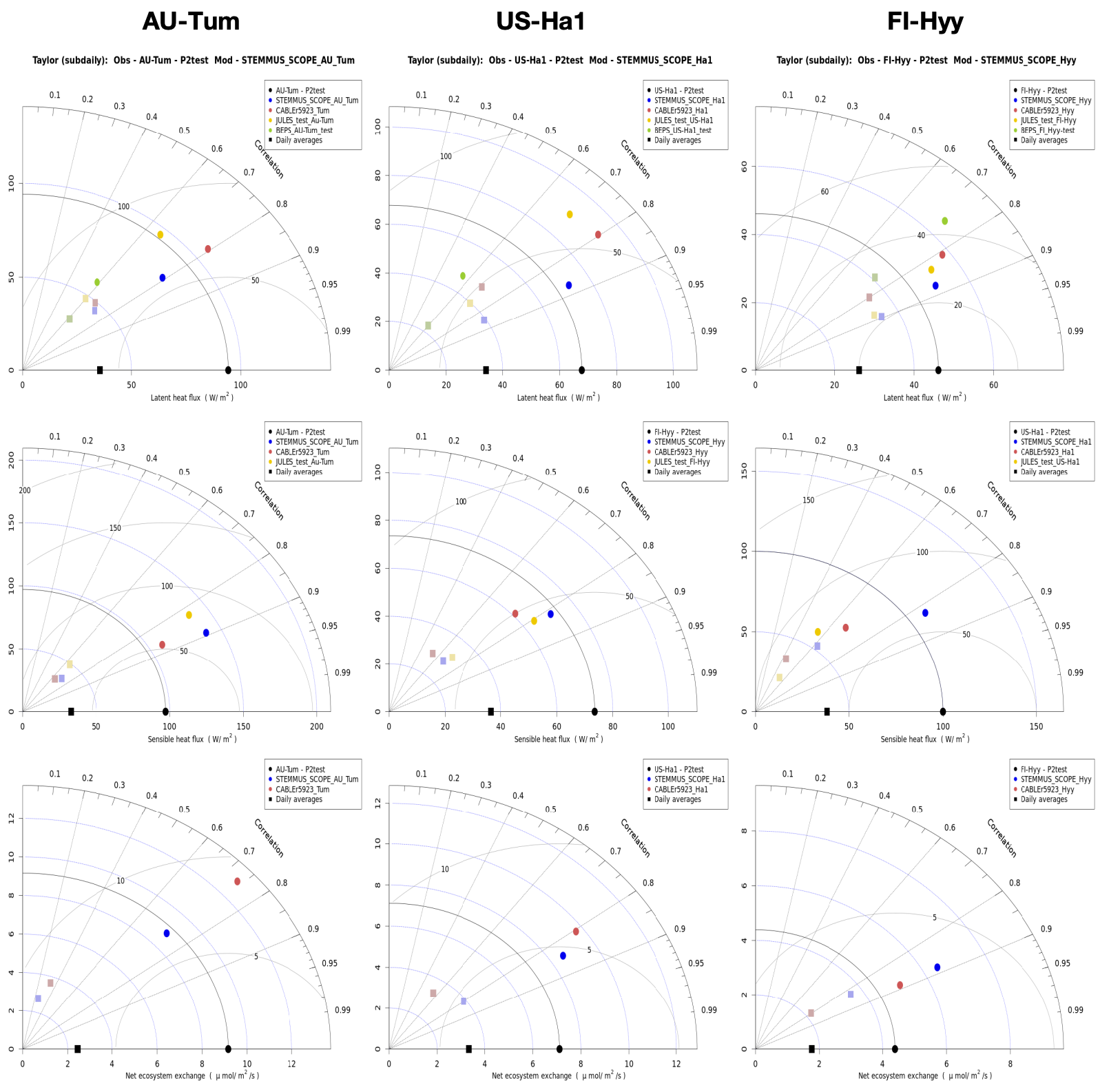


Supplementary Figure 1: Taylor diagram of latent heat flux, sensible heat flux, and net ecosystem exchange of STEMMUS-SCOPE at three forest sites (AU-Tum, US-Ha1, and FI-Hyy)


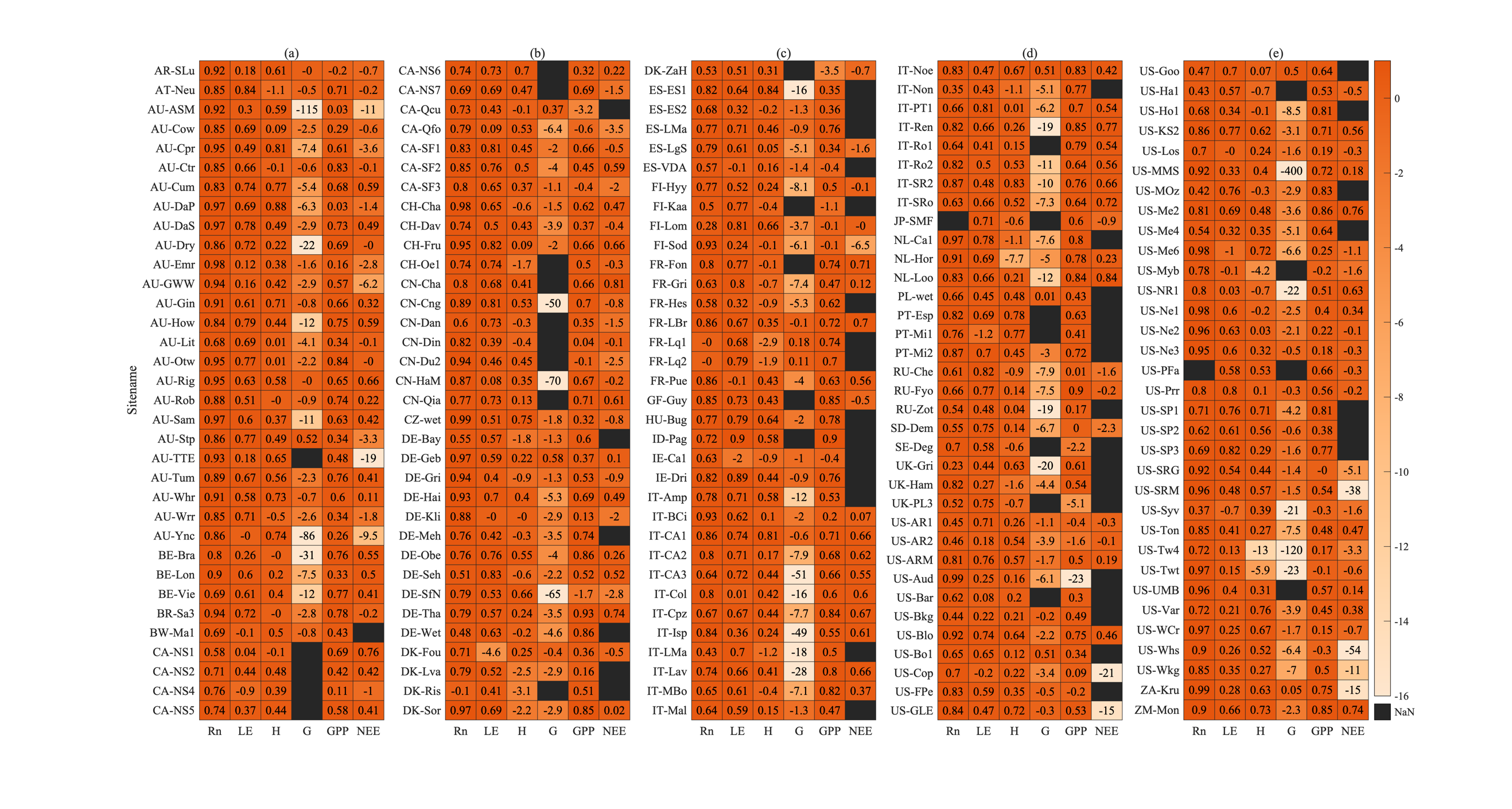


Supplementary Figure 2: The heat plots of the Kling–Gupta efficiency (KGE) between simulation and in-situ measurement of the 170 sites. (NaN means the in-situ measurements are missing and the values lower than -16 was set as -16)


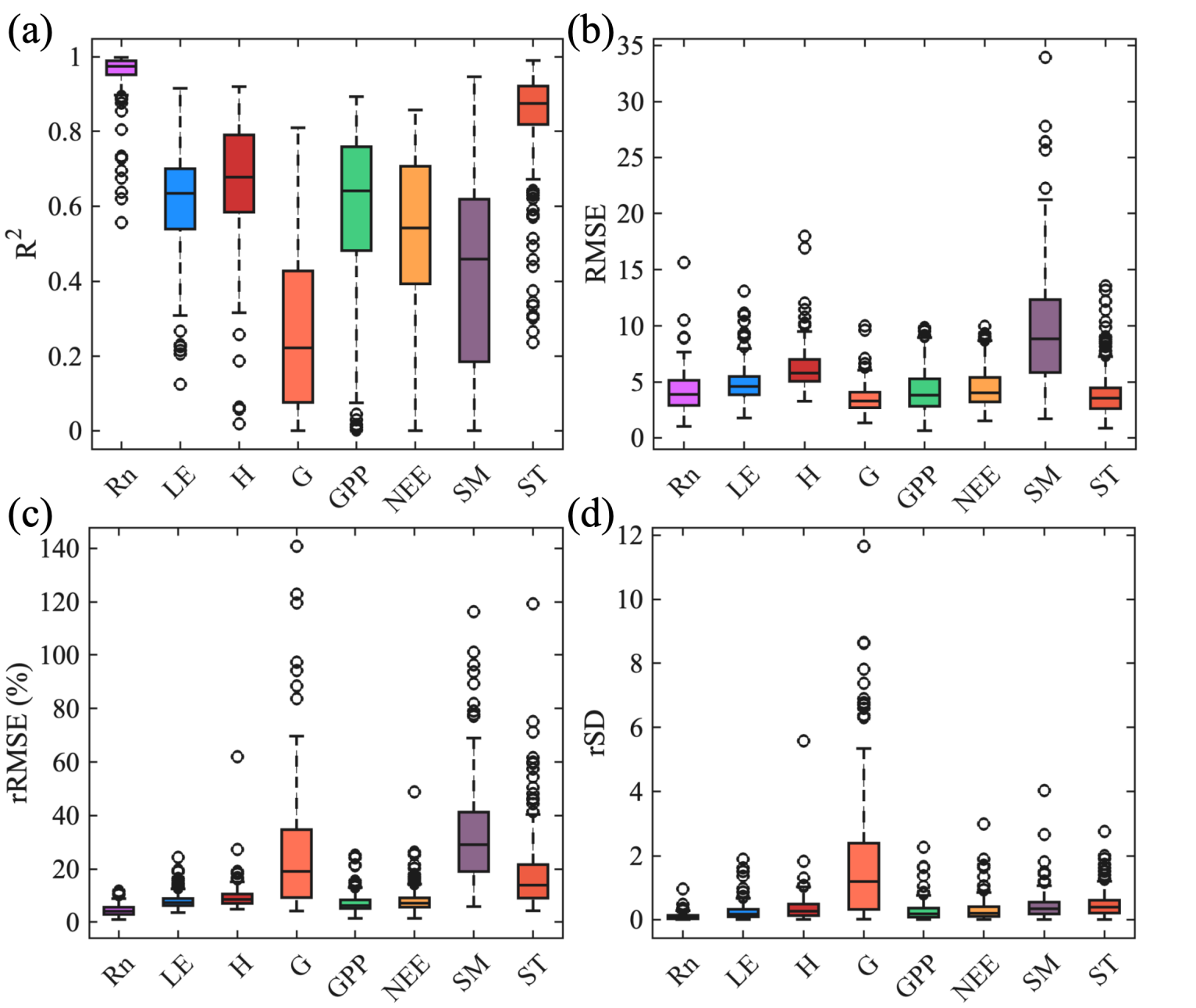


Supplementary Figure 3: Performance (R^2^, RMSE, rRMSE, and SD) of STEMMUS-SCOPE (box plots) for the validation set of observations. The box plots show (from top to bottom) the maximum, 75th percentile, median, 25th percentile, and minimum. The whiskers extend to the most extreme data points not considered outliers, and the outliers are plotted individually using the ‘○’marker symbol. (Unit: Rn: net radiation, 10 W m^-2^; LE: latent heat flux, 10 W m^-2^; H: sensible heat flux, 10 W m^-2^; G: ground heat flux, 10 W m^-2^; GPP: gross primary production, µmol m-2 s^-1^; NEE: net ecosystem exchange, *µ*mol m^-2^ s^-1^; SM: soil moisture, % m^3^ m^-3^; ST: soil temperature, ^o^C)


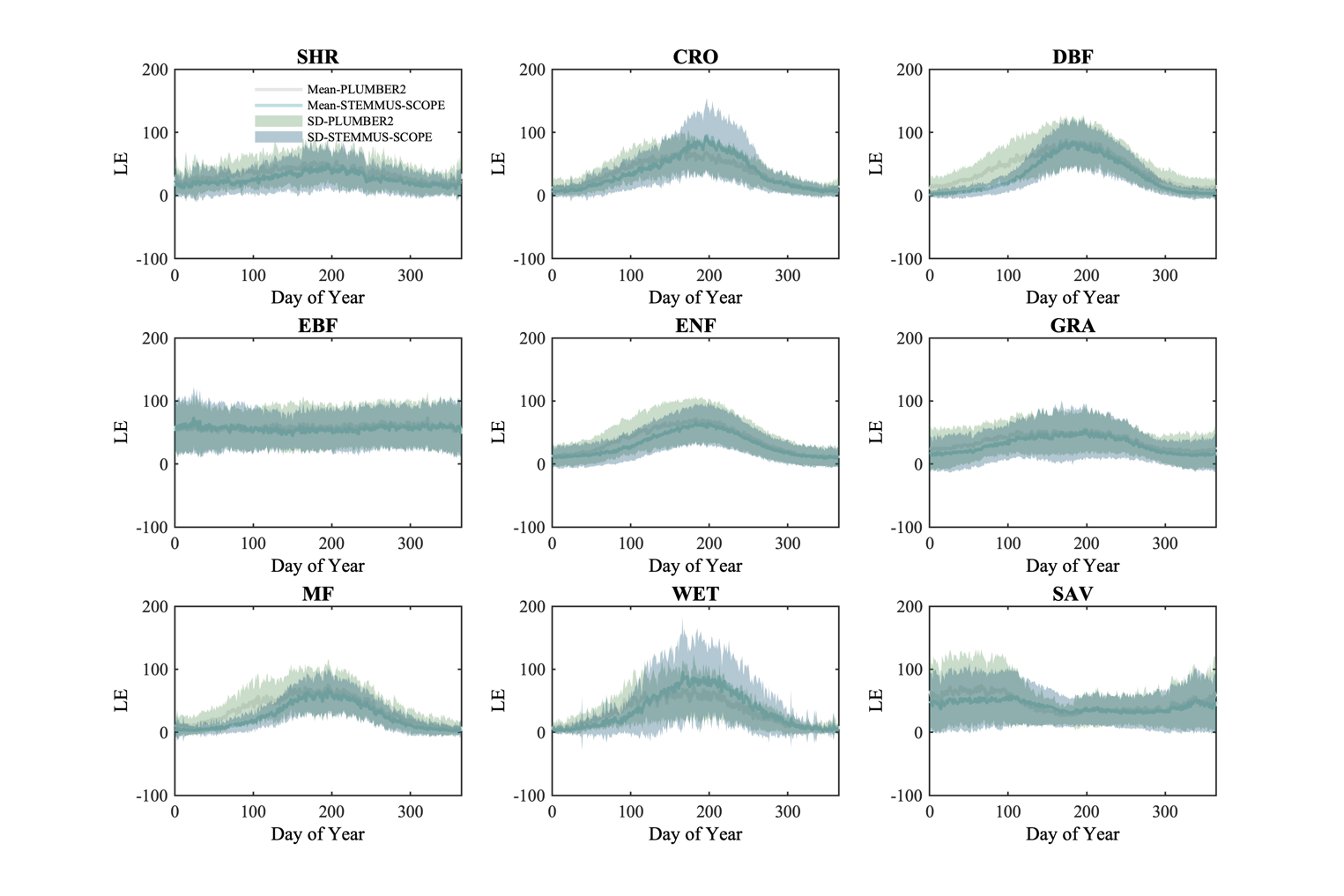


**Supplementary Figure 4: Time series of modeled and observed daily LE at 9 vegetation types.**


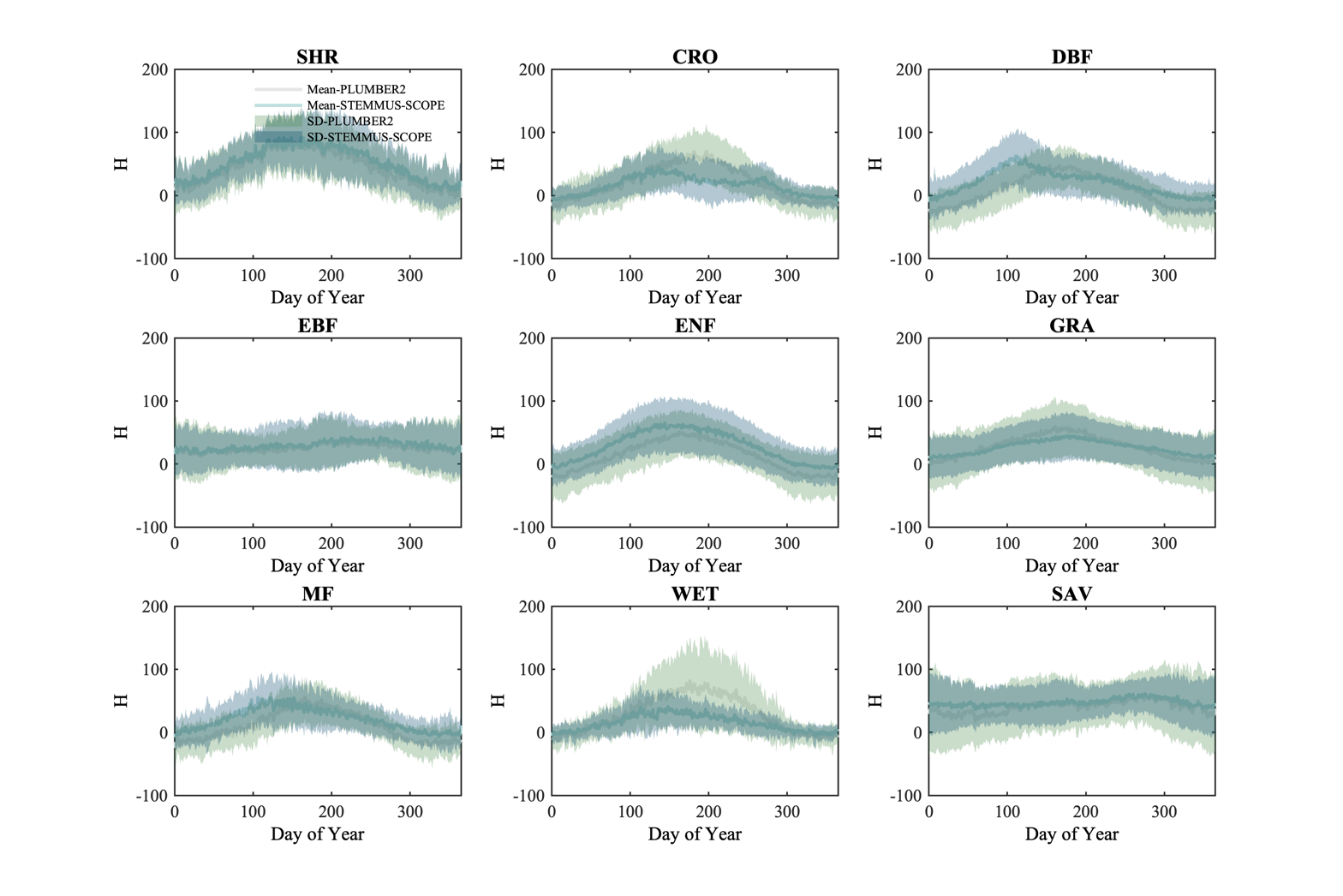


Supplementary Figure 5: Time series of modeled and observed daily H at 9 vegetation types.


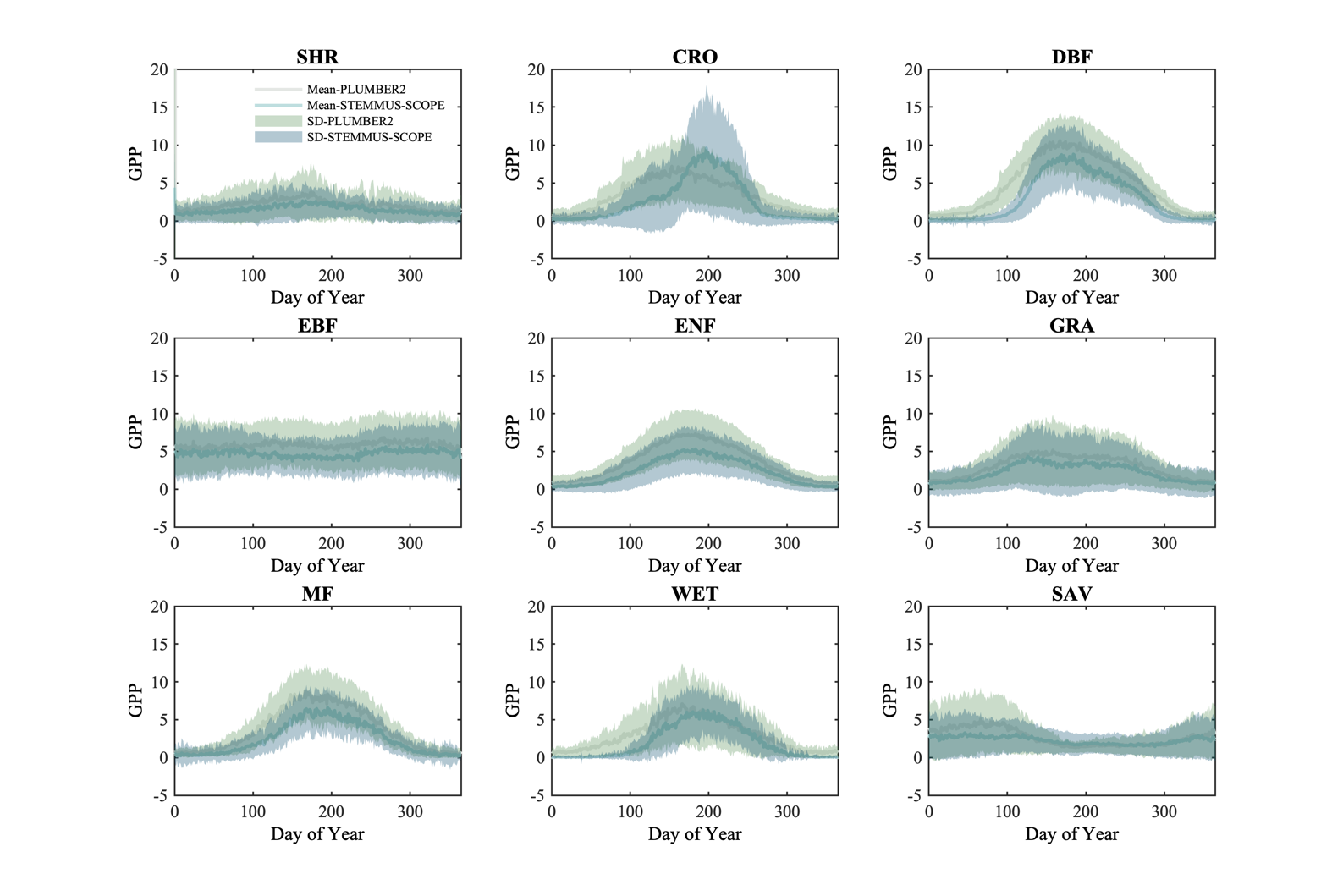


Supplementary Figure 6: Time series of modeled and observed daily GPP at 9 vegetation types.


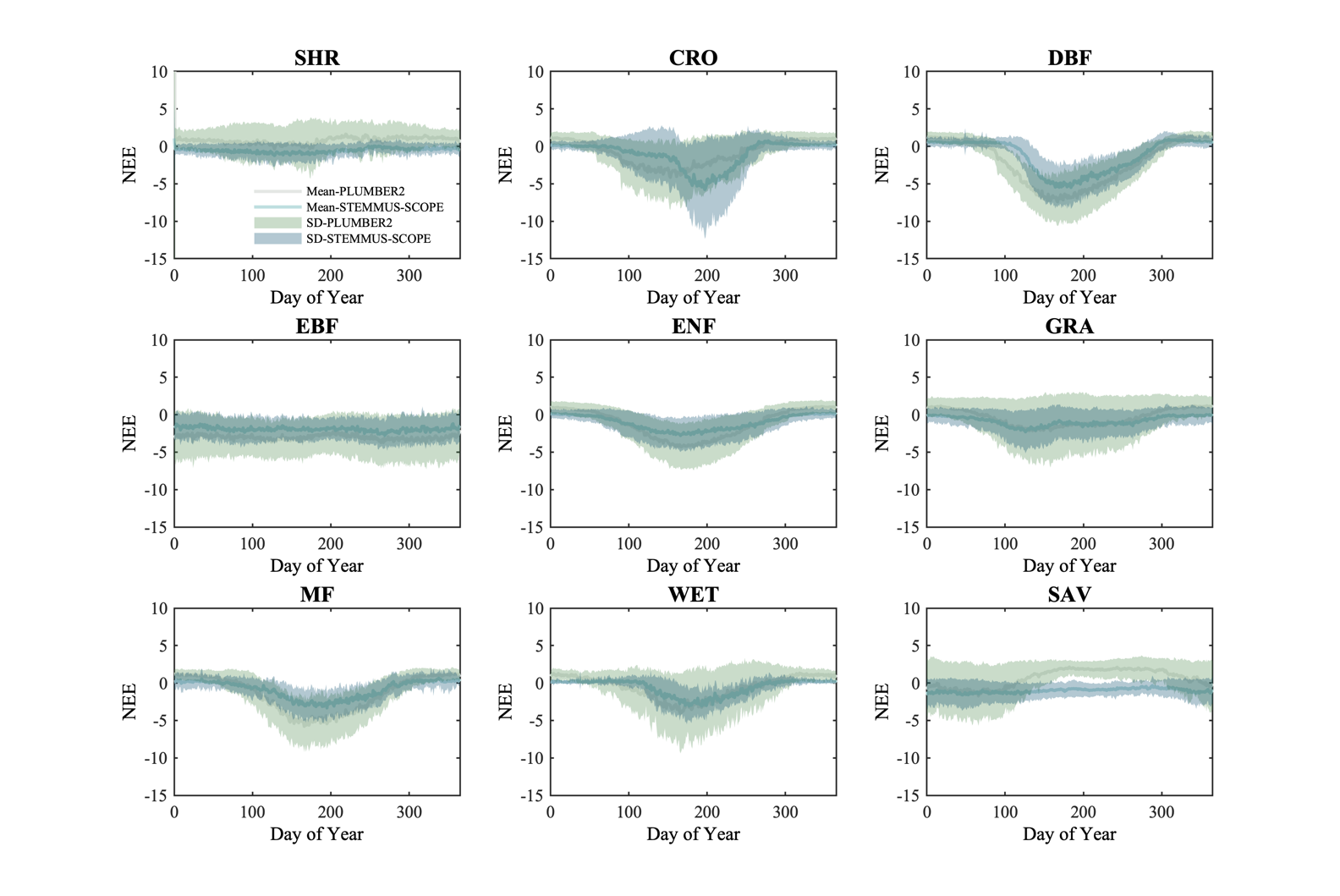


Supplementary Figure 7: Time series of modeled and observed daily NEE at 9 vegetation types.


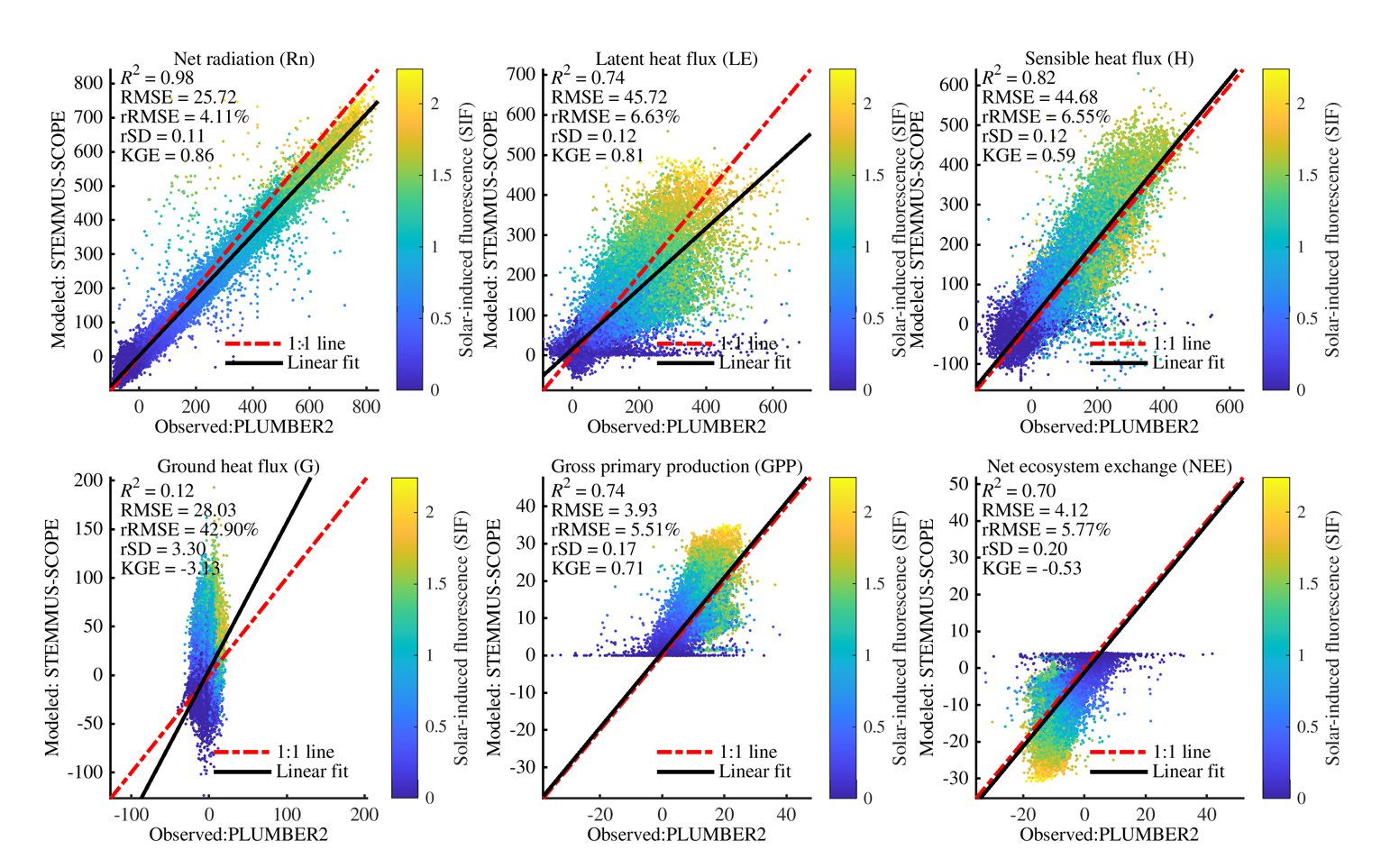


**Supplementary Figure 8: Correlation between modelled and observed energy and carbon fluxes at US-KS2 site, Shrubland**


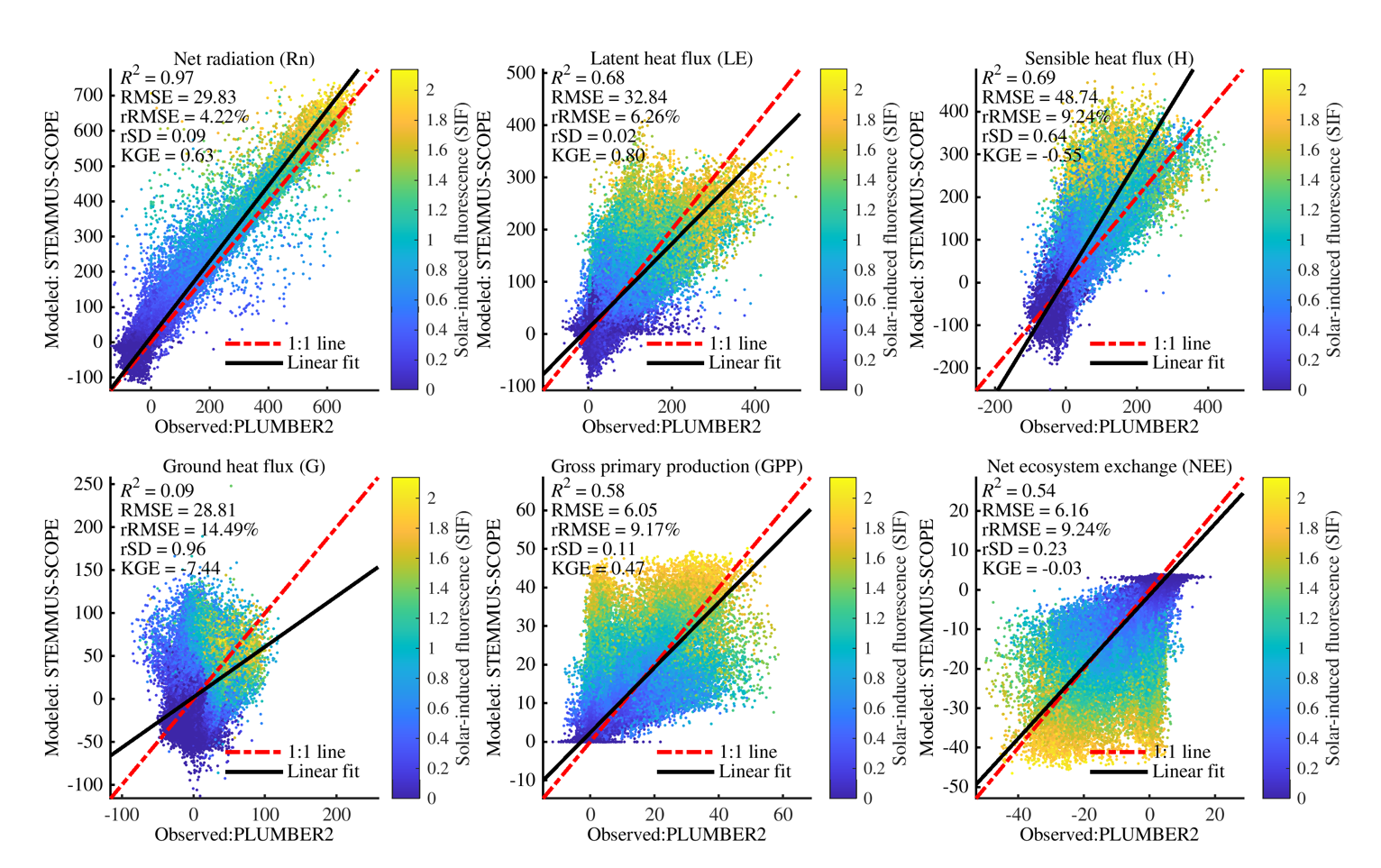


**Supplementary Figure 9: Correlation between modelled and observed energy and carbon fluxes at FR-Gri site, Cropland**


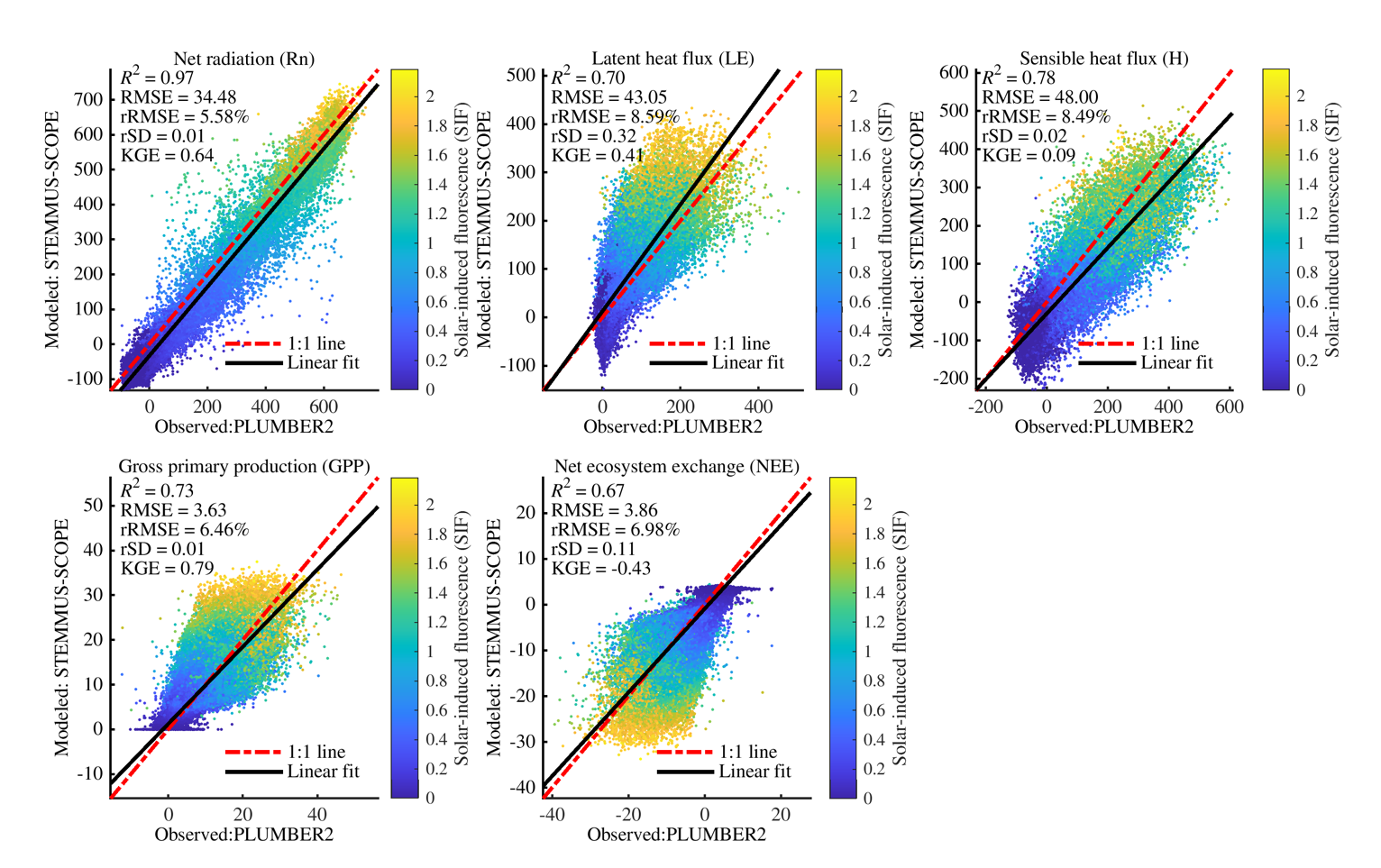


**Supplementary Figure 10: Correlation between modelled and observed energy and carbon fluxes at IT-Ro1 site, DBF**


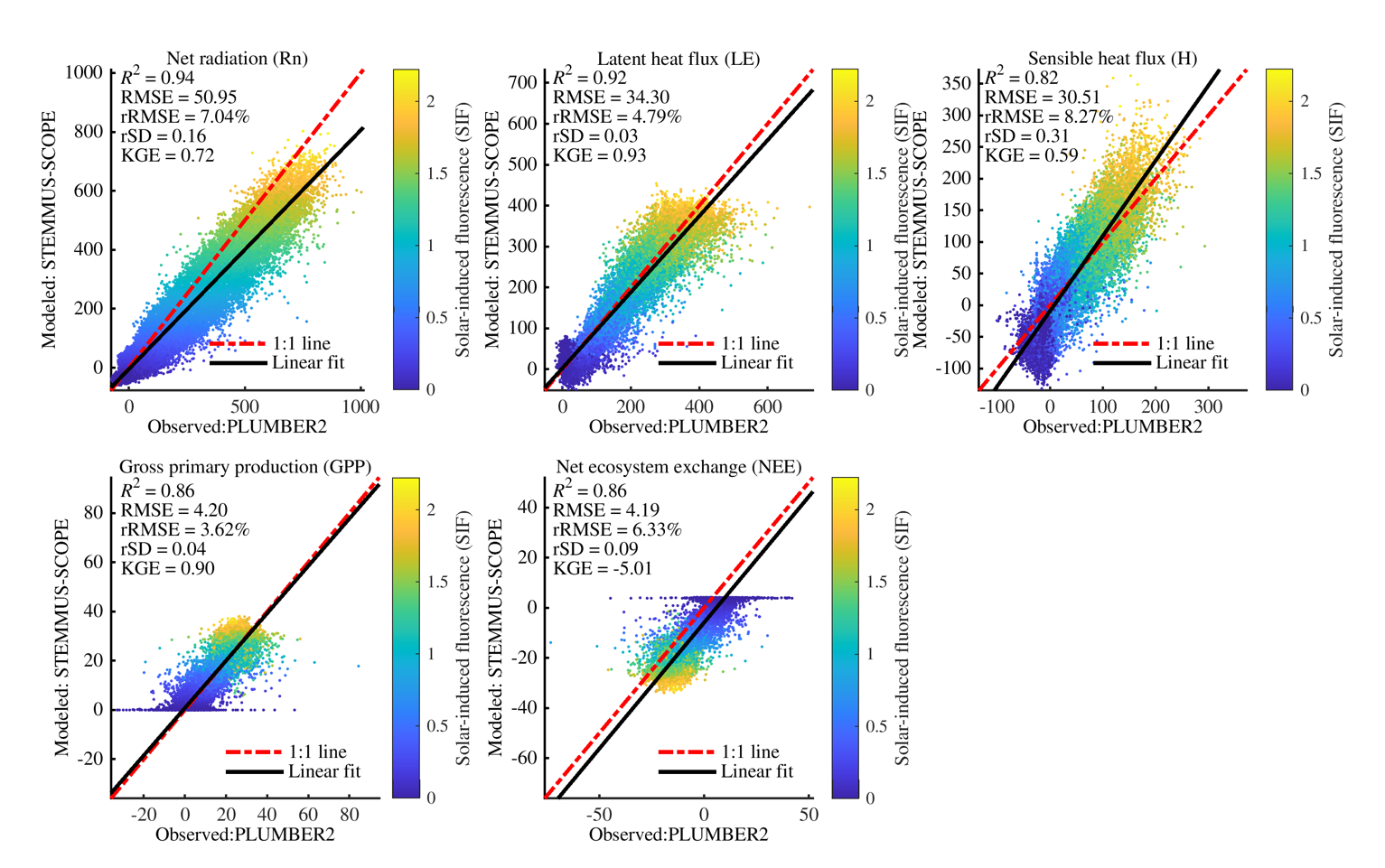


**Supplementary Figure 11: Correlation between modelled and observed energy and carbon fluxes at ID-Pag site, EBF**


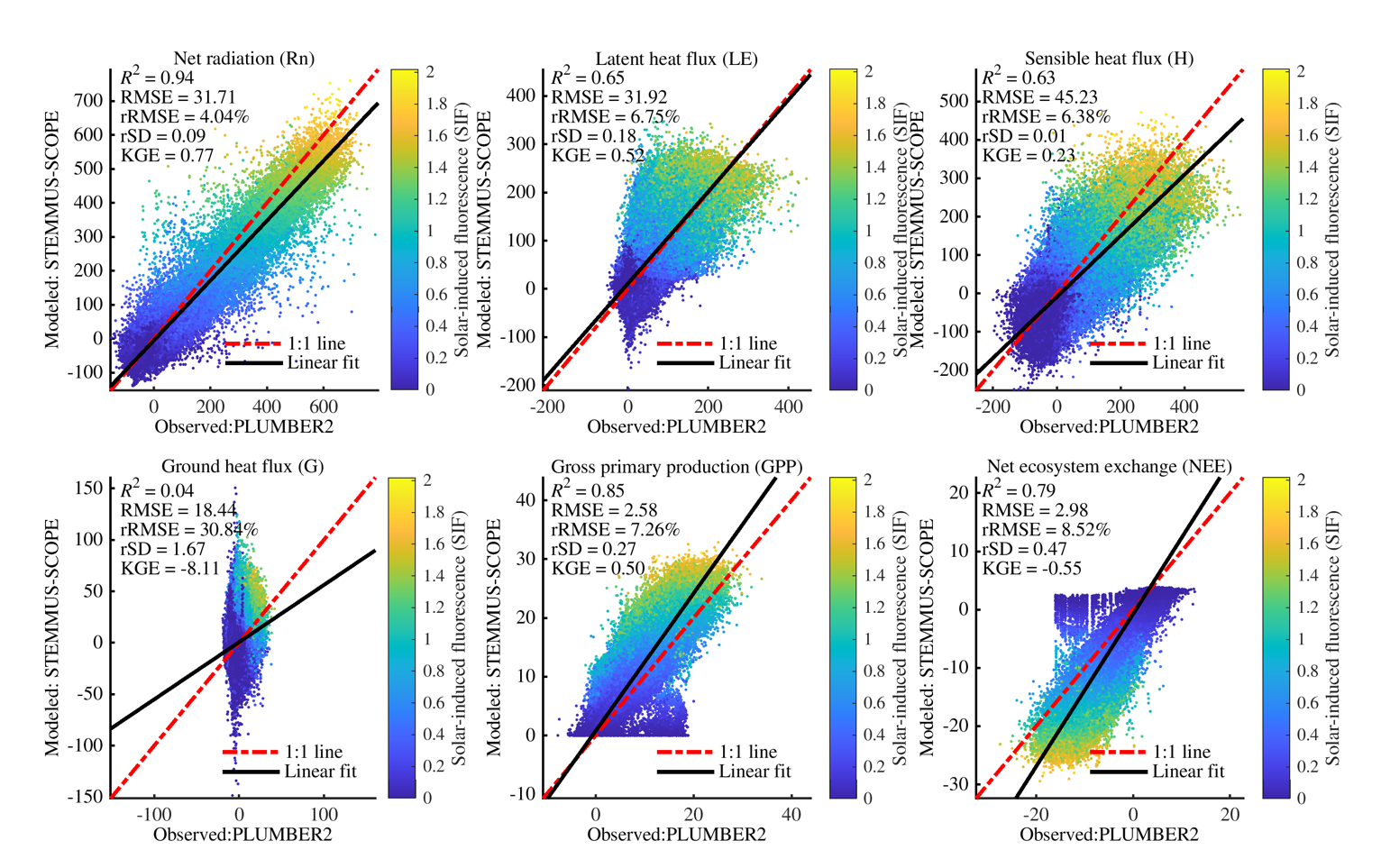


**Supplementary Figure 12: Correlation between modelled and observed energy and carbon fluxes at FI-Hyy site, ENF**


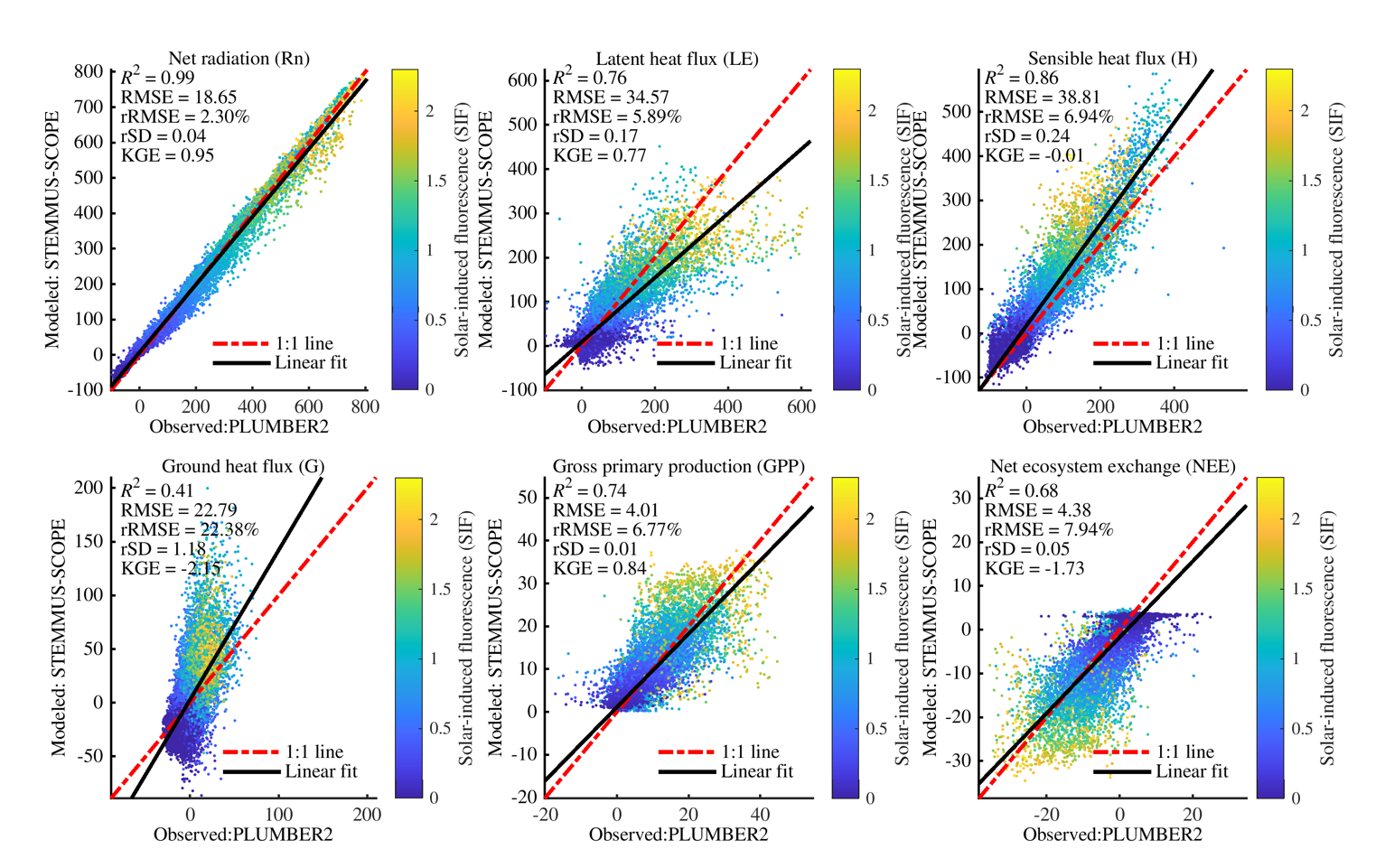


**Supplementary Figure 13: Correlation between modelled and observed energy and carbon fluxes at AU-Otw site, Grassland**


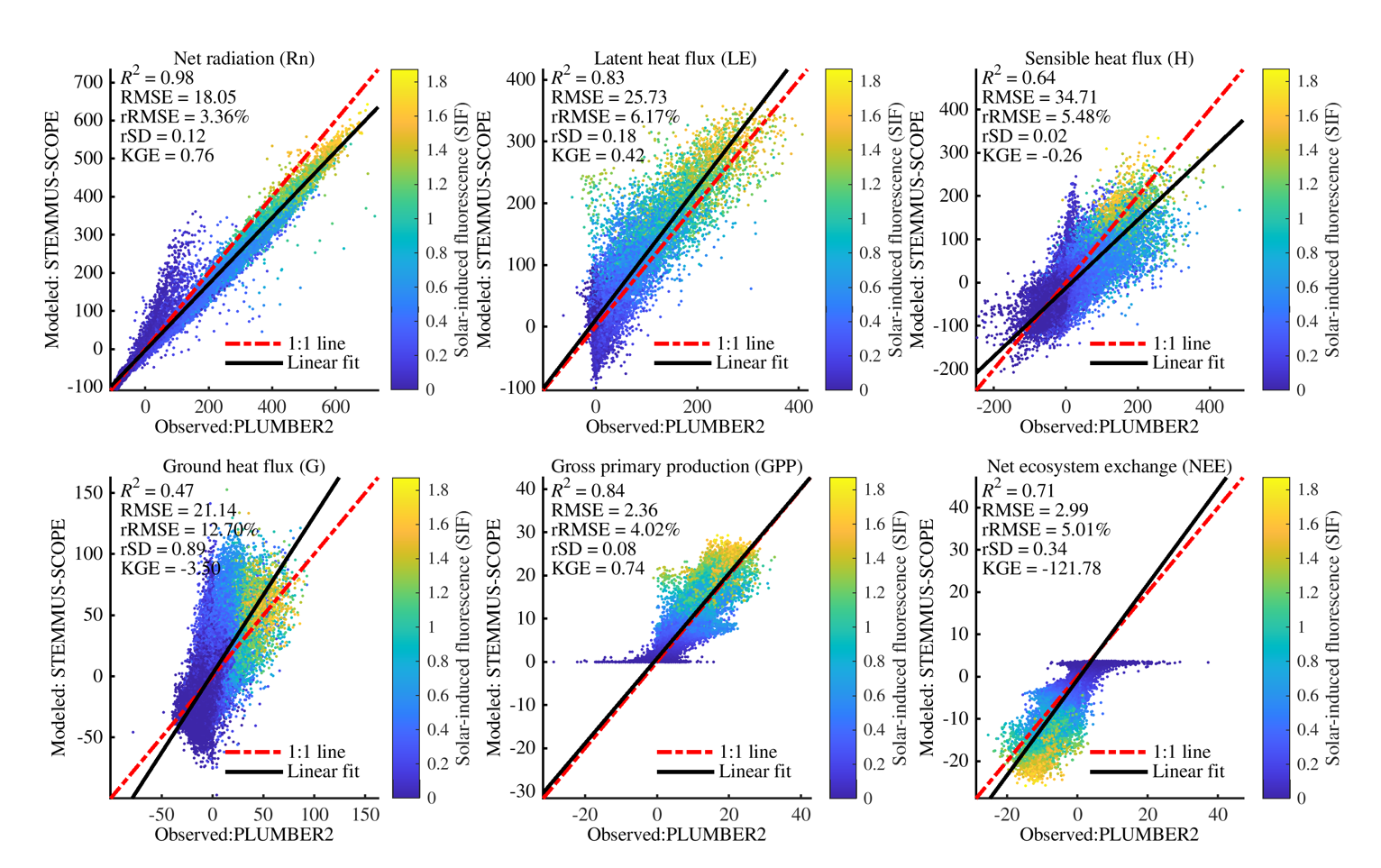


**Supplementary Figure 14: Correlation between modelled and observed energy and carbon fluxes at DE-Meh site, Mix forest**


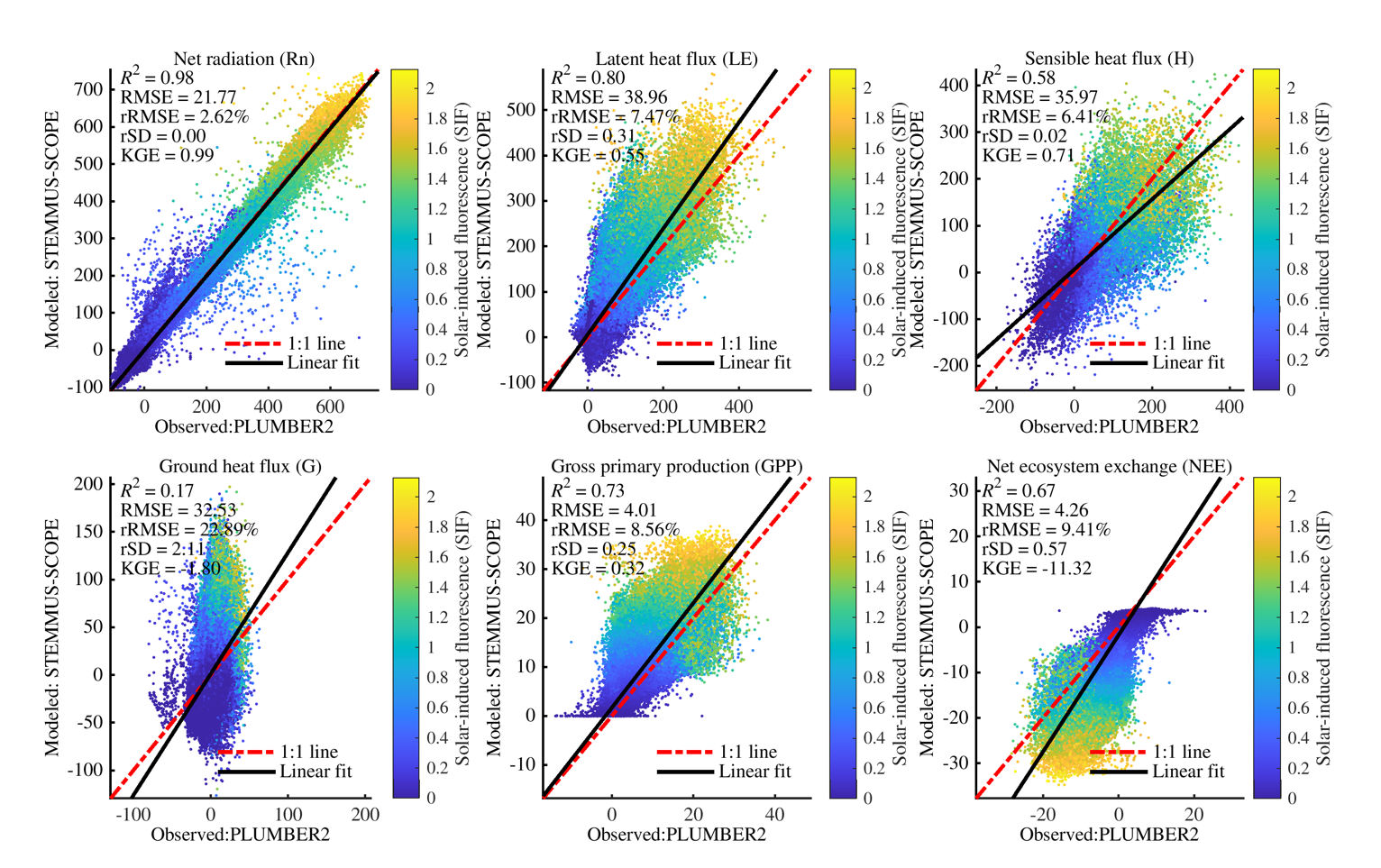


**Supplementary Figure 15: Correlation between modelled and observed energy and carbon fluxes at CZ-wet site, Wetland**


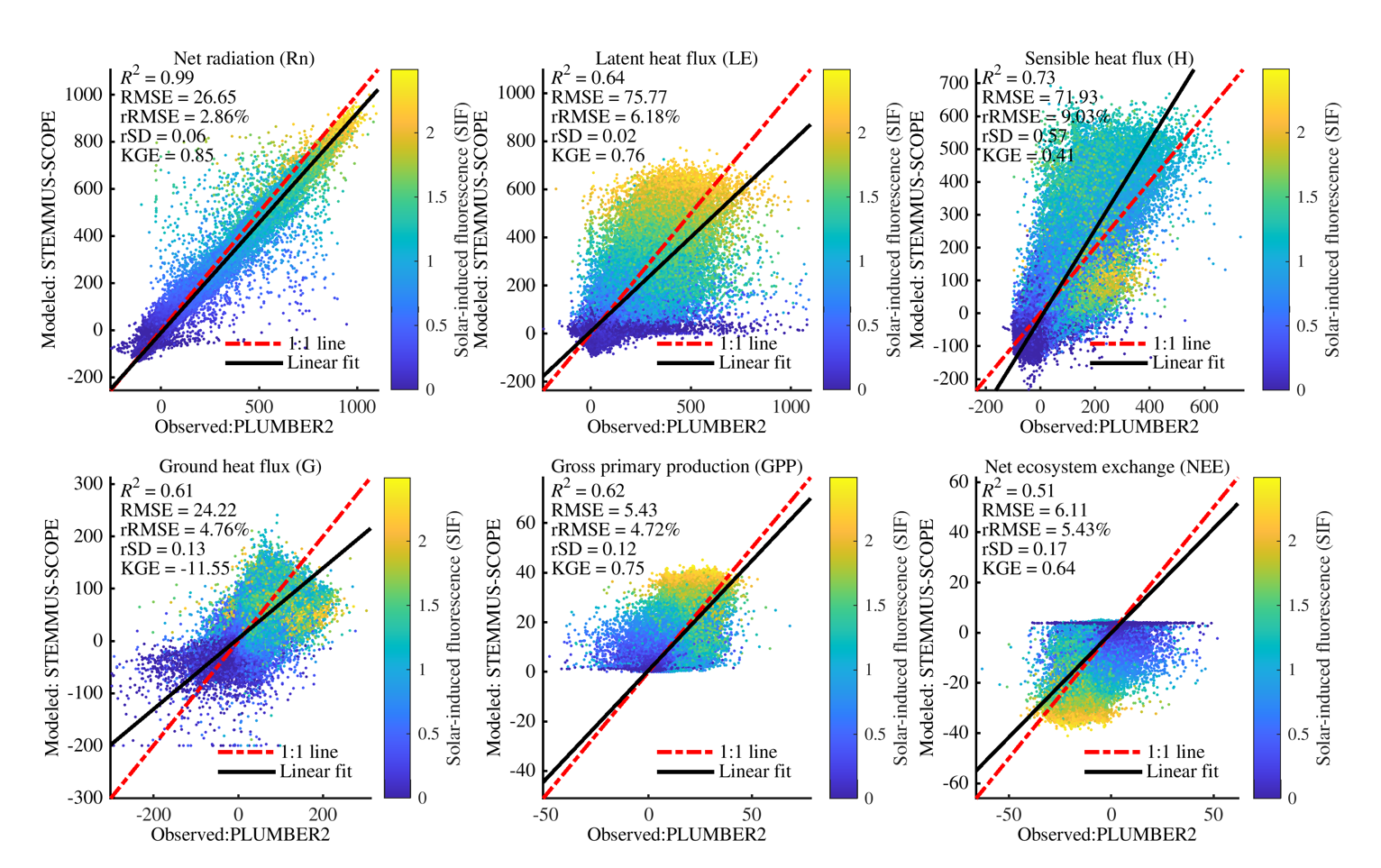


**Supplementary Figure 16: Correlation between modelled and observed energy and carbon fluxes at AU-How site, Savanna**


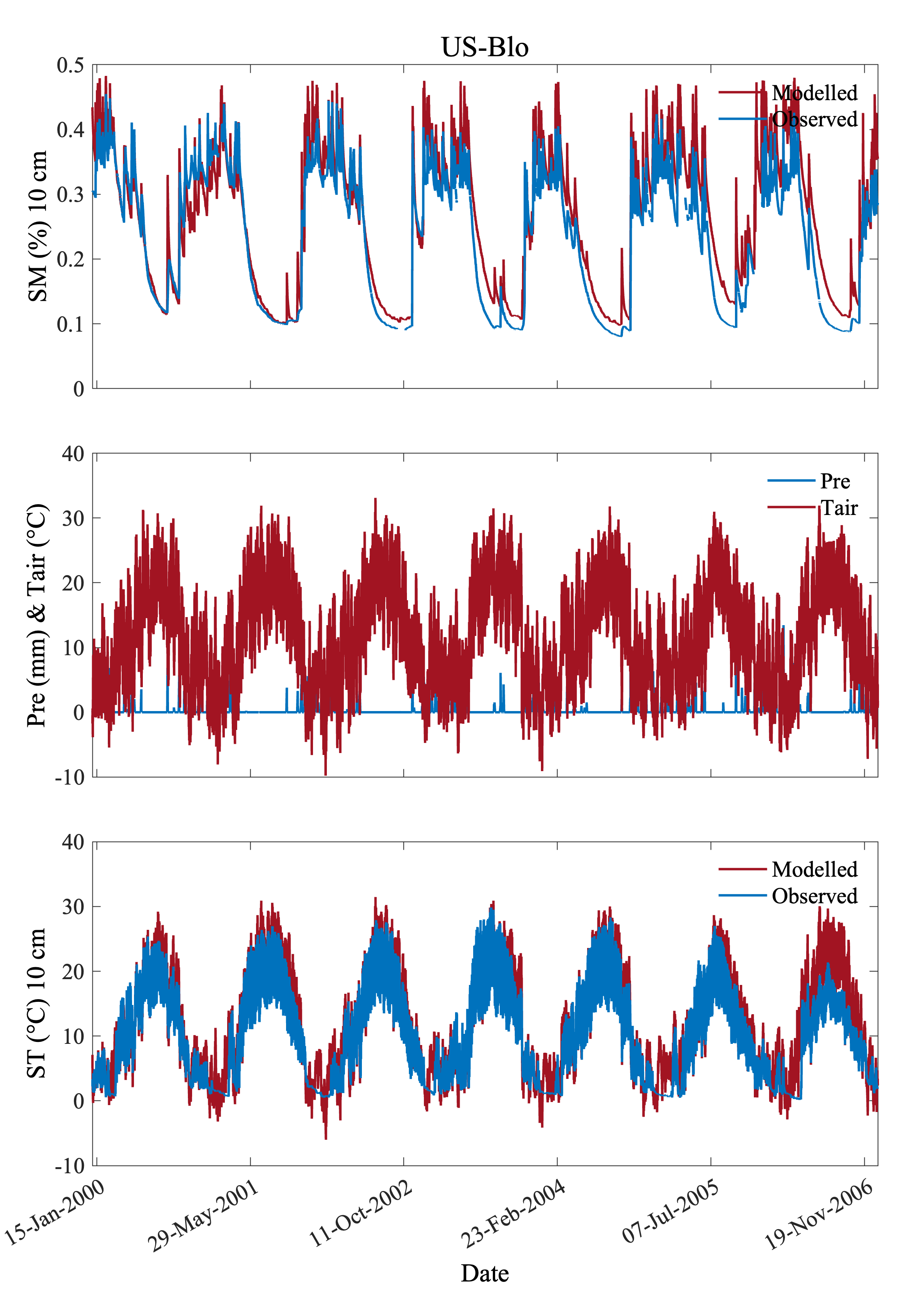


Supplementary Figure 17: Time series of modelled and observed soil moisture at US-Blo site (SM is volumetric soil moisture at 5cm depth; Pre and Tair are precipitation and air temperature; ST is soil temperature at 5cm depth).


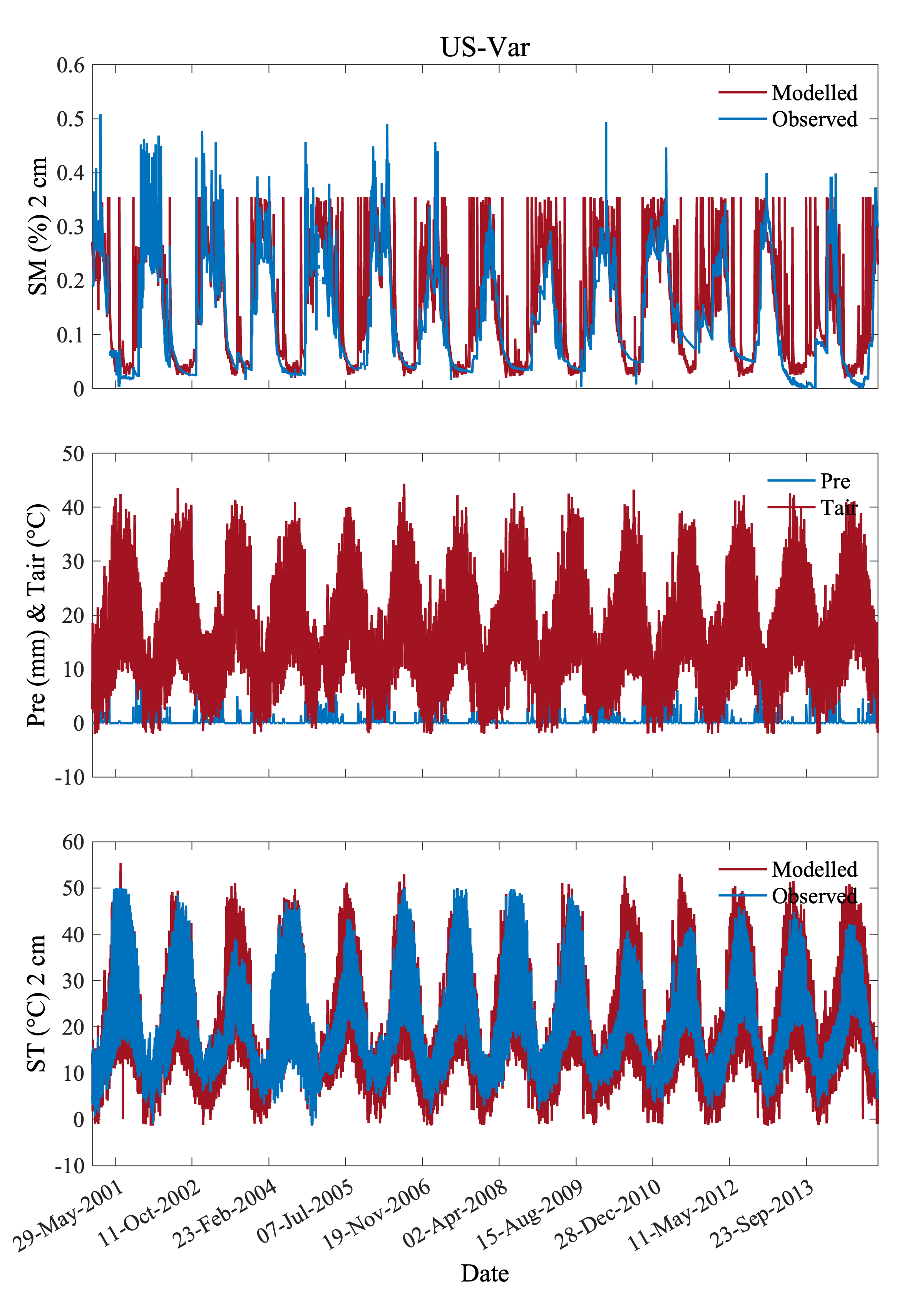


Supplementary Figure 18: Same as Supplementary Figure 17 but at US-Var site.


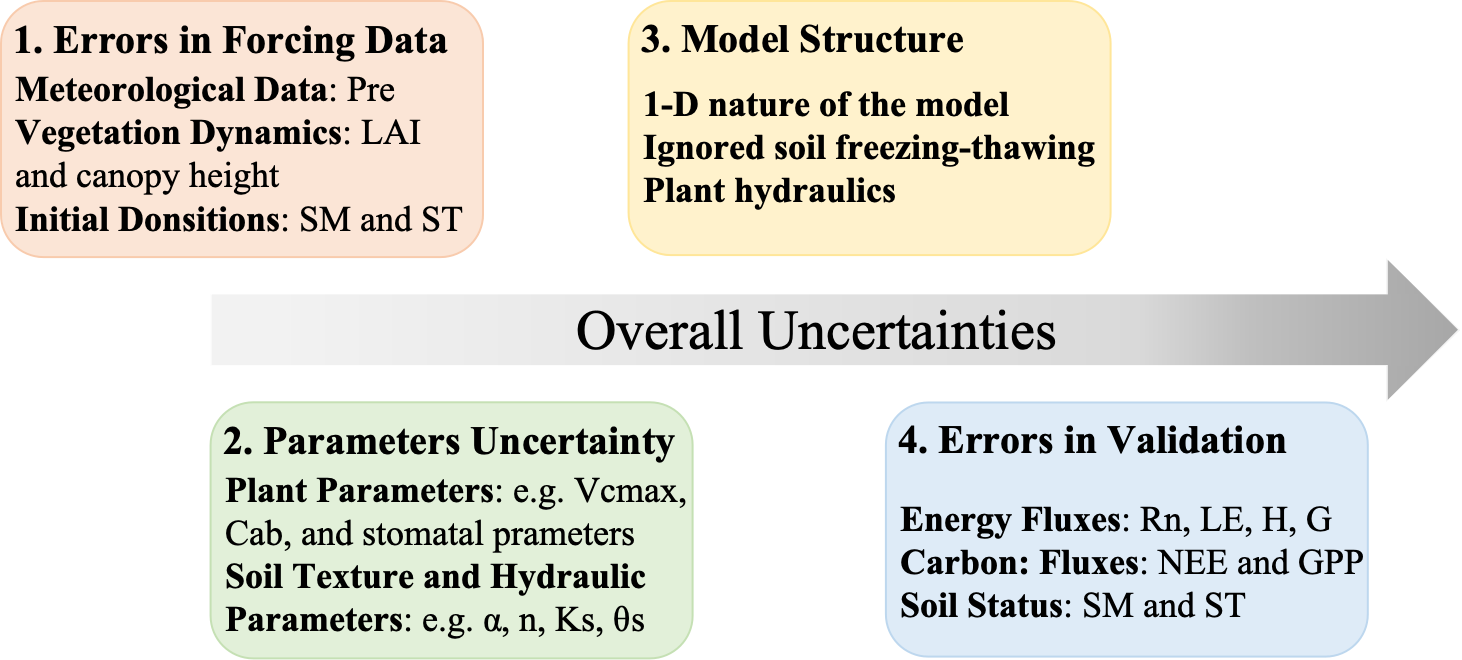


**Figure 19: Conceptual illustration of uncertainty sources in the simulations.**


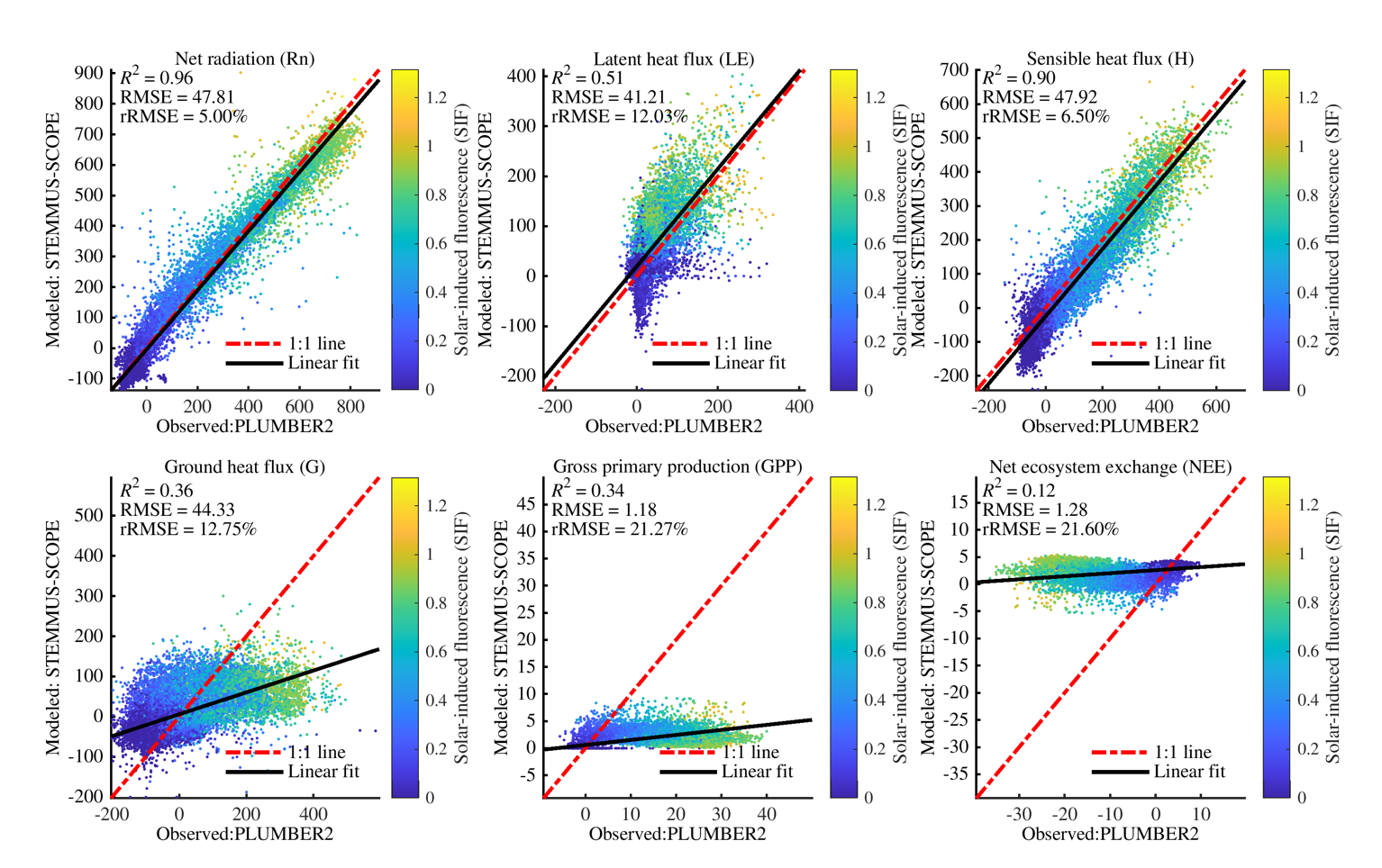


**Supplementary Figure 20. Energy and carbon fluxes simulations of AR-SLu (MODIS LAI).**


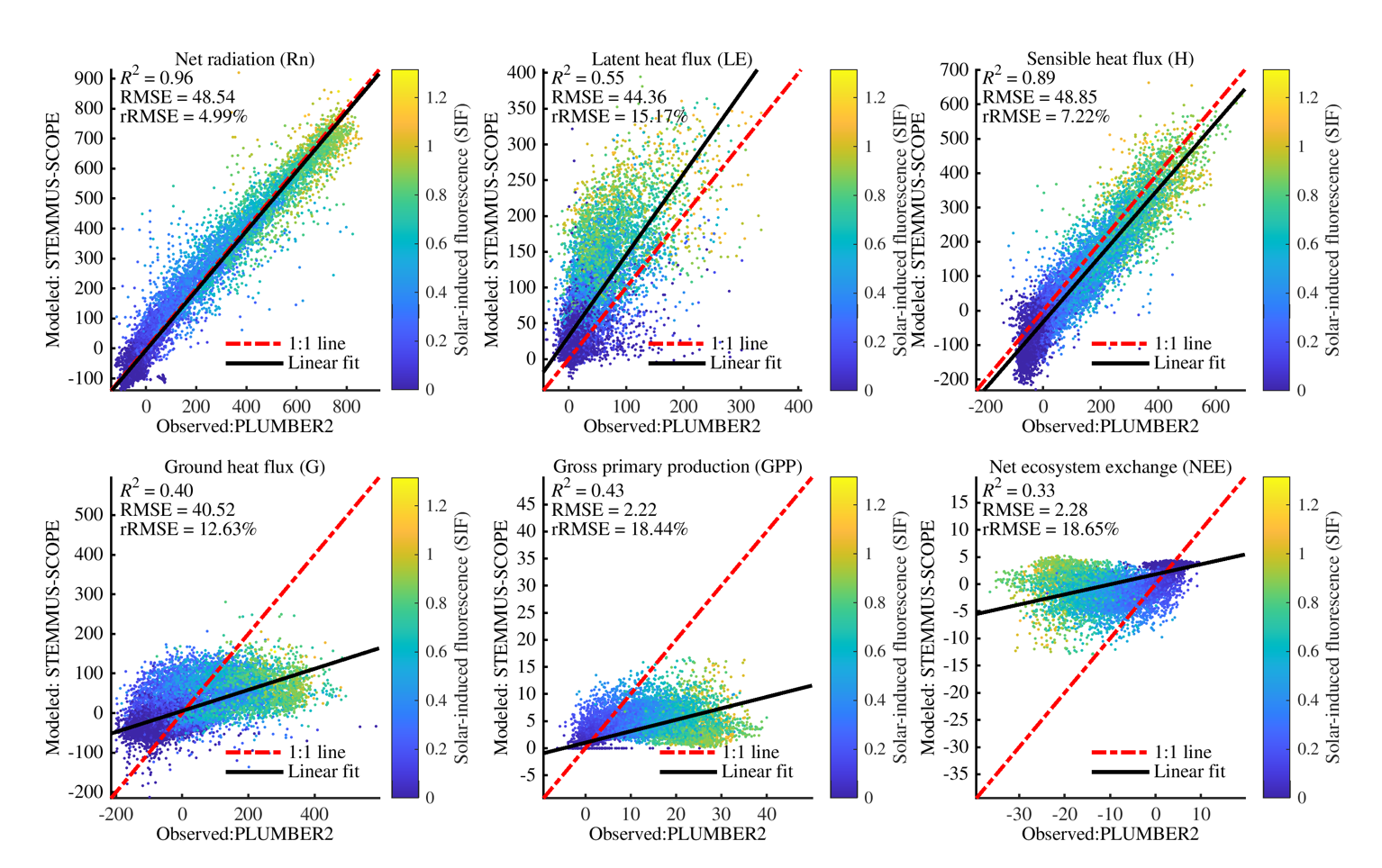


**Supplementary Figure 21. Energy and carbon fluxes simulations of AR-SLu (MODIS LAI * 2).**

**
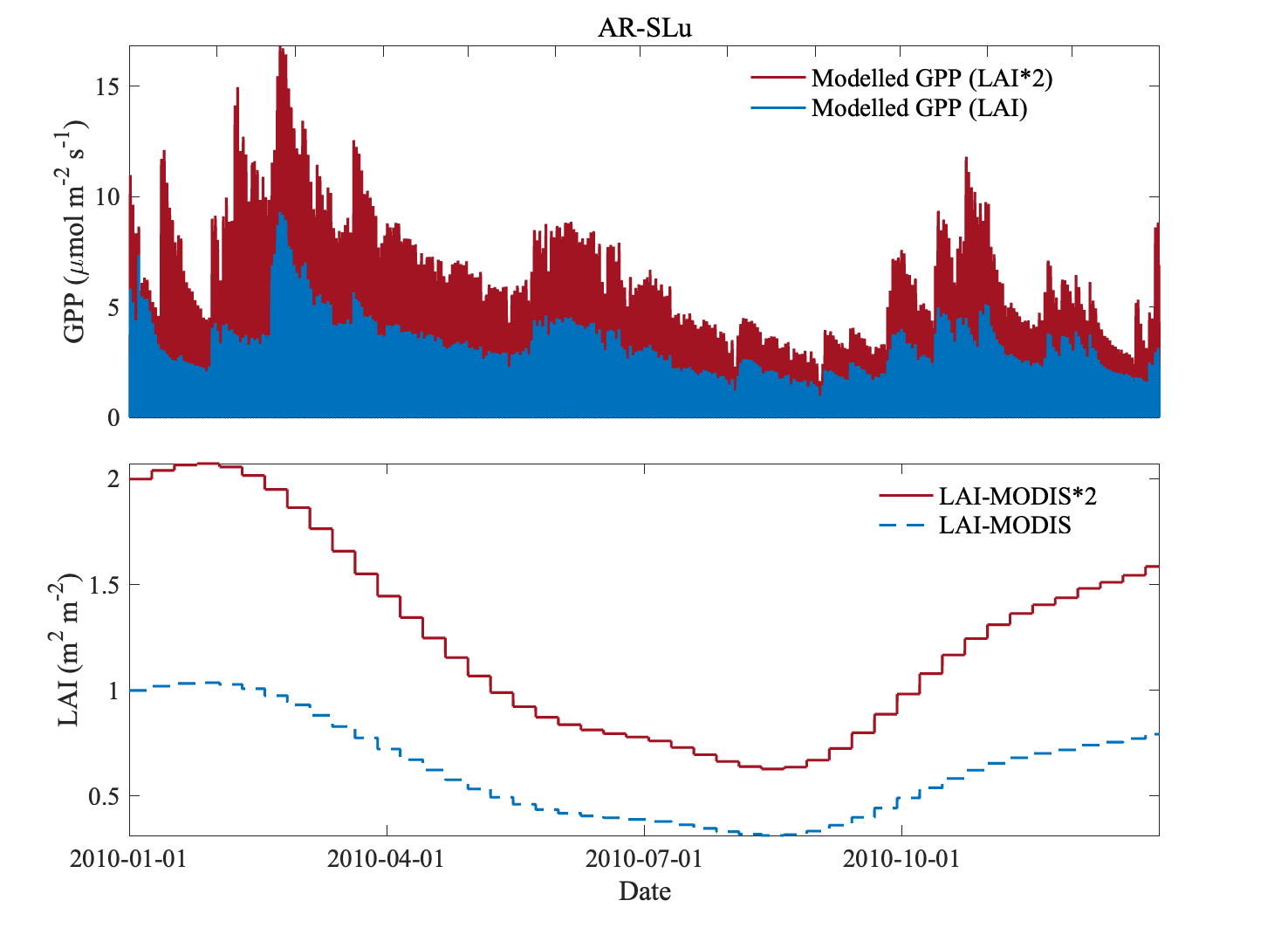
**

**Supplementary Figure 22. The modelled GPP with different LAI input at AR-SLu.**


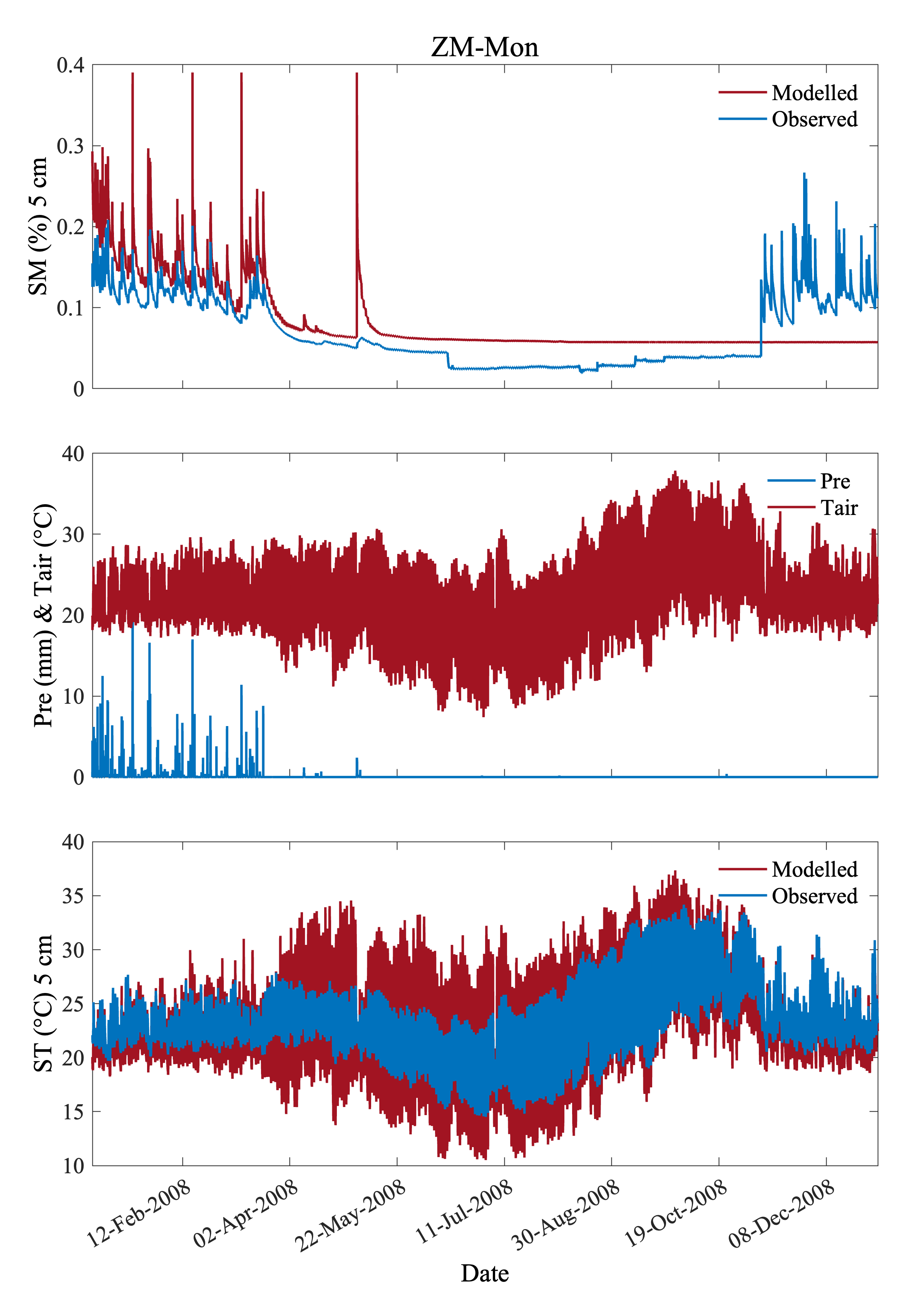


Supplementary Figure 23: Same as Supplementary Figure 17 but at ZM-Mon site.


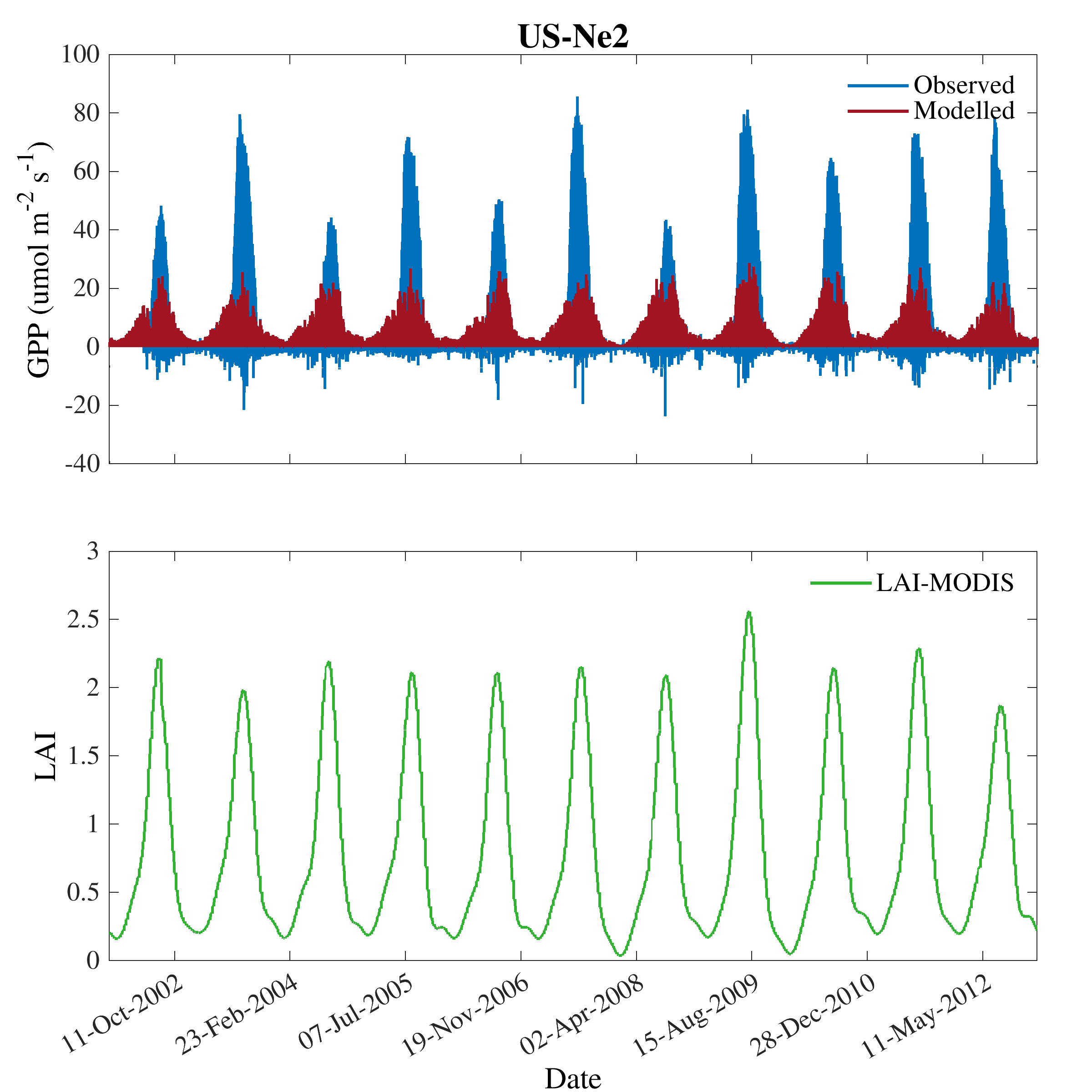


**Supplementary Figure 24. Observed and modeled GPP and remote sensing LAI of US-Ne2.**


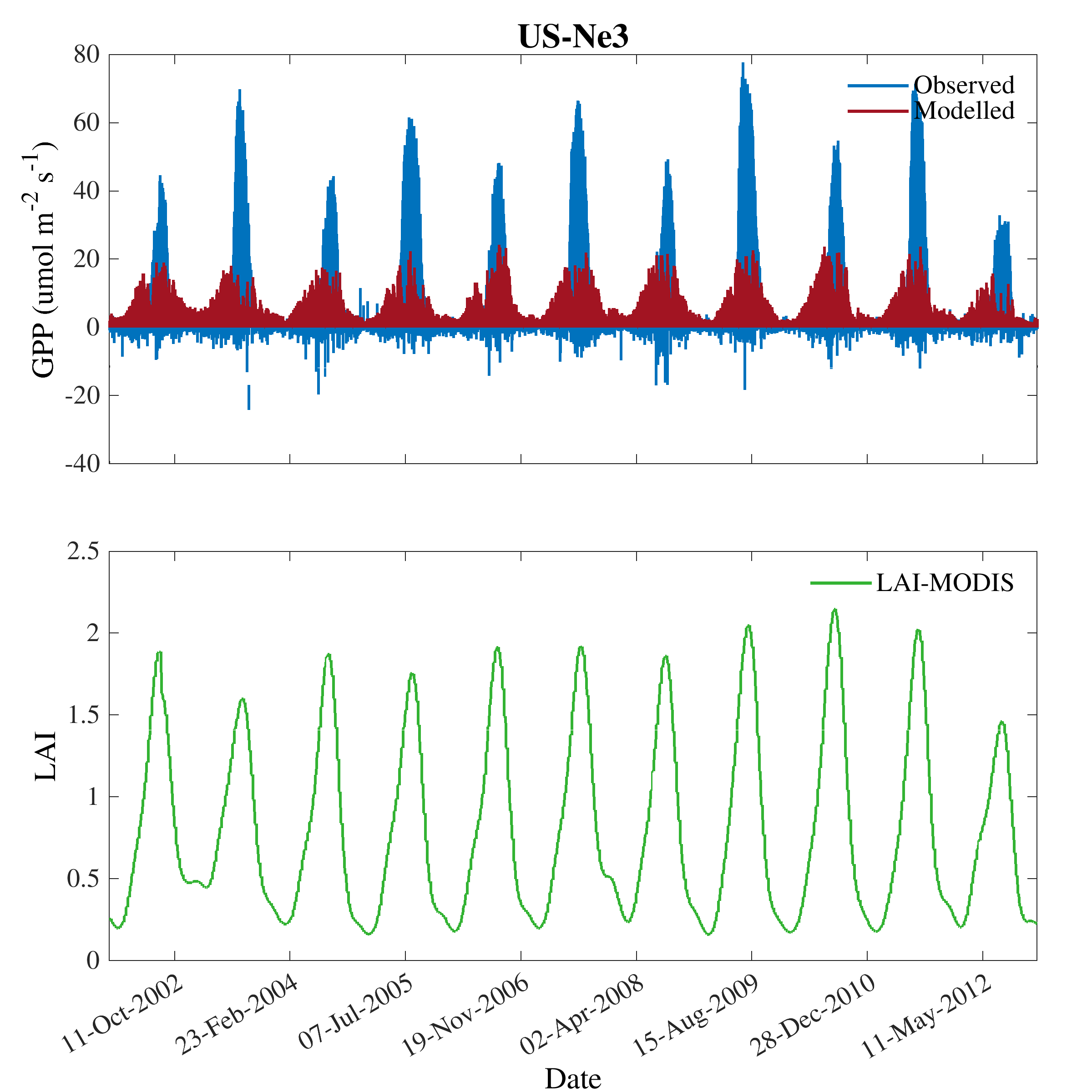


**Supplementary Figure 25. Observed and modeled GPP and remote sensing LAI of US-Ne3.**


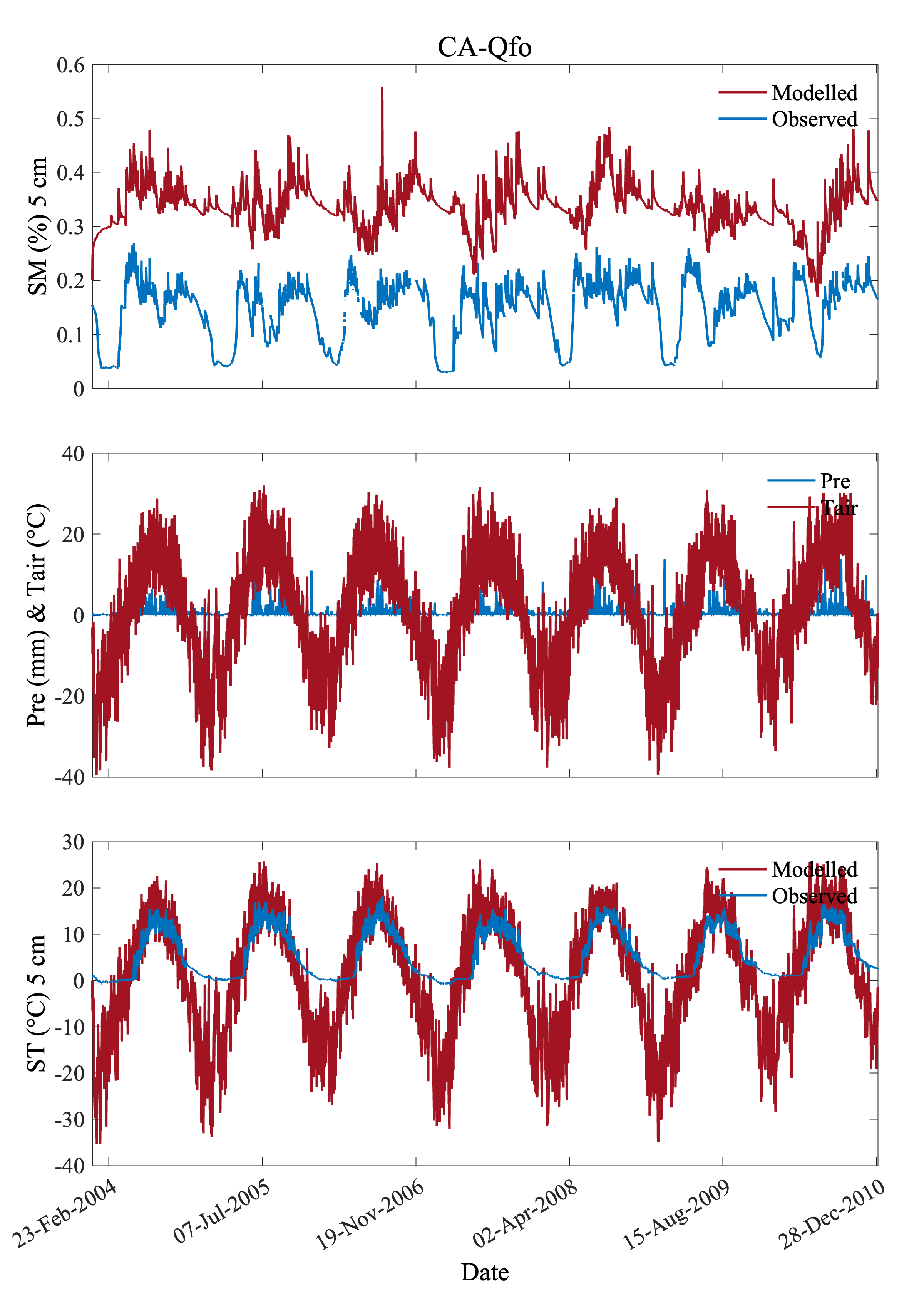


Supplementary Figure 26: Same as Supplementary Figure 17 but at CA-Qfo site.


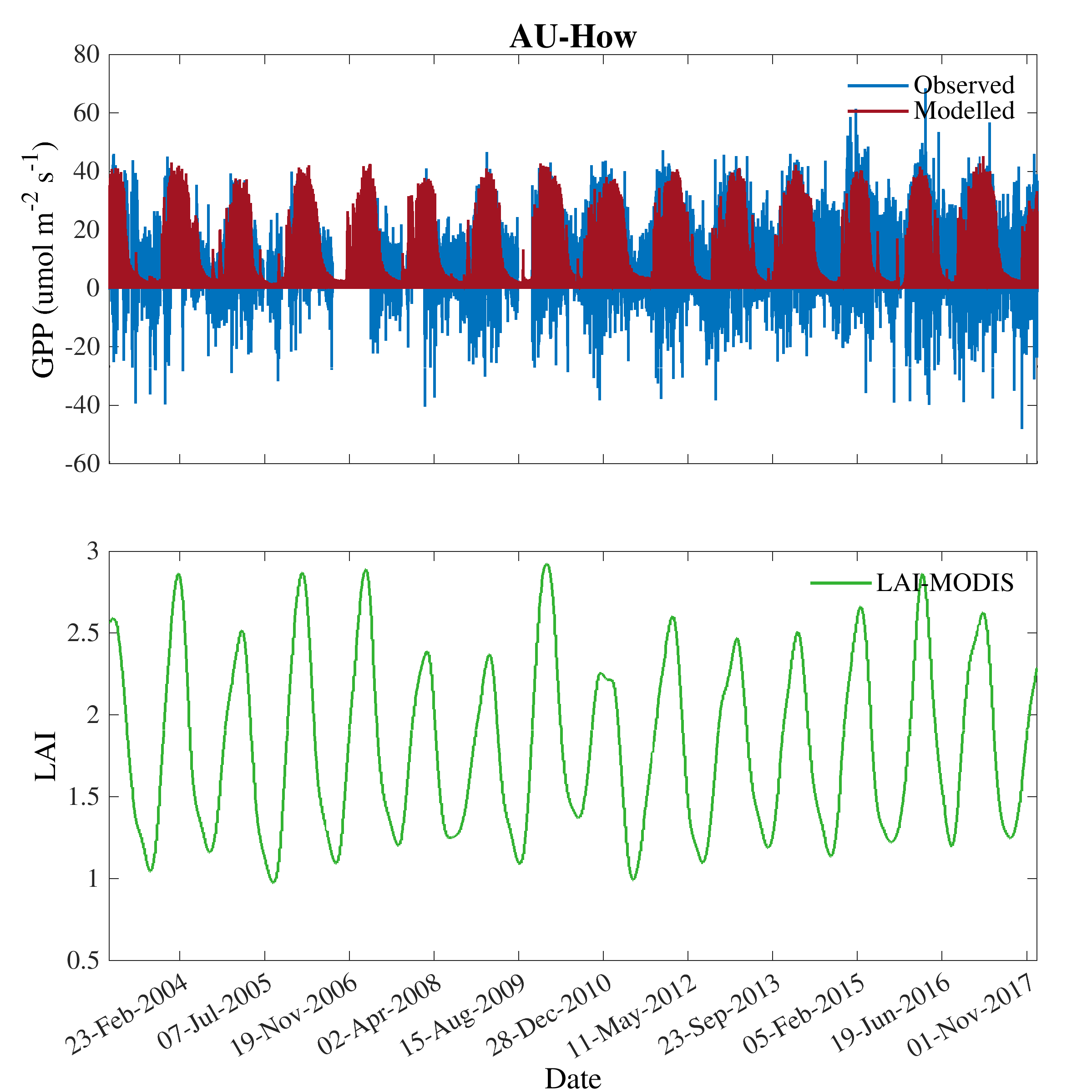


Supplementary Figure 27: Observed and modeled GPP and remote sensing LAI of AU-How.


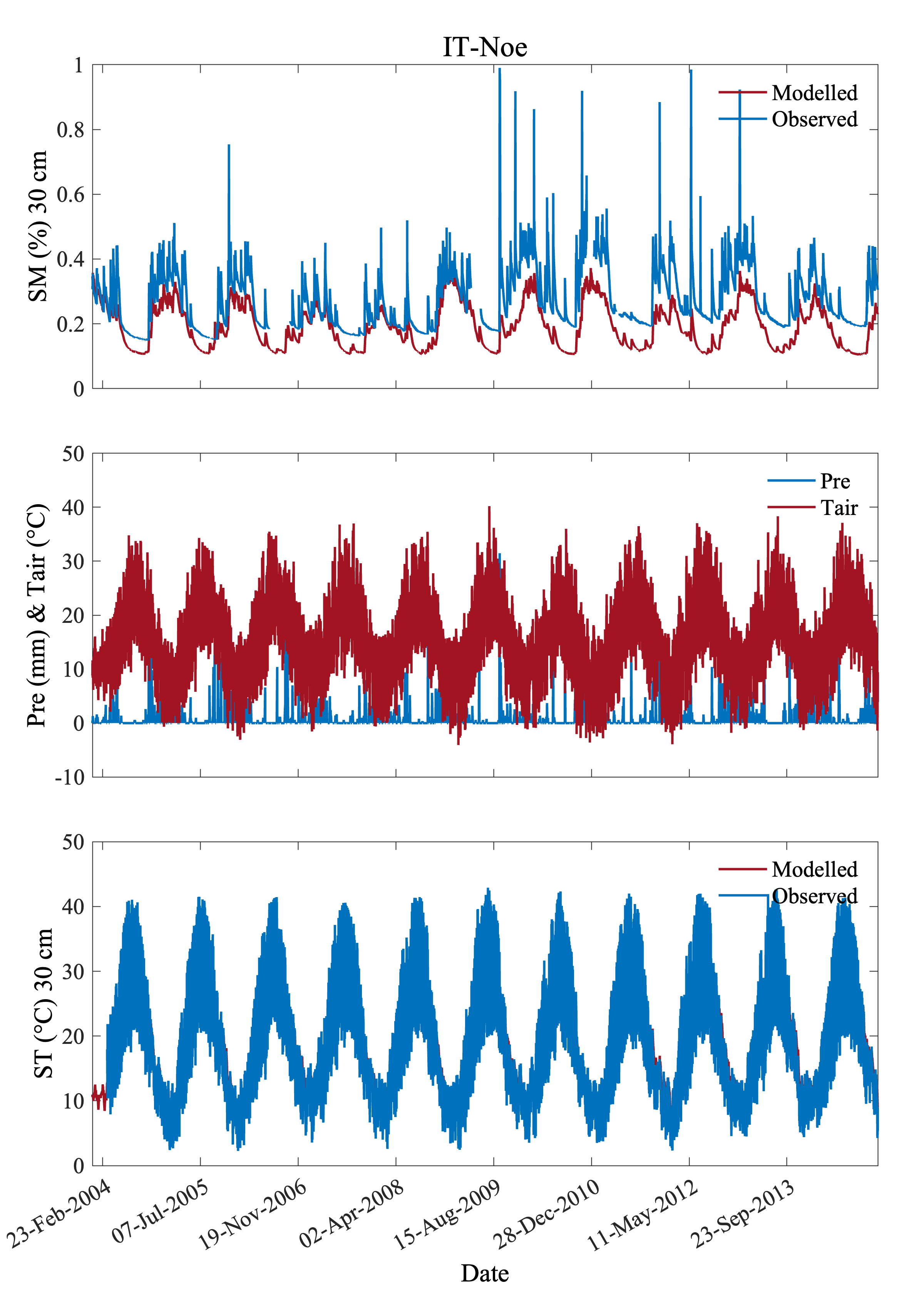


Supplementary Figure 28: Same as Supplementary Figure 17 but at IT-Noe site.

**Supplementary Table 1. The descriptions of the datasets used in this study.**

| **Variables** | **Datasets** | **Description and resolution** | **Download Link** | **References** |
| --- | --- | --- | --- | --- |
| **Meteorological and biological forcing** | PLUMBER2 | The second phase of the Protocol for the Analysis of Land Surface Models (PALS) Land Surface Model Benchmarking Evaluation Project (PLUMBER2) provides fully gap-filled meteorological data, reference measurement heights, vegetation types, and satellite-based monthly leaf area index (LAI) estimates. | http://doi.org/10.25914/5fdb0902607e1 | Ukkola, A. M., et al.: A flux tower dataset tailored for land model evaluation, Earth Syst. Sci. Data, 14, 449-461, 2022. |
| **Soil texture** | A Global Soil Data Set for Earth System Modeling (GSDE) | GSDE provides the vertical and horizontal variations of soil texture (sand, silt and clay contents, bulk density, and soil organic carbon). (0.0083° × 0.0083°) | http://globalchange.bnu.edu.cn/research/soilw | Shangguan, W., et al.: A global soil data set for earth system modeling, J. Adv. Model. Earth Sys., 6, 249-263, 2014. |
| **Soil hydraulic parameters** | SoilGridsTM | SoilGrids, which uses advanced machine learning methods to map the spatial distribution of soil properties across the globe, was used to provide the soil hydraulic parameters. (0.0083° × 0.0083°) | https://doi.org/10.1594/PANGAEA.870605 | Montzka, C., et al.: A global data set of soil hydraulic properties and sub-grid variability of soil water retention and hydraulic conductivity curves, Earth Syst. Sci. Data, 9, 529-543, 2017. |
| **Runoff** | SIMTOP | SIMTOP is a simple TOPMODEL-based surface runoff parameterization, which is consistent with that used in the Common Land Model. Maximum saturated fractional area (*F_max_*, %) is the key parameter in SIMTOP. (0.5° × 0.5°) |  | Niu, G.-Y., et al.: A simple TOPMODEL-based runoff parameterization (SIMTOP) for use in global climate models, J. Geophys. Res., 110, 2005. |
| **Initial conditions** | ERA5-Land | The SM and soil temperature (ST) of the ERA5-land dataset were used as the initial values at the start time of the simulation. (0.1° × 0.1°) | https://doi.org/10.24381/cds. | Muñoz-Sabater, J., et al.: ERA5-Land: a state-of-the-art global reanalysis dataset for land applications, Earth Syst. Sci. Data, 13, 4349-4383, 2021. |
| **Model validation: energy, water, and carbon fluxes; soil moisture and temperature** | PLUMBER2 and FLUXNET2015 | For model evaluation, the PLUMBER2 dataset provides key energy, water, and carbon fluxes. Because SM and ST were missing in PLUMBER2, we used FLUXNET2015 to validate the simulation of ST and SM of STEMMUS-SCOPE. | http://doi.org/10.25914/5fdb0902607e1  https://fluxnet.org/data/fluxnet2015-dataset/ | Ukkola, A. M., et al.: A flux tower dataset tailored for land model evaluation, Earth Syst. Sci. Data, 14, 449-461, 2022.  Pastorello, G., et al.: The FLUXNET2015 dataset and the ONEFlux processing pipeline for eddy covariance data, Sci. Data, 7, 225, 2020. |
| **Model validation: SIF** | OCO2 | Spatially contiguous global OCO-2 SIF product at 0.05°and 16-day resolutions was used to compared with the simulated SIF by STEMMUS-SCOPE. | https://doi.org/10.1029/2018GL081109 | Yu, L., et al.: High‐resolution global contiguous SIF of OCO‐2. Geophysical Research Letters, 46(3), 1449-1458, 2019. |
| **Model validation: Reflectance** | MODIS | The MOD09Q1 V6 product estimates Terra MODIS Bands 1 and 2 surface reflectance at 250 m resolution, corrected for atmospheric effects. It includes two quality layers, selecting a value per pixel from an 8-day composite. | https://lpdaac.usgs.gov/products/mod09q1v006/ | Vermote, E. F., et al.: Atmospheric correction algorithm: spectral reflectances (MOD09). ATBD version, 4, 1-107, 1999. |
| **Other parameters** | Default values in STEMMUS-SCOPE | According to the original SCOPE model, the canopy parameters such as maximum carboxylation capacity (Vcmax), Chlorophyll content (Cab), Ball-Berry stomatal conductance parameters (Ballberry0 and m), and dark respiration rate (Rd). were set with a lookup table. The plant-dependent root distribution parameter ($\beta$) was adopted from Jackson et al. (1996). |  | Van der Tol, C., et al.: An integrated model of soil-canopy spectral radiances, photosynthesis, fluorescence, temperature and energy balance, Biogeoscience, 6, 3109-3129, 2009.  Jackson, R. B., et al.: A global analysis of root distributions for terrestrial biomes, Oecologia, 108, 389-411, 10.1007/BF00333714, 1996. |

Supplementary Table 2. Detailed information of the 170 sites.

| Site_name | Country | Vege_type | Lat | Lon | Alt (m) | Pre (mm a^-1^) |
| --- | --- | --- | --- | --- | --- | --- |
| AR-SLu | Argentina | MF | -33.46 | -66.46 | 500 | 352 |
| AT-Neu | Austria | GRA | 47.12 | 11.32 | 970 | 668 |
| AU-ASM | Australia | ENF | -22.28 | 133.25 | 606 | 362 |
| AU-Cow | Australia | EBF | -16.24 | 145.43 | 86 | 4058 |
| AU-Cpr | Australia | SAV | -34.00 | 140.59 | 76 | 283 |
| AU-Ctr | Australia | EBF | -16.10 | 145.45 | 66 | 4227 |
| AU-Cum | Australia | EBF | -33.61 | 150.72 | 200 | 828 |
| AU-DaP | Australia | GRA | -14.06 | 131.32 | 116 | 1367 |
| AU-DaS | Australia | SAV | -14.16 | 131.39 | 108 | 1355 |
| AU-Dry | Australia | SAV | -15.26 | 132.37 | 191 | 977 |
| AU-Emr | Australia | GRA | -23.86 | 148.47 | 177 | 626 |
| AU-GWW | Australia | SAV | -30.19 | 120.65 | 504 | 304 |
| AU-Gin | Australia | SAV | -31.38 | 115.65 | 51 | 625 |
| AU-How | Australia | SAV | -12.50 | 131.15 | 41 | 1648 |
| AU-Lit | Australia | SAV | -13.18 | 130.79 | 200 | 1561 |
| AU-Otw | Australia | GRA | -38.53 | 142.82 | 54 | 784 |
| AU-Rig | Australia | GRA | -36.65 | 145.58 | 133 | 488 |
| AU-Rob | Australia | EBF | -17.12 | 145.63 | 710 |  |
| AU-Sam | Australia | GRA | -27.39 | 152.88 | 170 | 1253 |
| AU-Stp | Australia | GRA | -17.15 | 133.35 | 252 | 731 |
| AU-TTE | Australia | SHR | -22.29 | 133.64 | 553 | 387 |
| AU-Tum | Australia | EBF | -35.66 | 148.15 | 1200 | 1014 |
| AU-Whr | Australia | EBF | -36.67 | 145.03 | 152 | 409 |
| AU-Wrr | Australia | EBF | -43.10 | 146.65 | 100 | 1338 |
| AU-Ync | Australia | GRA | -34.99 | 146.29 | 125 | 362 |
| BE-Bra | Belgium | MF | 51.31 | 4.52 | 16 | 776 |
| BE-Lon | Belgium | CRO | 50.55 | 4.75 | 167 | 768 |
| BE-Vie | Belgium | MF | 50.31 | 6.00 | 493 | 964 |
| BR-Sa3 | Brazil | EBF | -3.02 | -54.97 | 100 | 1408 |
| BW-Ma1 | Botswana | SAV | -19.92 | 23.56 | 929 | 428 |
| CA-NS1 | Canada | ENF | 55.88 | -98.48 | 260 | 208 |
| CA-NS2 | Canada | ENF | 55.91 | -98.52 | 260 | 250 |
| CA-NS4 | Canada | ENF | 55.91 | -98.38 | 260 | 217 |
| CA-NS5 | Canada | ENF | 55.86 | -98.49 | 260 | 182 |
| CA-NS6 | Canada | SHR | 55.92 | -98.96 | 244 | 202 |
| CA-NS7 | Canada | SHR | 56.64 | -99.95 | 297 | 235 |
| CA-Qcu | Canada | ENF | 49.27 | -74.04 | 392 | 928 |
| CA-Qfo | Canada | ENF | 49.69 | -74.34 | 382 | 938 |
| CA-SF1 | Canada | ENF | 54.49 | -105.82 | 536 | 479 |
| CA-SF2 | Canada | ENF | 54.25 | -105.88 | 520 | 336 |
| CA-SF3 | Canada | SHR | 54.09 | -106.01 | 540 | 390 |
| CH-Cha | Switzerland | GRA | 47.21 | 8.41 | 393 | 1143 |
| CH-Dav | Switzerland | ENF | 46.82 | 9.86 | 1639 | 841 |
| CH-Fru | Switzerland | GRA | 47.12 | 8.54 | 982 | 1291 |
| CH-Oe1 | Switzerland | GRA | 47.29 | 7.73 | 450 | 1221 |
| CN-Cha | China | MF | 42.40 | 128.10 | 754 | 466 |
| CN-Cng | China | GRA | 44.59 | 123.51 | 138 | 332 |
| CN-Dan | China | GRA | 30.50 | 91.07 | 4751 | 519 |
| CN-Din | China | EBF | 23.17 | 112.54 | 261 | 1372 |
| CN-Du2 | China | GRA | 42.05 | 116.28 | 1331 | 285 |
| CN-HaM | China | GRA | 37.37 | 101.18 | 3975 | 583 |
| CN-Qia | China | ENF | 26.74 | 115.06 | 64 | 1169 |
| CZ-wet | Czech Republic | WET | 49.02 | 14.77 | 426 | 609 |
| DE-Bay | Germany | ENF | 50.14 | 11.87 | 781 | 1019 |
| DE-Geb | Germany | CRO | 51.10 | 10.91 | 161.5 | 531 |
| DE-Gri | Germany | GRA | 50.95 | 13.51 | 385 | 942 |
| DE-Hai | Germany | DBF | 51.08 | 10.45 | 430 | 761 |
| DE-Kli | Germany | CRO | 50.89 | 13.52 | 478 | 817 |
| DE-Meh | Germany | MF | 51.28 | 10.66 | 291 | 520 |
| DE-Obe | Germany | ENF | 50.79 | 13.72 | 734 | 1046 |
| DE-Seh | Germany | CRO | 50.87 | 6.45 | 103 | 527 |
| DE-SfN | Germany | WET | 47.81 | 11.33 | 590 | 888 |
| DE-Tha | Germany | ENF | 50.96 | 13.57 | 380 | 851 |
| DE-Wet | Germany | ENF | 50.45 | 11.46 | 703 | 1021 |
| DK-Fou | Germany | CRO | 56.48 | 9.59 | 51 | 643 |
| DK-Lva | Germany | GRA | 55.68 | 12.08 | -2493 | 1001 |
| DK-Ris | Germany | CRO | 55.53 | 12.10 | 24 | 481 |
| DK-Sor | Germany | DBF | 55.49 | 11.64 | 40 | 870 |
| DK-ZaH | Germany | GRA | 74.47 | -20.55 | 38 | 147 |
| ES-ES1 | Spain | ENF | 39.35 | -0.32 | 1 | 559 |
| ES-ES2 | Spain | CRO | 39.28 | -0.32 | 7 | 578 |
| ES-LMa | Spain | SAV | 39.94 | -5.77 | 278 | 691 |
| ES-LgS | Spain | SHR | 37.10 | -2.97 | 2267 | 402 |
| ES-VDA | Spain | GRA | 42.15 | 1.45 | 1787 | 924 |
| FI-Hyy | Finland | ENF | 61.85 | 24.30 | 181 | 604 |
| FI-Kaa | Finland | WET | 69.14 | 27.30 | 159 | 485 |
| FI-Lom | Finland | WET | 68.00 | 24.21 | 274 | 535 |
| FI-Sod | Finland | ENF | 67.36 | 26.64 | 180 | 541 |
| FR-Fon | France | DBF | 48.48 | 2.78 | 103 | 671 |
| FR-Gri | France | CRO | 48.84 | 1.95 | 125 | 588 |
| FR-Hes | France | DBF | 48.67 | 7.07 | 293 | 960 |
| FR-LBr | France | ENF | 44.72 | -0.77 | 61 | 962 |
| FR-Lq1 | France | GRA | 45.64 | 2.74 | 1066 | 975 |
| FR-Lq2 | France | GRA | 45.64 | 2.74 | 1081 | 975 |
| FR-Pue | France | EBF | 43.74 | 3.60 | 270 | 921 |
| GF-Guy | French Guiana | EBF | 5.28 | -52.92 | 48 | 3108 |
| HU-Bug | Hungary | GRA | 46.69 | 19.60 | 106 | 530 |
| ID-Pag | Indonesia | EBF | -2.32 | 113.90 | 30 | 2072 |
| IE-Ca1 | Ireland | CRO | 52.86 | -6.92 | 72 | 679 |
| IE-Dri | Ireland | GRA | 51.99 | -8.75 | 186 | 1271 |
| IT-Amp | Italy | GRA | 41.90 | 13.61 | 991 | 853 |
| IT-BCi | Italy | CRO | 40.52 | 14.96 | 20 | 1228 |
| IT-CA1 | Italy | DBF | 42.38 | 12.03 | 200 | 719 |
| IT-CA2 | Italy | CRO | 42.38 | 12.03 | 200 | 720 |
| IT-CA3 | Italy | DBF | 42.38 | 12.02 | 197 | 708 |
| IT-Col | Italy | DBF | 41.85 | 13.59 | 1560 | 1169 |
| IT-Cpz | Italy | EBF | 41.71 | 12.38 | 68 | 820 |
| IT-Isp | Italy | DBF | 45.81 | 8.63 | 210 | 2001 |
| IT-LMa | Italy | DBF | 45.15 | 7.58 | 350 | 856 |
| IT-Lav | Italy | ENF | 45.96 | 11.28 | 1353 | 1291 |
| IT-MBo | Italy | GRA | 46.01 | 11.05 | 1550 | 969 |
| IT-Mal | Italy | GRA | 46.11 | 11.70 | 1610 | 1833 |
| IT-Noe | Italy | SHR | 40.61 | 8.15 | 25 | 570 |
| IT-Non | Italy | DBF | 44.69 | 11.09 | 14 | 1475 |
| IT-PT1 | Italy | DBF | 45.20 | 9.06 | 60 | 642 |
| IT-Ren | Italy | ENF | 46.59 | 11.43 | 1730 | 952 |
| IT-Ro1 | Italy | DBF | 42.41 | 11.93 | 235 | 860 |
| IT-Ro2 | Italy | DBF | 42.39 | 11.92 | 160 | 855 |
| IT-SR2 | Italy | ENF | 43.73 | 10.29 | 4 | 1329 |
| IT-SRo | Italy | ENF | 43.73 | 10.28 | 6 | 837 |
| JP-SMF | Japan | MF | 35.26 | 137.08 | 199 | 1579 |
| NL-Ca1 | Netherlands | GRA | 51.97 | 4.93 | 1 | 688 |
| NL-Hor | Netherlands | GRA | 52.24 | 5.07 | 2 | 986 |
| NL-Loo | Netherlands | ENF | 52.17 | 5.74 | 25 | 849 |
| PL-wet | Poland | WET | 52.76 | 16.31 | 55 | 519 |
| PT-Esp | Portugal | EBF | 38.64 | -8.60 | 90 | 611 |
| PT-Mi1 | Portugal | EBF | 38.54 | -8.00 | 230 | 244 |
| PT-Mi2 | Portugal | GRA | 38.48 | -8.02 | 193 | 633 |
| RU-Che | Russia | WET | 68.61 | 161.34 | 6 | 73 |
| RU-Fyo | Russia | ENF | 56.46 | 32.92 | 265 | 566 |
| RU-Zot | Russia | SAV | 60.80 | 89.35 | 124 | 251 |
| SD-Dem | Sudan | SAV | 13.28 | 30.48 | 500 | 285 |
| SE-Deg | Sweden | GRA | 64.18 | 19.56 | 270 | 456 |
| UK-Gri | United Kingdom | ENF | 56.61 | -3.80 | 343 | 1213 |
| UK-Ham | United Kingdom | DBF | 51.15 | -0.86 | 76 | 795 |
| UK-PL3 | United Kingdom | MF | 51.45 | -1.27 | 104 | 556 |
| US-AR1 | United States | GRA | 36.43 | -99.42 | 611 | 514 |
| US-AR2 | United States | GRA | 36.64 | -99.60 | 646 | 430 |
| US-ARM | United States | CRO | 36.61 | -97.49 | 314 | 645 |
| US-Aud | United States | GRA | 31.59 | -110.51 | 1466 | 337 |
| US-Bar | United States | MF | 44.06 | -71.29 | 270 | 1444 |
| US-Bkg | United States | GRA | 44.35 | -96.84 | 495 | 591 |
| US-Blo | United States | ENF | 38.90 | -120.63 | 1315 | 1400 |
| US-Bo1 | United States | CRO | 40.01 | -88.29 | 217 | 800 |
| US-Cop | United States | GRA | 38.09 | -109.39 | 1520 | 154 |
| US-FPe | United States | GRA | 48.31 | -105.10 | 638 | 394 |
| US-GLE | United States | ENF | 41.37 | -106.24 | 3197 | 1467 |
| US-Goo | United States | GRA | 34.25 | -89.87 | 87 | 1418 |
| US-Ha1 | United States | DBF | 42.54 | -72.17 | 340 | 1185 |
| US-Ho1 | United States | ENF | 45.20 | -68.74 | 60 | 817 |
| US-KS2 | United States | SHR | 28.61 | -80.67 | 3 | 1146 |
| US-Los | United States | WET | 46.08 | -89.98 | 480 | 765 |
| US-MMS | United States | DBF | 39.32 | -86.41 | 275 | 1083 |
| US-MOz | United States | DBF | 38.74 | -92.20 | 212 | 878 |
| US-Me2 | United States | ENF | 44.45 | -121.56 | 1253 | 486 |
| US-Me4 | United States | ENF | 44.50 | -121.62 | 922 | 641 |
| US-Me6 | United States | ENF | 44.32 | -121.61 | 998 | 416 |
| US-Myb | United States | WET | 38.05 | -121.77 | -1 | 335 |
| US-NR1 | United States | ENF | 40.03 | -105.55 | 3050 | 726 |
| US-Ne1 | United States | CRO | 41.17 | -96.48 | 361 | 838 |
| US-Ne2 | United States | CRO | 41.16 | -96.47 | 362 | 860 |
| US-Ne3 | United States | CRO | 41.18 | -96.44 | 363 | 697 |
| US-PFa | United States | MF | 45.95 | -90.27 | 470 | 606 |
| US-Prr | United States | ENF | 65.12 | -147.49 | 210 | 250 |
| US-SP1 | United States | ENF | 29.74 | -82.22 | 47 | 54 |
| US-SP2 | United States | ENF | 29.76 | -82.24 | 46 | 1068 |
| US-SP3 | United States | EBF | 29.75 | -82.16 | 36 | 992 |
| US-SRG | United States | GRA | 31.79 | -110.83 | 1291 | 371 |
| US-SRM | United States | SAV | 31.82 | -110.87 | 1120 | 333 |
| US-Syv | United States | MF | 46.24 | -89.35 | 540 | 654 |
| US-Ton | United States | SAV | 38.43 | -120.97 | 177 | 545 |
| US-Tw4 | United States | WET | 38.10 | -121.64 | -5 | 387 |
| US-Twt | United States | CRO | 38.11 | -121.65 | -7 | 359 |
| US-UMB | United States | DBF | 45.56 | -84.71 | 234 | 613 |
| US-Var | United States | GRA | 38.41 | -120.95 | 129 | 563 |
| US-WCr | United States | DBF | 45.81 | -90.08 | 520 | 751 |
| US-Whs | United States | SHR | 31.74 | -110.05 | 1370 | 284 |
| US-Wkg | United States | GRA | 31.74 | -109.94 | 1531 | 292 |
| ZA-Kru | South Africa | SAV | -25.02 | 31.50 | 359 | 676 |
| ZM-Mon | Zambia | DBF | -15.44 | 23.25 | 1053 | 681 |

**Supplementary Table 3. The depths of observed soil moisture (SM) and soil temperature (ST).**

| Site Name | Depth of observed SM (cm) | | | | | | | Depth of observed ST (cm) | | | | | | | | |
| --- | --- | --- | --- | --- | --- | --- | --- | --- | --- | --- | --- | --- | --- | --- | --- | --- |
| AR-SLu | 5 |  |  |  |  |  |  | 5 |  |  |  |  |  |  |  |  |
| AT-Neu | 5 | 10 | 20 |  |  |  |  | 5 | 10 | 20 |  |  |  |  |  |  |
| AU-ASM | 5 |  |  |  |  |  |  | 5 |  |  |  |  |  |  |  |  |
| AU-Cpr | 10 |  |  |  |  |  |  | 8 |  |  |  |  |  |  |  |  |
| AU-Cum | 8 |  |  |  |  |  |  | 8 |  |  |  |  |  |  |  |  |
| AU-DaP | 5 |  |  |  |  |  |  | 4 |  |  |  |  |  |  |  |  |
| AU-DaS | 5 |  |  |  |  |  |  | 4 |  |  |  |  |  |  |  |  |
| AU-Dry | 5 |  |  |  |  |  |  | 4 |  |  |  |  |  |  |  |  |
| AU-Emr | 5 |  |  |  |  |  |  | 3.5 |  |  |  |  |  |  |  |  |
| AU-GWW | 5 |  |  |  |  |  |  | 6 |  |  |  |  |  |  |  |  |
| AU-Gin | 10 |  |  |  |  |  |  | 10 |  |  |  |  |  |  |  |  |
| AU-How | 10 |  |  |  |  |  |  | 4 |  |  |  |  |  |  |  |  |
| AU-Rig | 5 |  |  |  |  |  |  | 4 |  |  |  |  |  |  |  |  |
| AU-Rob | 10 |  |  |  |  |  |  | 5 |  |  |  |  |  |  |  |  |
| AU-Stp | 50 |  |  |  |  |  |  | 4 |  |  |  |  |  |  |  |  |
| AU-TTE | 5 |  |  |  |  |  |  | 5 |  |  |  |  |  |  |  |  |
| AU-Tum | 15 |  |  |  |  |  |  | 2 |  |  |  |  |  |  |  |  |
| AU-Whr |  |  |  |  |  |  |  | 4 |  |  |  |  |  |  |  |  |
| AU-Ync | 3 |  |  |  |  |  |  | 3 |  |  |  |  |  |  |  |  |
| BE-Bra |  |  |  |  |  |  |  | 3.5 | 15 |  |  |  |  |  |  |  |
| BE-Lon | 5 | 15 | 25 |  |  |  |  | 3 | 6 | 9 | 26 | 56 |  |  |  |  |
| BE-Vie | 5 | 18 | 42 |  |  |  |  | 4 | 6.5 | 10 | 27 | 57 | 110 |  |  |  |
| BR-Sa3 | 15 |  |  |  |  |  |  | 10 |  |  |  |  |  |  |  |  |
| CA-NS1 |  |  |  |  |  |  |  | 10 |  |  |  |  |  |  |  |  |
| CA-NS2 |  |  |  |  |  |  |  | 10 |  |  |  |  |  |  |  |  |
| CA-NS4 |  |  |  |  |  |  |  | 10 |  |  |  |  |  |  |  |  |
| CA-NS5 |  |  |  |  |  |  |  | 10 |  |  |  |  |  |  |  |  |
| CA-NS6 |  |  |  |  |  |  |  | 10 |  |  |  |  |  |  |  |  |
| CA-NS7 |  |  |  |  |  |  |  | 10 |  |  |  |  |  |  |  |  |
| CA-Qfo | 5 |  |  |  |  |  |  | 2 |  |  |  |  |  |  |  |  |
| CA-SF1 | 30 |  |  |  |  |  |  | 2 |  |  |  |  |  |  |  |  |
| CA-SF2 | 15 |  |  |  |  |  |  | 2 |  |  |  |  |  |  |  |  |
| CA-SF3 | 15 |  |  |  |  |  |  | 2 |  |  |  |  |  |  |  |  |
| CH-Cha | 5 | 15 | 25 |  |  |  |  | 1 | 2 | 4 | 7 | 10 | 15 | 25 | 40 | 95 |
| CH-Dav | 5 | 15 | 45 |  |  |  |  | 5 | 15 | 50 |  |  |  |  |  |  |
| CH-Fru | 5 | 15 | 25 | 40 |  |  |  | 1 | 2 | 4 | 7 | 10 | 15 | 25 | 40 | 95 |
| CH-Oe1 | 5 | 10 | 30 |  |  |  |  | 5 | 10 | 30 |  |  |  |  |  |  |
| CN-Cha | 10 |  |  |  |  |  |  | 5 |  |  |  |  |  |  |  |  |
| CN-Cng | 10 |  |  |  |  |  |  | 5 |  |  |  |  |  |  |  |  |
| CN-Dan | 5 |  |  |  |  |  |  | 5 |  |  |  |  |  |  |  |  |
| CN-Din | 5 |  |  |  |  |  |  | 2 |  |  |  |  |  |  |  |  |
| CN-Du2 | 10 |  |  |  |  |  |  | 5 |  |  |  |  |  |  |  |  |
| CN-HaM | 5 |  |  |  |  |  |  | 2 |  |  |  |  |  |  |  |  |
| CN-Qia | 5 |  |  |  |  |  |  | 5 |  |  |  |  |  |  |  |  |
| CZ-wet |  |  |  |  |  |  |  | 1 | 5 | 10 | 20 | 30 |  |  |  |  |
| DE-Geb | 8 | 16 | 32 | 64 |  |  |  | 2 | 4 | 8 | 16 | 32 | 64 |  |  |  |
| DE-Gri | 10 |  |  |  |  |  |  | 2 | 5 | 10 | 20 | 50 |  |  |  |  |
| DE-Hai | 8 | 16 | 32 |  |  |  |  | 2 | 5 | 15 | 30 | 50 |  |  |  |  |
| DE-Kli | 10 |  |  |  |  |  |  | 2 | 5 | 10 |  |  |  |  |  |  |
| DE-Obe | 10 | 20 | 35 |  |  |  |  | 2 | 5 | 10 | 20 | 50 |  |  |  |  |
| DE-Seh | 1 | 3 |  |  |  |  |  | 5 |  |  |  |  |  |  |  |  |
| DE-SfN |  |  |  |  |  |  |  | 2 | 5 | 10 | 20 | 50 |  |  |  |  |
| DE-Tha | 10 |  |  |  |  |  |  | 2 | 5 | 10 | 20 | 35 | 50 |  |  |  |
| DK-Fou | 10 | 25 | 50 |  |  |  |  | 2 | 5 | 10 | 30 |  |  |  |  |  |
| DK-Sor | 3 | 5 | 10 | 20 | 40 | 80 |  | 5 | 10 | 20 | 40 | 80 |  |  |  |  |
| ES-LgS | 4 |  |  |  |  |  |  | 4 |  |  |  |  |  |  |  |  |
| FI-Hyy | 2 | 4 | 16 | 30 | 50 |  |  | 2 | 4 | 12 | 25 | 50 |  |  |  |  |
| FI-Lom |  |  |  |  |  |  |  | 7 | 15 | 30 |  |  |  |  |  |  |
| FI-Sod | 5 | 30 |  |  |  |  |  | 5 | 30 |  |  |  |  |  |  |  |
| FR-Gri | 5 | 10 | 20 | 30 | 50 | 70 | 90 | 1 | 5 | 10 | 20 | 30 | 50 | 70 | 90 | 90 |
| FR-LBr | 15 | 30 | 45 | 60 | 75 | 90 |  | 4 | 32 | 82 |  |  |  |  |  |  |
| FR-Pue |  |  |  |  |  |  |  | 15 |  |  |  |  |  |  |  |  |
| GF-Guy | 5 | 10 | 20 | 26 |  |  |  | 5 | 53 |  |  |  |  |  |  |  |
| IT-BCi | 3.5 | 7 |  |  |  |  |  | 5 | 10 | 15 | 30 |  |  |  |  |  |
| IT-CA1 | 5 | 15 |  |  |  |  |  | 5 | 15 | 30 | 80 |  |  |  |  |  |
| IT-CA2 | 5 | 15 | 35 |  |  |  |  | 5 | 15 | 30 |  |  |  |  |  |  |
| IT-CA3 | 5 | 15 | 35 |  |  |  |  | 5 | 15 | 35 | 100 |  |  |  |  |  |
| IT-Col | 1 | 5 | 20 | 50 |  |  |  | 5 | 20 |  |  |  |  |  |  |  |
| IT-Cpz | 10 | 30 |  |  |  |  |  | 5 | 30 | 20 |  |  |  |  |  |  |
| IT-Isp | 10 | 30 | 50 | 100 |  |  |  | 5 | 10 | 20 | 30 | 50 | 100 |  |  |  |
| IT-Lav | 10 | 20 |  |  |  |  |  | 10 | 20 | 50 |  |  |  |  |  |  |
| IT-MBo | 10 | 20 |  |  |  |  |  | 2 | 5 | 10 |  |  |  |  |  |  |
| IT-Noe | 30 | 35 |  |  |  |  |  | 5 |  |  |  |  |  |  |  |  |
| IT-PT1 | 15 | 50 |  |  |  |  |  | 5 | 20 | 40 |  |  |  |  |  |  |
| IT-Ren | 15 | 16 |  |  |  |  |  | 2 | 5 | 10 | 20 | 50 |  |  |  |  |
| IT-Ro1 | 10 | 30 |  |  |  |  |  | 5 | 15 | 30 |  |  |  |  |  |  |
| IT-Ro2 | 10 | 30 |  |  |  |  |  | 5 | 15 | 30 |  |  |  |  |  |  |
| IT-SR2 | 5 | 30 | 50 | 100 |  |  |  | 5 | 10 | 20 | 30 | 50 | 100 |  |  |  |
| IT-SRo | 10 | 50 |  |  |  |  |  | 3 | 15 | 50 |  |  |  |  |  |  |
| JP-SMF | 2 |  |  |  |  |  |  | 2 |  |  |  |  |  |  |  |  |
| NL-Hor |  |  |  |  |  |  |  | 1 | 2 | 4 | 5 | 10 | 15 | 25 | 40 | 60 |
| NL-Loo | 10 | 25 | 200 |  |  |  |  | 10 | 25 | 100 |  |  |  |  |  |  |
| RU-Che |  |  |  |  |  |  |  | 1 | 5 | 10 |  |  |  |  |  |  |
| RU-Fyo |  |  |  |  |  |  |  | 5 | 15 | 50 |  |  |  |  |  |  |
| SD-Dem | 5 | 15 | 30 | 60 | 100 | 150 | 200 | 3 | 3.5 | 30 | 60 |  |  |  |  |  |
| US-AR1 | 10 |  |  |  |  |  |  | 5 |  |  |  |  |  |  |  |  |
| US-AR2 | 10 |  |  |  |  |  |  | 5 |  |  |  |  |  |  |  |  |
| US-ARM | 10 |  |  |  |  |  |  | 5 |  |  |  |  |  |  |  |  |
| US-Blo | 10 |  |  |  |  |  |  | 5 |  |  |  |  |  |  |  |  |
| US-Cop | 10 |  |  |  |  |  |  | 10 |  |  |  |  |  |  |  |  |
| US-GLE | 5 |  |  |  |  |  |  | 3 |  |  |  |  |  |  |  |  |
| US-Goo | 5 |  |  |  |  |  |  | 2 |  |  |  |  |  |  |  |  |
| US-Ha1 |  |  |  |  |  |  |  | 10 |  |  |  |  |  |  |  |  |
| US-KS2 | 7.5 |  |  |  |  |  |  | 1 |  |  |  |  |  |  |  |  |
| US-Los |  |  |  |  |  |  |  | 20 |  |  |  |  |  |  |  |  |
| US-MMS | 15 |  |  |  |  |  |  | 5 |  |  |  |  |  |  |  |  |
| US-Me2 | 10 |  |  |  |  |  |  | 2 |  |  |  |  |  |  |  |  |
| US-Me4 | 30 |  |  |  |  |  |  | 2 |  |  |  |  |  |  |  |  |
| US-Me6 |  |  |  |  |  |  |  | 2 |  |  |  |  |  |  |  |  |
| US-Myb |  |  |  |  |  |  |  | 8 |  |  |  |  |  |  |  |  |
| US-NR1 | 5 |  |  |  |  |  |  | 5 |  |  |  |  |  |  |  |  |
| US-Ne1 | 10 |  |  |  |  |  |  | 2 |  |  |  |  |  |  |  |  |
| US-Ne2 | 10 |  |  |  |  |  |  | 2 |  |  |  |  |  |  |  |  |
| US-Ne3 | 10 |  |  |  |  |  |  | 2 |  |  |  |  |  |  |  |  |
| US-Prr |  |  |  |  |  |  |  | 5 |  |  |  |  |  |  |  |  |
| US-SRG | 5 |  |  |  |  |  |  | 4 |  |  |  |  |  |  |  |  |
| US-SRM | 5 |  |  |  |  |  |  | 5 |  |  |  |  |  |  |  |  |
| US-Syv | 10 |  |  |  |  |  |  | 5 |  |  |  |  |  |  |  |  |
| US-Ton | 2 |  |  |  |  |  |  | 2 |  |  |  |  |  |  |  |  |
| US-Tw4 |  |  |  |  |  |  |  | 8 |  |  |  |  |  |  |  |  |
| US-Twt |  |  |  |  |  |  |  | 2 |  |  |  |  |  |  |  |  |
| US-UMB | 2 |  |  |  |  |  |  | 2 |  |  |  |  |  |  |  |  |
| US-Var | 2 |  |  |  |  |  |  | 2 |  |  |  |  |  |  |  |  |
| US-WCr | 5 |  |  |  |  |  |  | 5 |  |  |  |  |  |  |  |  |
| US-Whs | 5 |  |  |  |  |  |  | 5 |  |  |  |  |  |  |  |  |
| US-Wkg | 5 |  |  |  |  |  |  | 5 |  |  |  |  |  |  |  |  |
| ZA-Kru | 5 | 10 | 30 | 60 |  |  |  |  |  |  |  |  |  |  |  |  |
| ZM-Mon | 5 | 10 | 30 |  |  |  |  | 5 | 10 | 30 |  |  |  |  |  |  |

**Supplementary Table 4. Parameters used in STEMMUS-SCOPE for the different vegetation types.**

| Vege_type | OSHR | CSHR | CRO | | DBF | EBF | ENF | GRA | | MF | WET | SAV | WSAV |
| --- | --- | --- | --- | --- | --- | --- | --- | --- | --- | --- | --- | --- | --- |
| Photo_Path | C3 | C3 | C3 | C4 | C3 | C3 | C3 | C3 | C4 | C3 | C3 | C3 | C3 |
| Slti | 0.2 | 0.2 | 0.2 | 0.2 | 0.2 | 0.2 | 0.2 | 0.2 | 0.2 | 0.2 | 0.2 | 0.2 | 0.2 |
| Shti | 0.3 | 0.3 | 0.3 | 0.3 | 0.3 | 0.3 | 0.3 | 0.3 | 0.3 | 0.3 | 0.3 | 0.3 | 0.3 |
| Thl (K) | 288 | 288 | 278 | 278 | 283 | 283 | 278 | 288 | 288 | 281 | 288 | 278 | 278 |
| Thh (K) | 313 | 313 | 303 | 303 | 311 | 313 | 303 | 303 | 303 | 307 | 313 | 313 | 313 |
| Trdm (K) | 328 | 328 | 328 | 328 | 328 | 328 | 328 | 328 | 328 | 328 | 328 | 328 | 328 |
| Vcmax (µmol m^-2^ s^-1^) | 120 | 80 | 120 | 50 | 120 | 80 | 120 | 120 | 50 | 120 | 120 | 120 | 120 |
| Cab (ug cm^-2^) | 120 | 120 | 120 | 120 | 120 | 120 | 120 | 120 | 120 | 120 | 120 | 120 | 120 |
| m | 9 | 9 | 9 | 4 | 9 | 9 | 9 | 9 | 4 | 9 | 9 | 9 | 9 |
| BallBerry0 | 0.015 | 0.015 | 0.015 | 0.015 | 0.015 | 0.015 | 0.015 | 0.015 | 0.015 | 0.015 | 0.015 | 0.015 | 0.015 |
| Rdparam | 0.015 | 0.015 | 0.015 | 0.025 | 0.015 | 0.015 | 0.015 | 0.015 | 0.025 | 0.015 | 0.015 | 0.015 | 0.015 |
| LIDFa | -0.35 | -0.35 | -0.35 | -0.35 | -0.35 | -0.35 | -0.35 | -0.35 | -0.35 | -0.35 | -0.35 | -0.35 | -0.35 |
| LIDFb | -0.15 | -0.15 | -0.15 | -0.15 | -0.15 | -0.15 | -0.15 | -0.15 | -0.15 | -0.15 | -0.15 | -0.15 | -0.15 |
| Leafwidth (m) | 0.05 | 0.05 | 0.03 | 0.03 | 0.05 | 0.05 | 0.01 | 0.02 | 0.02 | 0.04 | 0.05 | 0.05 | 0.03 |
| $\beta$ | 0.966 | 0.966 | 0.943 | 0.943 | 0.993 | 0.993 | 0.993 | 0.943 | 0.943 | 0.993 | 0.993 | 0.943 | 0.943 |

Vege_type and Photo_Path means vegetation type and photochemical pathway, respectively. Vcmax is the maximum carboxylation rate at 25 ^o^ C. Cab is the chlorophyll content. m is the slope of Ball-Berry equation. BallBerry0 is the intercept of Ball-Berry equation. Rdparam is the leaf respiration parameter. LIDFa and LIDFb is the parameter a and parameter b of the leaf inclination distribution function, respectively. leafwidth is the leaf width. $\beta$ is the plant-dependent root distribution parameter.

Slti is the slope of cold temperature decline (C4 only). Shti is the slope of high-temperature decline in photosynthesis. Thl is the temperature below which C4 photosynthesis is lower than half that predicted by Q10. Thh is the temperature above which photosynthesis is lower than half that predicted by Q10. Trdm is the temperature at which respiration is lower than half that predicted by Q10.

OSHR and CSHR is Open Shrublands and Closed Shrubland, respectively. CRO is Croplands. DBF is Deciduous Broadleaf Forests. EBF is Evergreen Broadleaf Forests. ENF is Evergreen Needleleaf Forests. GRA is Grasslands. MF is Mixed Forests. WET is Wetlands. SAV and WSAV is Savannas and Woody Savannas, respectively.

**Supplementary Table 5. Criteria for classification of 170 sites.**

|  | Excellent | Good | Average | Poor |
| --- | --- | --- | --- | --- |
| Fluxes | All the KGE values of LE, H, and GPP are higher than 0.4 | Two KGE values of LE, H, and GPP are higher than 0 | One KGE of LE, H, and GPP are higher than -0.4 | All KGE values of LE, H, and GPP are lower than -0.4 |
| SM | KGE >0.4 | 0.4>KGE >0 | 0>KGE >-0.4 | KGE<-0.4 |

**Supplementary Table 6. Maximum, 75th percentiles, Median, 25th percentiles, and Minimum values of KGE. (Unit of RMSE: Rn: net radiation, W m^-2^; LE: latent heat flux, W m^-2^; H: sensible heat flux, W m^-2^; G: ground heat flux, W m^-2^; GPP: gross primary production, µmol m-2 s^-1^; NEE: net ecosystem exchange, *µ*mol m^-2^ s^-1^; SM: soil moisture, % m^3^ m^-3^; ST: soil temperature, ^o^C)**

|  |  | Rn | LE | H | G | GPP | NEE | SM | ST |
| --- | --- | --- | --- | --- | --- | --- | --- | --- | --- |
| KGE | Maximum | 0.99 | 0.90 | 0.88 | 0.58 | 0.93 | 0.84 | 0.93 | 0.96 |
|  | 75th (q75) percentiles | 0.89 | 0.71 | 0.52 | -1.34 | 0.72 | 0.52 | 0.46 | 0.76 |
|  | Median | 0.80 | 0.60 | 0.30 | -3.52 | 0.55 | -0.03 | 0.24 | 0.56 |
|  | 25th (q25) percentiles | 0.67 | 0.35 | -0.08 | -7.46 | 0.29 | -1.00 | -0.03 | 0.34 |
|  | Minimum | 0.35 | -0.13 | -0.91 | -15.88 | -0.35 | -3.28 | -0.73 | -0.31 |
| R^2^ | Maximum | 1.00 | 0.92 | 0.92 | 0.81 | 0.89 | 0.86 | 0.95 | 0.99 |
|  | 75th (q75) percentiles | 0.99 | 0.7 | 0.79 | 0.43 | 0.76 | 0.71 | 0.62 | 0.92 |
|  | Median | 0.97 | 0.63 | 0.68 | 0.22 | 0.64 | 0.54 | 0.46 | 0.88 |
|  | 25th (q25) percentiles | 0.95 | 0.54 | 0.58 | 0.08 | 0.48 | 0.39 | 0.18 | 0.82 |
|  | Minimum | 0.9 | 0.31 | 0.32 | 0.00 | 0.07 | 0.00 | 0.00 | 0.67 |
| RMSE | Maximum | 76.7 | 79.9 | 95.0 | 60.7 | 8.96 | 8.69 | 21.22 | 7.27 |
|  | 75th (q75) percentiles | 51.6 | 55.0 | 70.2 | 40.7 | 5.29 | 5.42 | 12.33 | 4.49 |
|  | Median | 38.6 | 46.2 | 58.1 | 32.8 | 3.79 | 4.00 | 8.84 | 3.54 |
|  | 25th (q25) percentiles | 28.8 | 38.3 | 50.7 | 26.8 | 2.81 | 3.20 | 5.86 | 2.60 |
|  | Minimum | 10.2 | 17.6 | 32.6 | 13.3 | 0.64 | 1.51 | 1.7 | 0.85 |
| rRMSE | Maximum | 8.76 | 12.39 | 15.04 | 69.67 | 12.89 | 14.17 | 68.93 | 40.39 |
|  | 75th (q75) percentiles | 5.54 | 8.82 | 10.5 | 34.72 | 8.30 | 9.05 | 41.28 | 21.67 |
|  | Median | 4.09 | 7.29 | 8.49 | 19.12 | 6.15 | 7.09 | 29.11 | 13.8 |
|  | 25th (q25) percentiles | 2.90 | 6.16 | 7.01 | 9.17 | 5.09 | 5.61 | 19.1 | 8.95 |
|  | Minimum | 0.95 | 3.56 | 4.82 | 4.17 | 1.40 | 1.40 | 5.80 | 4.24 |
| rSD | Maximum | 0.28 | 0.66 | 1.02 | 5.34 | 0.75 | 0.84 | 1.06 | 1.19 |
|  | 75th (q75) percentiles | 0.13 | 0.32 | 0.48 | 2.40 | 0.36 | 0.40 | 0.54 | 0.60 |
|  | Median | 0.07 | 0.17 | 0.26 | 1.18 | 0.18 | 0.20 | 0.34 | 0.39 |
|  | 25th (q25) percentiles | 0.02 | 0.08 | 0.12 | 0.31 | 0.08 | 0.10 | 0.18 | 0.20 |
|  | Minimum | 0.00 | 0.00 | 0.00 | 0.01 | 0.00 | 0.00 | 0.00 | 0.00 |

**Supplementary Table 7. Description of the performance of 170 sites.**

| Site Name | Vege_type | Fluxes | SM | Site Name | Vege_type | Fluxes | SM |
| --- | --- | --- | --- | --- | --- | --- | --- |
| AR-SLu | MF | Average | Good | CN-Cng | GRA | Good | Average |
| AT-Neu | GRA | Average | Good | CN-Dan | GRA | Average | Good |
| AU-ASM | ENF | Average | Good | CN-Din | EBF | Poor | Excellent |
| AU-Cow | EBF | Average | Unvalidated | CN-Du2 | GRA | Good | Average |
| AU-Cpr | SAV | Good | Poor | CN-HaM | GRA | Poor | Average |
| AU-Ctr | EBF | Average | Unvalidated | CN-Qia | ENF | Good | Good |
| AU-Cum | EBF | Excellent | Excellent | CZ-wet | WET | Good | Unvalidated |
| AU-DaP | GRA | Good | Average | DE-Bay | ENF | Good | Unvalidated |
| AU-DaS | SAV | Excellent | Poor | DE-Geb | CRO | Average | Excellent |
| AU-Dry | SAV | Average | Good | DE-Gri | GRA | Average | Excellent |
| AU-Emr | GRA | Poor | Good | DE-Hai | DBF | Good | Excellent |
| AU-GWW | SAV | Average | Good | DE-Kli | CRO | Poor | Good |
| AU-Gin | SAV | Good | Poor | DE-Meh | MF | Good | Unvalidated |
| AU-How | SAV | Excellent | Good | DE-Obe | ENF | Good | Good |
| AU-Lit | SAV | Average | Unvalidated | DE-Seh | CRO | Good | Excellent |
| AU-Otw | GRA | Average | Unvalidated | DE-SfN | WET | Good | Unvalidated |
| AU-Rig | GRA | Excellent | Excellent | DE-Tha | ENF | Good | Good |
| AU-Rob | EBF | Average | Excellent | DE-Wet | ENF | Good | Unvalidated |
| AU-Sam | GRA | Good | Unvalidated | DK-Fou | CRO | Poor | Excellent |
| AU-Stp | GRA | Good | Good | DK-Lva | GRA | Good | Unvalidated |
| AU-TTE | SHR | Average | Poor | DK-Ris | CRO | Good | Unvalidated |
| AU-Tum | EBF | Excellent | Good | DK-Sor | DBF | Average | Excellent |
| AU-Whr | EBF | Good | Unvalidated | DK-ZaH | GRA | Average | Unvalidated |
| AU-Wrr | EBF | Average | Unvalidated | ES-ES1 | ENF | Excellent | Unvalidated |
| AU-Ync | GRA | Average | Average | ES-ES2 | CRO | Average | Unvalidated |
| BE-Bra | MF | Average | Unvalidated | ES-LMa | SAV | Excellent | Unvalidated |
| BE-Lon | CRO | Good | Excellent | ES-LgS | SHR | Average | Excellent |
| BE-Vie | MF | Good | Excellent | ES-VDA | GRA | Average | Unvalidated |
| BR-Sa3 | EBF | Average | Excellent | FI-Hyy | ENF | Average | Good |
| BW-Ma1 | SAV | Good | Unvalidated | FI-Kaa | WET | Good | Unvalidated |
| CA-NS1 | ENF | Average | Excellent | FI-Lom | WET | Good | Unvalidated |
| CA-NS2 | ENF | Excellent | Average | FI-Sod | ENF | Poor | Poor |
| CA-NS4 | ENF | Poor | Good | FR-Fon | DBF | Good | Unvalidated |
| CA-NS5 | ENF | Good | Average | FR-Gri | CRO | Average | Excellent |
| CA-NS6 | SHR | Good | Average | FR-Hes | DBF | Average | Unvalidated |
| CA-NS7 | SHR | Good | Average | FR-LBr | ENF | Good | Good |
| CA-Qcu | ENF | Good | Unvalidated | FR-Lq1 | GRA | Good | Unvalidated |
| CA-Qfo | ENF | Average | Poor | FR-Lq2 | GRA | Good | Unvalidated |
| CA-SF1 | ENF | Good | Average | FR-Pue | EBF | Good | Unvalidated |
| CA-SF2 | ENF | Excellent | Poor | GF-Guy | EBF | Good | Good |
| CA-SF3 | SHR | Average | Average | HU-Bug | GRA | Excellent | Unvalidated |
| CH-Cha | GRA | Good | Excellent | ID-Pag | EBF | Excellent | Unvalidated |
| CH-Dav | ENF | Good | Good | IE-Ca1 | CRO | Average | Unvalidated |
| CH-Fru | GRA | Good | Good | IE-Dri | GRA | Excellent | Unvalidated |
| CH-Oe1 | GRA | Average | Excellent | IT-Amp | GRA | Excellent | Unvalidated |
| CN-Cha | MF | Excellent | Average | IT-BCi | CRO | Average | Excellent |

**Continue to Supplementary Table 7. Description of the performance of 170 sites.**

| Site Name | Vege_type | Fluxes | SM | Site Name | Vege_type | Fluxes | SM |
| --- | --- | --- | --- | --- | --- | --- | --- |
| IT-CA1 | DBF | Excellent | Excellent | US-Bkg | GRA | Average | Unvalidated |
| IT-CA2 | CRO | Good | Excellent | US-Blo | ENF | Excellent | Excellent |
| IT-CA3 | DBF | Excellent | Excellent | US-Bo1 | CRO | Good | Unvalidated |
| IT-Col | DBF | Good | Excellent | US-Cop | GRA | Poor | Excellent |
| IT-Cpz | EBF | Excellent | Good | US-FPe | GRA | Good | Unvalidated |
| IT-Isp | DBF | Average | Excellent | US-GLE | ENF | Good | Average |
| IT-LMa | DBF | Good | Unvalidated | US-Goo | GRA | Good | Good |
| IT-Lav | ENF | Excellent | Excellent | US-Ha1 | DBF | Average | Unvalidated |
| IT-MBo | GRA | Average | Good | US-Ho1 | ENF | Average | Unvalidated |
| IT-Mal | GRA | Good | Unvalidated | US-KS2 | SHR | Excellent | Average |
| IT-Noe | SHR | Excellent | Excellent | US-Los | WET | Poor | Unvalidated |
| IT-Non | DBF | Good | Unvalidated | US-MMS | DBF | Poor | Excellent |
| IT-PT1 | DBF | Good | Excellent | US-MOz | DBF | Good | Unvalidated |
| IT-Ren | ENF | Good | Good | US-Me2 | ENF | Excellent | Excellent |
| IT-Ro1 | DBF | Good | Excellent | US-Me4 | ENF | Average | Excellent |
| IT-Ro2 | DBF | Excellent | Excellent | US-Me6 | ENF | Average | Average |
| IT-SR2 | ENF | Excellent | Good | US-Myb | WET | Poor | Unvalidated |
| IT-SRo | ENF | Excellent | Good | US-NR1 | ENF | Average | Poor |
| JP-SMF | MF | Average | Poor | US-Ne1 | CRO | Average | Average |
| NL-Ca1 | GRA | Good | Unvalidated | US-Ne2 | CRO | Average | Good |
| NL-Hor | GRA | Average | Unvalidated | US-Ne3 | CRO | Average | Good |
| NL-Loo | ENF | Good | Average | US-PFa | MF | Good | Average |
| PL-wet | WET | Excellent | Unvalidated | US-Prr | ENF | Average | Average |
| PT-Esp | EBF | Excellent | Unvalidated | US-SP1 | ENF | Excellent | Unvalidated |
| PT-Mi1 | EBF | Good | Unvalidated | US-SP2 | ENF | Excellent | Unvalidated |
| PT-Mi2 | GRA | Excellent | Unvalidated | US-SP3 | EBF | Good | Unvalidated |
| RU-Che | WET | Average | Unvalidated | US-SRG | GRA | Good | Average |
| RU-Fyo | ENF | Average | Average | US-SRM | SAV | Good | Poor |
| RU-Zot | SAV | Good | Unvalidated | US-Syv | MF | Poor | Average |
| SD-Dem | SAV | Average | Good | US-Ton | SAV | Good | Excellent |
| SE-Deg | GRA | Good | Unvalidated | US-Tw4 | WET | Poor | Unvalidated |
| UK-Gri | ENF | Excellent | Unvalidated | US-Twt | CRO | Poor | Unvalidated |
| UK-Ham | DBF | Average | Unvalidated | US-UMB | DBF | Average | Poor |
| UK-PL3 | MF | Good | Unvalidated | US-Var | GRA | Average | Excellent |
| US-AR1 | GRA | Average | Excellent | US-WCr | DBF | Average | Good |
| US-AR2 | GRA | Average | Poor | US-Whs | SHR | Average | Poor |
| US-ARM | CRO | Good | Good | US-Wkg | GRA | Poor | Good |
| US-Aud | GRA | Average | Unvalidated | ZA-Kru | SAV | Average | Good |
| US-Bar | MF | Average | Unvalidated | ZM-Mon | DBF | Excellent | Excellent |

(Note: SHR is (Open/Closed) Shrublands. CRO is Croplands. DBF is Deciduous Broadleaf Forests. EBF is Evergreen Broadleaf Forests. ENF is Evergreen Needleleaf Forests. GRA is Grasslands. MF is Mixed Forests. WET is Permanent Wetlands. SAV is (Woody) Savannas)

**Supplementary Table 8.**  **Criteria for classification of water status based on mean water stress factor (WSF).**

| 1>WSF>0.8 | 0.8>WSF>0.6 | 0.6>WSF>0.4 | 0.4>WSF |
| --- | --- | --- | --- |
| Wet | Mild | Dry | Severe |

# References

1 Farquhar, G. D., von Caemmerer, S. & Berry, J. A. A biochemical model of photosynthetic CO2 assimilation in leaves of C 3 species. *Planta* **149**, 78-90 (1980). https://doi.org/10.1007/BF00386231

2 Bayat, B., van der Tol, C., Yang, P. Q. & Verhoef, W. Extending the SCOPE model to combine optical reflectance and soil moisture observations for remote sensing of ecosystem functioning under water stress conditions. *REMOTE SENSING OF ENVIRONMENT* **221**, 286-301 (2019). https://doi.org/10.1016/j.rse.2018.11.021

3 Zeng, Y., Su, Z., Wan, L. & Wen, J. Numerical analysis of air-water-heat flow in unsaturated soil: Is it necessary to consider airflow in land surface models? *Journal of Geophysical Research* **116** (2011). https://doi.org/10.1029/2011jd015835

4 Zeng, Y., Su, Z., Wan, L. & Wen, J. A simulation analysis of the advective effect on evaporation using a two-phase heat and mass flow model. *Water Resources Research* **47** (2011). https://doi.org/10.1029/2011wr010701

5 Jackson, R. B. *et al.* A global analysis of root distributions for terrestrial biomes. *Oecologia* **108**, 389-411 (1996). https://doi.org/10.1007/BF00333714

6 Wang, Y. *et al.* Integrated modeling of canopy photosynthesis, fluorescence, and the transfer of energy, mass, and momentum in the soil–plant–atmosphere continuum (STEMMUS–SCOPE v1.0.0). *Geoscientific Model Development* **14**, 1379-1407 (2021). https://doi.org/10.5194/gmd-14-1379-2021

7 van Genuchten, M. T. A Closed-form Equation for Predicting the Hydraulic Conductivity of Unsaturated Soils. *Soil Science Society of America Journal* **44**, 892-898 (1980). https://doi.org/https://doi.org/10.2136/sssaj1980.03615995004400050002x

8 Reid, J. & Huck, M. Diurnal variation of crop hydraulic resistance: a new analysis. *Agronomy journal* **82**, 827-834 (1990).

9 Klepper, B., Rickman, R. W. & Taylor, H. M. Farm management and the function of field crop root systems. *Agricultural Water Management* **7**, 115-141 (1983). https://doi.org/https://doi.org/10.1016/0378-3774(83)90078-1
